# Cationic Surfactants Based on Arginine-Phenylalanine and Arginine-Tryptophan: Synthesis, Aggregation Behavior, Antimicrobial Activity, and Biodegradation

**DOI:** 10.3390/pharmaceutics14122602

**Published:** 2022-11-25

**Authors:** Lourdes Pérez, María Teresa García, Aurora Pinazo, Edgar Pérez-Matas, Zakaria Hafidi, Elena Bautista

**Affiliations:** Department of Surfactants and Nanobiotechnology, Institute for Advanced Chemistry of Catalonia (IQAC-CSIC), c/Jordi Girona, 18-26, 08034 Barcelona, Spain

**Keywords:** arginine phenylalanine tryptophan, surfactants, antimicrobial activity, biodegradation

## Abstract

Cationic surfactants have great potential as drug vehicles and for use in gene therapy (cationic vesicles made from cationic surfactants can encapsulate RNA or DNA for cellular transfer). They can also be used as antimicrobial and antifungal agents to treat human infections. In an era of increasing antimicrobial resistance, the development of new biocompatible surfactants suitable for application as antimicrobial agents is of high interest. In this work, a library of amino acid-based surfactants was synthesized, characterized and tested for antimicrobial activity. The head group architecture (number and type of amino acids, density of cationic charge, ionic character) and the hydrophobic moiety (alkyl chain length and position of the hydrophobic group) were systematically modified, and the effect on the surfactant biological and aggregation behavior was studied. Thus, the pKa values, micellization process, antimicrobial efficiency and biodegradability were evaluated. The critical micelle concentration values of the surfactants depended on their hydrophobic character, but changes in the polar head as well as the position and length of the alkyl chain also significantly affected activity against some of the tested microorganisms. Moreover, biodegradability was closely related to the hydrophobic character of the surfactant and attachment of the alkyl chain to the polar head. The structure–activity relationships established here may open perspectives for the design of effective biodegradable antimicrobial materials that can overcome emerging resistance.

## 1. Introduction

Infectious diseases are a major concern for human and veterinary health and therefore for the global economy [1]. An outcome of the mass usage of antibiotics and biocides in the last century is the rapid evolution of highly resistant microorganisms, including bacteria, fungi, viruses, and parasites, which is threatening our ability to treat common infections. The World Health Organization (WHO) recognizes that infections caused by multi-drug-resistant pathogens are one of the top three threats to global health [2], predicted to cause 10 million deaths per year by 2050 if no action is taken [3]. In May 2015, the 68th World Health Assembly adopted the Global Action Plan on Antimicrobial Resistance [4], with member states proposing specific actions to tackle the problem. These include raising public awareness about the negative effects of antibiotic misuse and stimulating research on novel antimicrobial agents that can prevent the spread of infectious disease.

It has been demonstrated that conventional antimicrobials easily, and sometimes quickly, lose efficacy due to microbial resistance [5,6] and biocidal diffusion can cause environmental contamination and toxicity for humans [7]. Vancomycin-resistant enterococci and methicillin-resistant *Staphylococcus aureus* can survive for a day on materials used in healthcare systems, while other microbes can endure for 90 days [8]. Microbial cells attached to any artificial surface in a moist environment can proliferate and form a biofilm, a polysaccharide matrix in which the cells are embedded. In this way, microbial cells can withstand harsh conditions and are up to 1000 times less susceptible to most antibiotics and biocides [9]. Biofilms are the cause of nosocomial, recurrent, and chronic infections associated with catheters, implants, or prostheses, and often trigger food-associated outbreaks [10]. Antimicrobial treatment may kill metabolically active cells in biofilms, but without affecting persister cells, which can re-start colonization and biofilm development when the treatment is removed [11]. There is therefore a pressing need for new more efficient broad-spectrum antibacterial agents that not only disarm bacterial resistance but also eradicate bacterial biofilms.

Conventional quaternary ammonium compounds (QACs) are cationic amphiphilic molecules with broad-spectrum antimicrobial activity, widely used as disinfectants and antiseptics for general hygiene and clinical purposes [12]. QACs exert their biological activity mainly by inducing disintegration of bacterial membranes via electrostatic and hydrophobic interactions [13]. Although it is difficult for bacteria to circumvent this non-specific disruption mechanism, the huge consumption of QACs worldwide and their ensuing accumulation in the environment is applying selective pressure on bacteria and triggering the emergence of antimicrobial resistance [14]. Another reason why the use of QACs is being increasingly questioned is their persistence in the environment, due to chemical stability and low biodegradability. Negative environmental impacts of QACs include disruption of wastewater treatment unit operations, proliferation of antimicrobial resistance, toxicity for aquatic species and alterations in the biota of surface water [15]. In Europe, limitations have been recently imposed on QAC usage in food products and consumer hand and body washes [16]. The various drawbacks of QACs call for the development of new biodegradable molecules that meet safety and efficacy requirements for use as antimicrobials in antiseptic formulations and in the development of biomedical material with antimicrobial properties.

Cationic amino acid-based surfactants represent a promising starting point for the development of alternatives to the increasingly controversial QACs. Amino acid-based surfactants consist of one or two amino acids linked to a hydrophobic moiety. Like QACs, they act via perturbation of the bacterial membrane [17,18], but as they can be designed to biodegrade in the environment, their capacity to induce bacterial resistance is expected to be low. Taking advantage of the great variety of existing amino acids, the physicochemical and biological properties of this type of surfactants can be tailored through structural modifications.

**Figure 1:**
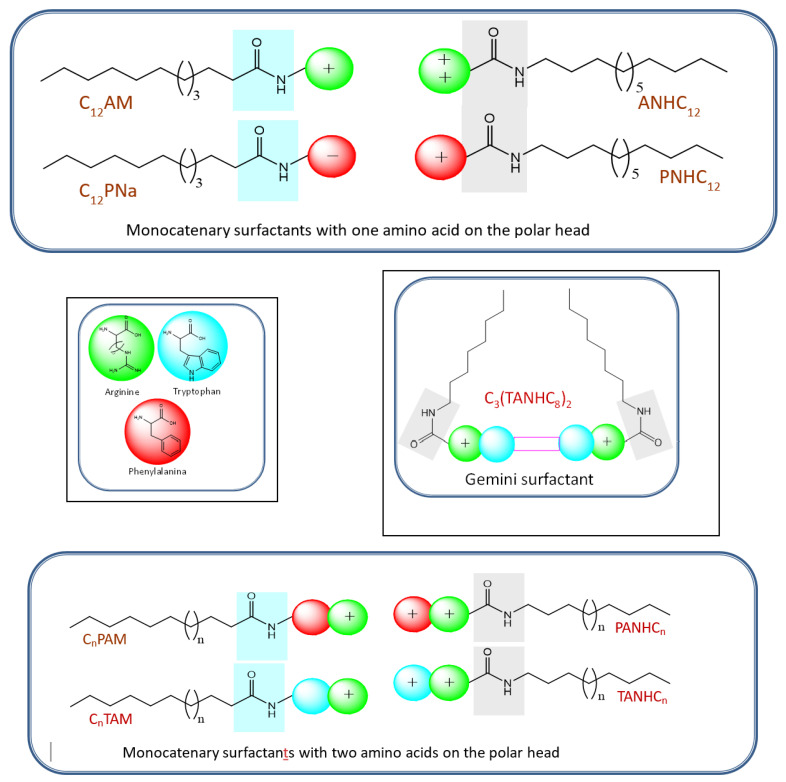
Structure of the different amino acid-based surfactants prepared with arginine, phenylalanine, and tryptophan.

**Figure 2:**
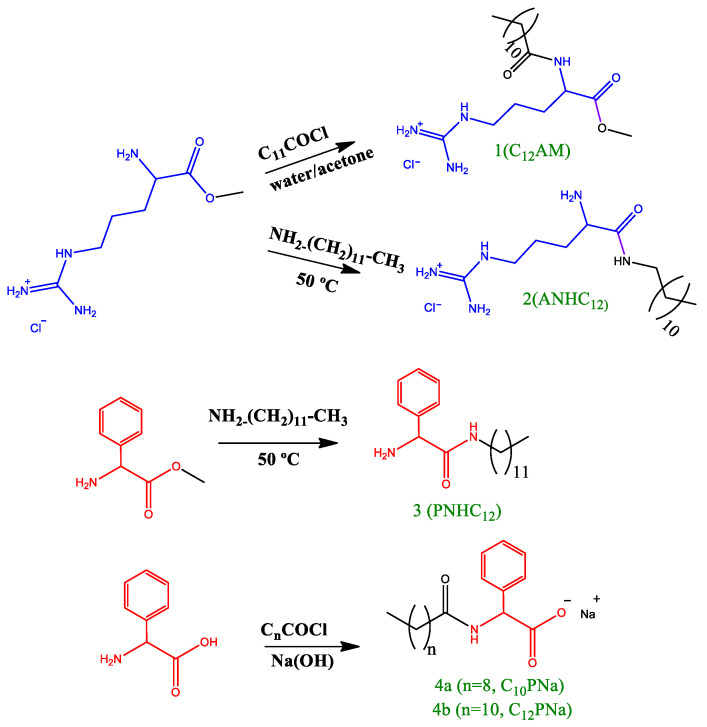
Synthetic pathway to obtain monocatenary surfactants with one amino acid on the polar head.

**Figure 3:**
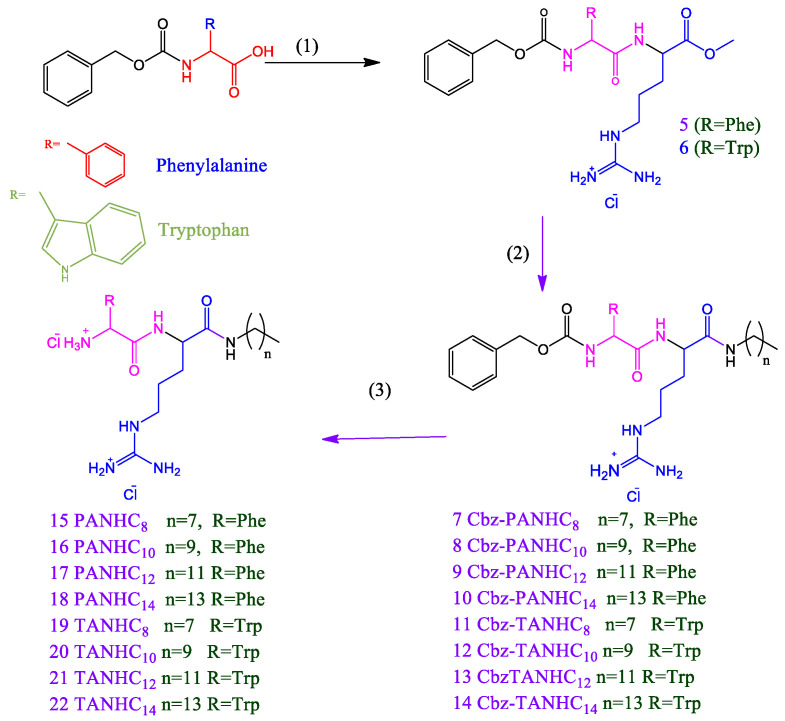
Synthetic pathway to obtain monocatenary surfactants with two amino acids of the N-alkylmide type. (1) ArgOMe-2HCl/BOP/Dabco in dichloromethane at room temperature (2) NH_2_-(CH_2_)_n_-CH_3_/50 °C (3) Pd/C in methanol/HCl at room temperature.

**Figure 4:**
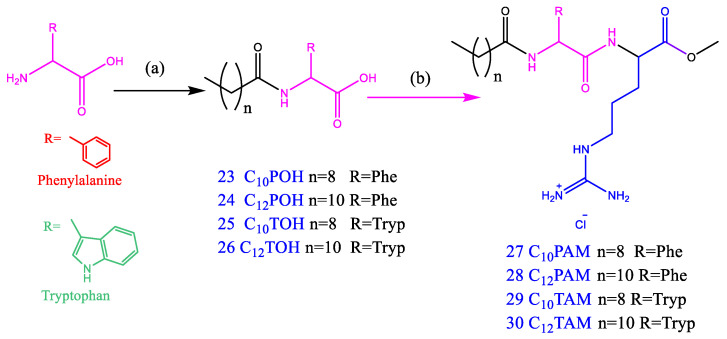
Synthetic pathway for the synthesis of monocatenary surfactants with two amino acids of the N^α^-Acyl-aa type: (**a**) acyl chloride, water/acetone, (**b**) ArgOMe-2HCl, BOP, DABCO, CH_2_Cl, room temperature.

**Figure 5:**
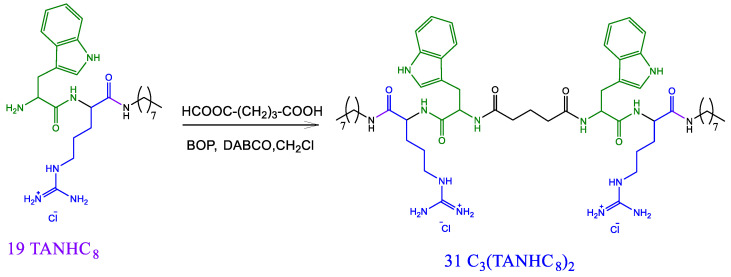
Synthetic pathway to obtain the gemini C_3_(TANHC_8_)_2_ surfactant.

Here, we report the synthesis and characterization of a library of amino acid-based surfactants designed to be used as antimicrobials. The head group architecture (number and type of amino acids, density of cationic charge, ionic character) and hydrophobic moiety (alkyl chain length and position of the hydrophobic group) were systematically changed to modify the surfactant biological and aggregation behavior (Figure 1). The chemical structure of these new compounds and their corresponding acronyms are shown in Figure 1, Figure 2, Figure 3, Figure 4 and Figure 5. The prepared surfactants are described below.

(1) Four monocatenary surfactants with one amino acid on the polar head and one C_12_ alkyl chain (Figure 1): N^α^-dodecyl-phenylalanine sodium salt (C_12_PNa), an anionic surfactant with a C_12_ alkyl chain and negative charge on the carboxylic group of phenylalanine; N^α^-dodecyl-arginine methyl ester hydrochloride (C_12_AM), with a C_12_ alkyl chain linked to the α-amine group of arginine and a cationic charge on the protonated guanidine group; arginine dodecyl amide dihydrochloride (ANHC_12_), with two cationic charges on arginine (one on the protonated guanidine group and the other on the protonated amine group) and the alkyl chain linked by an amide bond to the carboxylic group of the amino acid; and phenylalanine dodecyl amide hydrochloride (PNHC_12_), in which the cationic charge lies on the protonated amine group of phenylalanine and the alkyl chain is linked to the carboxylic group of the amino acid.
(2) Eight monocatenary surfactants with two amino acids on the polar head (Phe/Arg [PANHC_n_] or Trp/Arg [TANHC_n_]), two cationic charges (one on the protonated guanidine group and the other on the protonated amine group of the aromatic amino acid) and the alkyl chain (C_8_-C_14_) linked to the carboxylic group of the amino acid by an amide bond (Figure 1): phenylalanine-arginine octyl amide dihydrochloride (PANHC_8_), phenylalanine-arginine decyl amide dihydrochloride (PANHC_10_), phenylalanine-arginine dodecyl amide dihydrochloride (PANHC_12_), phenylalanine-arginine tetradecylamide dihydrochloride (PANHC_14_), tryptophan-arginine octyl amide dihydrochloride (TANHC_8_), tryptophan-arginine decyl amide dihydrochloride (TANHC_10_), tryptophan-arginine dodecyl amide dihydrochloride (TANHC_12_) and tryptophan-arginine tetradecylamide dihydrochloride (TANHC_14_).
(3) Four monocatenary surfactants with two amino acids on the polar head (Phe/Arg [CnPAM] or Trp/Arg [CnTAM]), one cationic charge lying on the protonated guanidine group of arginine and the alkyl chain (C_10_ or C_12_) linked to the α-amino group of the aromatic amino acid by an amide bond (Figure 1): N^α^-dodecyl-phenylalanine-arginine methyl ester hydrochloride (C_12_PAM), N^α^-decyl-phenylalanine-arginine methyl ester hydrochloride (C_10_PAM), N^α^-dodecyl-tryptophan-arginine methyl ester hydrochloride (C_12_TAM) and N^α^-decyl-tryptophan-arginine methyl ester hydrochloride (C_10_TAM).
(4) One gemini surfactant with two C_8_ alkyl chains, two head groups with Arg-Tryp amino acids and a spacer chain of three methylene groups (C_3_(TANHC_8_)_2_) (Figure 1).

Conductivity and fluorescence were measured to determine the critical micellar concentration (cmc) of all the prepared surfactants. Antimicrobial activity was tested against representative Gram-positive and Gram-negative bacteria. Additionally, the biodegradability of the synthesized amino acid-based surfactants was determined. It is expected that the modification of the head group architecture and the hydrophobic moiety will allow us to modulate aggregation behavior and biological activity. The main aim of this study is to shed light on the effect of different structural features of amino acid-based surfactants on their antibacterial activity and biodegradation profile.

## 2. Materials and Methods

### 2.1. Materials

Organic solvents were reagent grade and used without further purification. Acyl chlorides and fatty amines were from Fluka. N-benzyloxycarbonyl-L-phenylalanine (N-Cbz-L-Phe) N-benzyloxycarbonyl-L-tryptophan (N-Cbz-L-Try), L-phenylalanine, L-tryptophan, arginine methyl ester dihydrochloride, L-phenylalanine methyl ester hydrochloride and L-tryptophan methyl ester hydrochloride were obtained from TCI Europe. 1,4 diazabiciclo[2.2.2]octane (DABCO) was from Sigma. Benzotriazol-1-yloxy) tris(dimethylamino) phosphonium hexafluorophosphate (BOP) was from Sigma-Aldrich. Trifluoroacetic acid (TFA) and palladium on activated charcoal (Pd/C, 10%) were supplied by Merck (Darmstadt, Germany). Deuterated solvents were purchased from Eurotop. Mueller-Hinton Broth was purchased from Oxoid Ltd., Basingstoke, UK. The 96-well polypropylene microtiter plates were from Costar; Corning Incorporated, Corning, NY, USA). Water from a Milli-Q Millipore system was used to prepare aqueous solutions.

### 2.2. Synthesis

#### 2.2.1. Preparation of Dodecyl Arginine Methyl Ester Hydrochloride C_12_AM (**1**, Figure 2)

Arginine methyl ester (0.010 mol) was dissolved in a mixture of acetone/water 40/60 (30 mL). A solution of aqueous Na(OH) was added until pH = 10, and the mixture was cooled to 0 °C. After the completion of the reaction, HCl was added, and a white solid precipitated. Pure **1** compound was then obtained after several crystallizations in hot acetonitrile.

#### 2.2.2. Preparation of Arginine Dodecyl Amide Dihydrochloride ANHC_12_ (**2**, Figure 2) and Phenylalanine Dodecyl Amide Hydrochloride PNHC_12_ (**3**, Figure 2)

Arginine methyl ester or phenylalanine methyl ester (0.003 mol) was weighed in a round flask. Then, 0.014 moles of dodecyl amine were added. The mixture was heated using a glycerol bath to the melting point of the amine and was stirred at this temperature for three hours. After the completion of the reaction, the mixture was cooled at room temperature, and several crystallizations in methanol/acetonitrile were carried out to remove the excess of fatty amine in order to obtain the pure **2** and **3** surfactants.

#### 2.2.3. Preparation of N-Acyl Phenylalanine Sodium Salt (**4a**, **4b**, Figure 2)

A solution of 100 mL of acetone/water (30/70) containing 0.020 moles of L-phenylalanine was placed in a round-bottom flask. A solution of aqueous Na(OH) was added until pH = 10 and the mixture was cooled to 0 °C. The corresponding fatty acid chloride (0.012 mols) was added dropwise and the mixture was stirred at room temperature for 4 h. HCl was then added and a white solid precipitated. The resulting solid was washed with water and dried over vacuum. Pure Nα-acyl phenylalanine compounds were obtained by several crystallizations from hot acetonitrile. After that, 0.005 mol of pure N-acyl phenylalanine dissolved in 15 mL of methanol were neutralized with 0.005 mol of Na(OH) and pure **4a** and **4b** surfactants were obtained by removing the solvent.

#### 2.2.4. Preparation of Phenylalanine Arginine Alkyl Amide Dihydrochloride (PANHC_n_, **15**–**18**, Figure 3) and Tryptophan-Arginine Alkyl Amide Dihydrochloride (TANHC_n_, **19**–**22**, Figure 3) Surfactants

These compounds were prepared using a three steps procedure:

(a) Synthesis of N^α^-carbobenzoxy-phenylalanine-arginine methyl ester hydrochloride (**5**, Figure 3) and Nα-carbobenzoxy-tryptophan-arginine methyl ester hydrochloride (**6**, Figure 3). A solution of N^α^-carbobenzoxy-phenylalanine or N^α^-carbobenzoxy-tryptophan (0.025 mol) in 50 mL of dichlorometane (CH_2_Cl_2_) was treated with 0.060 mol of 1,4-diazabicyclo[2.2.2]octane (DABCO). Then, 0.025 mol of arginine methyl ester dihydrochloride was added until the total solubilization of the starting reagents. Finally, 0.025 mol of benzotriazol-1-yloxytris(dimethylamino)phosphonium hexafluorophosphate (BOP) was added, and the mixture was stirred for 4 h. After that, the mixture reaction was cooled at 0 °C, and the solid precipitated was isolated by filtration. The solid was washed with diethyl ether and water, and pure **5** and **6** compounds were obtained by crystallizations in acetonitrile/methanol.
(b) Synthesis of N^α^-carbobenzoxy-phenylalanine-arginine alkyl amide hydrochloride (**7**–**10**, Figure 3) and N^α^-carbobenzoxy-tryptophan-arginine alkyl amide hydrochloride (**11**–**14**, Figure 3). Compound **5** or **6** (0.010 mol) was weighed in a round flask. Then, 0.100 moles of the corresponding alkyl amine were added. The mixture was heated using a glycerol bath to the melting point of the amine and was stirred at this temperature for three hours. After the completion of the reaction, the mixture was cooled at room temperature, and several crystallizations in methanol/acetonitrile were carried out to remove the excess of fatty amine. Finally, pure **714** surfactants were obtained by preparative HPLC.
(c) Synthesis of phenylalanine-arginine alkyl amide dihydrochloride (**15**–**18**) and tryptophan-arginine alkyl amide dihydrochloride (**19**–**22**). Compounds **15**–**22** were obtained by hydrogenation of the corresponding pure **7**–**14** (0.007mol) in 50 mL of MeOH/HCl using Pd in activated charcoal (10% Pd) as catalyst. The reaction was carried out at room temperature and atmospheric pressure. After completion of the reaction, the catalyst was filtered off on Celite, and the methanol was removed at vacuum. Pure **15**–**22** compounds were obtained by crystallizations from Me(OH)/ACN.

#### 2.2.5. Preparation of N^α^-Acyl Phenylalanine-Arginine Methyl Ester Hydrochloride (C_n_PAM, **27**–**28**, Figure 4) and N^α^-Acyl Tryptophan-Arginine Methyl ester Hydrochloride (CnTAM, **29**–**30**, Figure 4)

These compounds were prepared using a three steps procedure:

(a) Synthesis of N^α^-Acyl phenylalanine (**23**, **24**, Figure 4) and N^α^-Acyl tryptophan (**25**, **26**, Figure 4). A solution of 40 mL of acetone/water (30/70) containing 0.015 moles of L-phenylalanine or L-tryptophan was placed in a round-bottom flask. A solution of aqueous Na(OH) was added until pH = 10, and the mixture was cooled to 0 °C. Dodecyl or decyl chloride (0.016 mols) was added dropwise, and the mixture was stirred at room temperature for 4 h. HCl was then added and a white solid precipitated. The resulting solid was washed with water and dried over vacuum. Pure compounds were obtained by several crystallizations from hot acetonitrile.
(b) Synthesis of N^α^-Acyl phenylalanine methyl ester hydrochloride (**27**, **28**) and N^α^-Acyl tryptophan (**29**, **30**). A solution of **23**, **24** or **25**, **26** (0.012 mols) in 50 mL of Dimethylformamide was treated with 30 mmol of DABCO. Then, 0.012 mols of arginine methyl ester dihydrochloride were added under stirring until the compounds were completely solubilized. After that, 0.012 mols of BOP were added and the mixture was agitated for 3 h. The DMF was removed over vacuum and the mixture was washed with diethyl ether. Pure **27**–**30** surfactants were obtained by preparative HPLC.

#### 2.2.6. Preparation of the Gemini Tryptophan Arginine Octyl Amide Derivative C_3_(TANHC_8_)_2_ (**31**, Figure 5)

A solution of TANHC_8_ (0.005 mols) in 30 mL of CH_2_Cl was treated with 0.010 mols of DABCO. Then, 0.0025 mols of glutaric acid were added under stirring until the compounds were completely solubilized. After that, 0.025 mols of BOP were added, and the mixture was agitated for 3 h. The CH_2_Cl was removed over vacuum, and the mixture was washed with diethyl ether. Pure **30** surfactant was obtained by preparative HPLC.

The identification of all synthesized compounds was carried out by HPLC, elemental analysis and NMR experiments. Detailed characterization of the products is included in the Supplementary Materials.

### 2.3. HPLC, NMR and HRMS Analysis

The reaction progress as well as the purity of the new surfactants were measured by HPLC (Merck-Hitachi D-2500, Gerber Hausen, Germany) using a LiChrosphere 100 CN column 5 μm, 250 × 4 mm. The solvent system used was: aqueous phase, trifluoroacetic acid 0.1% (*v*/*v*) in H_2_O and organic phase, trifluoroacetic acid 0.095% (*v*/*v*) in H_2_O/ACN (1:4). UV-detector at λ = 215 nm and flow rate of 1 mL/min.

The NMR spectra were recorded on a Varian spectrometer at 499.803 (^1^H) and 125.233 (^13^C) MHz, respectively. Sample solutions were prepared in deuterated methanol. The HRMS identification of compounds was performed on an Acquity UPLC System and a LTC PremierTM XE Benchtop orthogonal acceleration time-of-flight (Water Corporation) with an electrospray ionization source. All data were processed and displayed using MassLynx software.

### 2.4. Preparative HPLC

This methodology was used to purify the target surfactants. HPLC was performed using a Waters preparative HPLC system equipped with a Kromasil 100 C_8_ 5 μm 250 × 2.12 column. Crude surfactant (500 mg dissolved in 5 mL of methanol) was loaded onto the preparative column. The solvent system consisted of 0.1% (*v*/*v*) TFA in water (solvent A) and 0.1% (*v*/*v*) TFA in acetonitrile (solvent B). A flow rate of 20 mL/min with a linear gradient from 40 to 100% of phase B in 20 min was used. The absorbance of the eluate was measured at 215 nm. All collected fractions were analyzed by analytical HPLC, and pure portions were dried under vacuum.

### 2.5. pKa Determinations

The pKa values of these amino acid-based surfactants were determined by titration 2 mL of a postmicellar aqueous surfactant solution with an aqueous Na(OH) solution, at 25 °C, with a pH electrode (l 8102 ROSS Thermo Orion). The experiments were performed under nitrogen gas atmosphere and magnetic stirring. The pKa was determined based on the pH corresponding to semi equivalent point of the neutralization curve.

### 2.6. Fluorescence Measurements

A Shidmadzu RF 540 spectrofluorometer (Shimadzu, Kioto, Japan) was used to measure cmc by fluorescence, using pyrene (Sigma-Aldrich, San Luis, MO, USA) as a fluorescence probe, at 25 °C. The fluorescence emission spectra of pyrene dissolved in surfactant aqueous solutions were recorded from 340 to 450 nm after excitation at 332 nm. The ratio of the first to the third vibrionic peaks of the fluorescence spectra (I_I_/I_III_) in this region depends on the environment polarity and can be used to calculate the cmc.

### 2.7. Conductivity

The conductivity of aqueous surfactant solutions was measured with an Orion Cond Cell 011010A (Thermo scientific, Waltham, MA, USA) with platinized platinum electrodes in conjunction with a Thermo Orion 550A with a cell constant of 0.998 cm^−1^. The cell constant was calibrated with NaCl/KCl solutions. Measurements of samples prepared in Millipore water were carried out at increasing concentrations to minimize errors from possible electrode contamination. The conductivity of pure water was substrated from the conductivity of each sample.

### 2.8. Antimicrobial Activity

#### 2.8.1. Antimicrobial Susceptibility Test

The minimal inhibitory concentration (MIC) of the new surfactants was determined using a broth microdilution assay [19]. Serial dilutions of every compound, between 256 and 2 μg/mL, in MH Broth were dispensed (200 μL) in the corresponding wells of a 96-well polypropylene microtiter plate. The nutrient broth starter culture of each bacterial strain (10 μL) was added to achieve final inoculum of ca. 5 × 10^−5^ colony-forming units (CFU) per mL. Nutrient broth medium without the compound served as growth control. The development of turbidity in an inoculated medium is a function of growth, and reflects an increase in both mass and cell number. The MIC was defined as the lowest concentration of antibacterial agent that inhibited development of visible growth after 24 h of incubation, at 37 °C. To confirm this observation, 20 μL of resazurin at 0.015% *w*/*v* was added to each well and left to react for approximately 1 h for bacteria, at 35°. After the incubation period, the indicative of bacterial growth, i.e., changing from blue to pink, confirmed the MIC value. To obtain the MBC, the antimicrobial concentration corresponding to at least 3-log reductions in viable cells, an aliquot of 10 μL of the MIC well and the 2 concentrations immediately above were seeded over agar MH and incubated for 24 h, at 35 °C. The MBC were determined as the lowest concentration in which no colonies were observed on the agar plates.

#### 2.8.2. Exposure of Cationic Surfactants to Microorganisms

Suspensions of microorganisms were obtained from an overnight culture of *S. aureus* ATCC 6538 and *P. aeruginosa* ATCC 9027, at 30 °C. Four colonies were suspended in 10 mL of peptone buffered solution (pH 7.0). Each bacterial suspension was pelleted by centrifugation at 8000× *g* for 20 min, washed in sterile filtered peptone buffered solution (pH 7.0), centrifuged again, and finally suspended to obtain a concentration of 10^7^–10^8^ CFU/mL. An appropriate volume (250 μL) of the respective cell suspensions was used to inoculate flasks containing 24 mL of buffered peptone water (pH 7) to obtain a cell density of ca 10^5^–10^6^ CFU/mL. To flasks containing 24 mL of the respective bacterial suspensions, surfactant stock solution (1 mL) was added to reach a final concentration of surfactant corresponding to the MIC. The inoculated flasks were kept in darkness, at room temperature. The contact time established for flow cytometry (FC) was 30 min. Then, suspensions were centrifuged at 8000× *g* for 30 min, and the sediment resuspended in 2 mL of filtered peptone buffered water (pH 7.0). In parallel control experiments, cells were incubated in surfactant-free buffer solution and treated under the same conditions.

#### 2.8.3. Bacterial Counts

After contact with C_12_PAM, reduction in bacterial viability was calculated by viable cell counts, according to the colony forming units (CFU mL^−1^) obtained on TSA. After appropriate dilution in Ringer’s solution, 0.1 mL was inoculated on plates and incubated at 30 °C for 8–24 h. The cell count was performed in triplicate, and the mean was determined. Based on these data, growth inhibition was calculated according to the reduction in viability as follows [20]:

(1 − N_T_/N_C_) × 100

where N_T_ is the bacterial count in the treated sample and N_c_ is the bacterial count in the control sample.

#### 2.8.4. Flow Cytometry

The staining protocols for FC experiments were as follows: 10 μL of a 1 mg mL^−1^ stock solution of propidium iodide (PI) in distilled water was added to 1 mL of the bacterial suspension containing 10^7^–10^8^ CFU/mL (prepared as described above) in filtered buffered peptone water. Staining was carried out at room temperature before the FC analysis. To evaluate membrane potential, 2 μL of a 250 mmol/L stock solution of bis-oxonol in ethanol was added to 1 mL of the bacterial suspension. Heat-killed cells (30 min at 70 °C) were used as a positive control for PI and bis-oxonol staining protocols. FC experiments were performed in duplicate using a Cytomics FC500 MPL flow cytometer (Beckman Coulter, Inc., Fullerton, CA, USA). Samples were excited using a 488 nm air-cooled argon-ion laser at 15 mW power. The instrument was set up with the standard configuration: green (525 nm) fluorescence for bis-oxonol, red (675 nm) for PI. The results were collected on logarithmic scales. Optical alignment was checked using 10 nm fluorescent beads (Flow-Check fluorospheres, Beckman Coulter). The cell population was selected by gating in a FS vs. SS dot plot, excluding aggregates and cell debris. Fluorescence histograms were represented in single-parameter histograms (1024 channels). The windows used to calculate percentages were set using living and dead cell populations for each strain. Data were analyzed using Summit^®^ ver. 3.1 software (Cytomation, Fort Collins, CO, USA).

### 2.9. Aerobic Biodegradability

The aerobic biodegradability of amino acid-based surfactants was evaluated using the OECD 310 method (CO_2_ headspace test). This standard method evaluates the mineralization of an organic compound in an aqueous medium by measuring the production of CO_2_. Therefore, the rate of biodegradation of amino acid-based surfactants was determined by measuring over time the CO_2_ generated from the degradation of these molecules by microorganisms. Each surfactant, as the sole source of carbon and energy, was added at 15 mg C/L to a mineral buffered aqueous medium (pH 7.4 ± 0.2) inoculated with a mixed population of microorganisms obtained from activated sludge from a waste water treatment plant (Manresa, Barcelona). Sodium benzoate (NaBz) at 20 mg C/L was used as reference substance. Biodegradation tests ran for 28 days. For each test surfactant, inhibition tests with binary mixtures of NaBz and amino acid-based surfactant at 20 and 15 mg C/L, respectively, were also conducted. Biodegradation was determined by measuring the net increase in inorganic carbon over time, i.e., the excess of CO_2_ generated in the vessels containing surfactant relative to blank vessels that measured the CO_2_ endogenous production, using a carbon analyzer (Shimadzu TOC-5050). Biodegradation extent was determined as a percentage of the theoretical maximum production of inorganic carbon based on the initial concentration of surfactant. The pass level of the method is set at 60%, and therefore, a compound that undergoes a higher biodegradation percentage can be considered readily biodegradable.

## 3. Results and Discussion

### 3.1. Synthesis

The self-assembling and biological properties of surfactants depend on the structural features of their hydrophobic and hydrophilic moieties. In this work, we prepared amino acid-based surfactants in which the two moieties were systematically modified to study the effects of structural parameters on the surfactant physicochemical properties, antimicrobial activity and biodegradation. Four types of amino acid-based surfactants were prepared based on arginine, phenylalanine and tryptophan (Figure 2, Figure 3, Figure 4 and Figure 5). The effect of the hydrophilic group was determined by preparing surfactants with one of two amino acids on the polar head and one or two cationic charges. Variable alkyl chain lengths (C_8_–C_14_ derivatives) and positioning of the hydrophobic group was also tested (compounds with the alkyl chain linked to the α-amino group of the amino acid, and others in which the hydrophobic part is linked to the carboxylic group of the amino acid). Finally, a gemini surfactant was prepared consisting of two monocatenary tryptophan-arginine-octyl amide compounds connected at the head group by a spacer chain of three methylene groups. All these new surfactants were prepared from natural fatty acids, fatty amines, and amino acids, the amide bonds ensuring stability and improving biodegradability.

#### 3.1.1. Monocatenary Surfactants with a Single Amino Acid on the Polar Head (Figure 2)

The monocatenary C_12_AM (**1**) was prepared in a one-step procedure using commercially available arginine methyl ester. The acylation of the free amino group of Arg-OMe-2HCl with dodecyl chloride was carried out in water/acetone (70/30) at pH = 9.0. No protecting group was required, as at this pH, the guanidine group of the arginine remains protonated and cannot react with the dodecyl chloride. Several crystallizations in hot acetonitrile gave pure C_12_AM. The monocatenary ANHC_12_ (**2**) and PNHC_12_ (**3**) were prepared simply by heating the starting Arg-OMe-2HCl or Phe-OMe-HCl with dodecyl amine without any solvent. The anionic derivatives CnPNa (**4a**, **4b**) were prepared by acylation of the free amino group of phenylalanine in acetone/water (30/70) at pH = 9.0. The four procedures were monitored by HPLC, and after four hours, more than 92% of the starting materials had been converted. Large-scale synthesis was achieved in a short time and without the use of any activation agent. The nuclear magnetic resonance (NMR) spectra and mass spectra are consistent with the target compounds (see Supplementary Materials).

#### 3.1.2. Monocatenary Surfactants Based on Phenylalanine-Arginine Alkyl Amide Dihydrochloride (PANHC_n_) and Tryptophan-Arginine Alkyl Amide Dihydrochloride (TANHC_n_) (Figure 3)

The eight compounds in the second group of surfactants, four arginine-phenylalanine derivatives (PANHCn) and four tryptophan-arginine derivatives (TANHCn), have two amino acids in the polar head bound by peptide linkages. All have two positive charges on the polar head and an alkyl chain of C_8_–C_14_, and the hydrophobic chain is also linked to the carboxylic group of arginine by a peptide linkage.

The three-step synthetic procedure for the preparation of the O-alkyl amide derivatives is outlined in Figure 3. Compounds **5** and **6** were obtained using the BOB reagent to condense the α-carboxylic group of Cbz-phenylalanine/tryptophan methyl ester with the amino group of Arg-OMe-2HCl. These reactions occurred at room temperature in CH_2_Cl_2_, and the conversion of starting material was over 90% after one hour. The formation of amide bonds between amino acids usually requires the use of activating agents. Intermediates **7**–**14** were obtained using the procedure for the preparation of ANHC_12_ and PNHC_12_ (Figure 2); compounds **5** and **6** were mixed with the corresponding fatty amine (C_8_–C_14_) at the amine melting point. The reactions were also completed in 2–3 h. Finally, removal of the Cbz protecting group by means of catalytic hydrogenation in methanol using Pd/C as the catalyst allowed us to obtain the target cationic surfactants **15**–**22**. The reaction was carried out at room temperature and atmospheric pressure. Hydrochloric acid was added to obtain the surfactants as dihydrochloride salts. In this approach, organic solvents were used in the first step because they enabled the synthesis to be carried out in a short time, with typical overall yields of 70–75%. The other two steps were performed without using dangerous organic solvents; moreover, the reactions were achieved at room temperature and atmospheric pressure.

#### 3.1.3. Monocatenary Surfactants Based on N^α^-Acyl-Phenylalanine-Arginine-Methyl Ester Hydrochloride (CnPAM) and N-^α^-Acyl-Trypthophan-Arginine-Methyl Ester (CnTAM) (Figure 4)

A two-step procedure was used to prepare these derivatives. In the first step, N^α^-acyl-tryptophan (**25**, **26**) and N^α^-acyl-phenylalanine (**23**, **24**) were obtained by acylation of the α-amino group of commercial tryptophan and phenylalanine with the corresponding acyl chloride. The reaction was monitored by HPLC, and at 6 h, 90% of the starting amino acid had been converted. The compounds were purified by several crystallizations in methanol/acetonitrile. The second step involved the condensation of the free carboxylic group of **23**–**26** with the α-amino group of the arginine methyl ester. The use of the coupling agent BOP in the presence of the base DABCO to activate the carboxylic groups rapidly afforded the target compounds **27**–**30**. The ^1^HNMR and ^13^CNMR spectra agreed with the target structure, and the mass spectra showed the positive molecular ion (Supplementary Materials). The procedure used to prepare these surfactants was easy and efficient, and by avoiding the use of protected amino acids, it allowed us to carry out a rapid synthesis on a large scale and at a reasonable cost.

#### 3.1.4. Gemini Surfactant Based on Tryptophan-Arginine Octyl Amide (Figure 5)

This gemini derivative was prepared using compound **19** (TANHC_8_) as the starting material via the synthetic pathway depicted in Figure 5. Two TANHC_8_ molecules were condensed to both carboxylic groups of the glutaric acid, using BOP as the condensing agent and DABCO as the organic base. In a previous study, our group successfully used this method to prepare gemini surfactants from arginine, lysine, and histidine [21]. The target gemini derivative was purified using preparative HPLC.

#### 3.1.5. Determination of pKa

The pKa values of these amino acid-based surfactants were determined by titration of a post-micellar surfactant solution (2 mL) of the corresponding surfactant (Figure 2) with aqueous Na(OH), at 25 °C. The apparent pKa was based on the pH corresponding to the semi-equivalent point of the neutralization curve.

**Figure 6:**
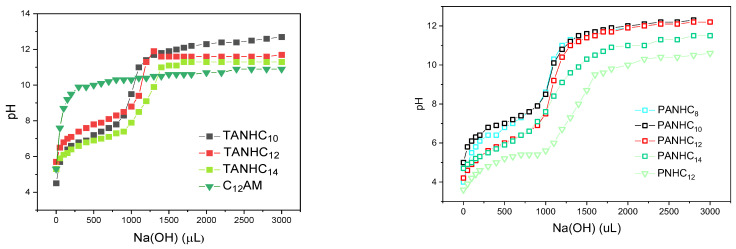
Tritation curves of the C_12_AM, PANHC_n_ and TANHC_n_ surfactants.

The cationic charge of the prepared pH-sensitive surfactants lies on the protonated amine or guanidine group of the amino acids, and its density depends on the apparent pKa corresponding to the acid-base equilibrium of the protonated groups. Given that the biological properties of cationic surfactants are governed by the cationic charge density [13], it is essential to know the number of cationic charges as well as the pKa values at the pH at which antimicrobial activity is determined. Figure 6 shows the pH values plotted as a function of the volume of NaOH added. According to the equilibria, the pKa corresponds to the pH value obtained at the semi-equivalent titration point (Table 1).

In the monocatenary C_12_AM, diamino acid-based surfactants of the N^α^-acyl type (C_12_PAM, C_10_PAM, C_12_TAM, C_10_TAM) and the gemini (C_3_(TANHC_8_)_2_), the cationic charge lies on the arginine guanidine group. The strong basic character of this guanidine group makes it difficult to obtain the pKa values from the pH/Na(OH) curves (see the titration curve obtained for C_12_AM in Figure 6). However, considering the results obtained for C_12_AM and the data reported for other amphiphiles containing arginine [22] the pKa can be expected to be similar to that of C_12_AM (around 9–10). It is therefore likely that the guanidine group in these surfactants remained fully protonated in all the biological experiments carried out in this work. Considering that the pKa of the guanidine group of the arginine amino acid is 12.5, it underwent a significant decrease, perhaps induced by aggregation [22]. The monocatenary PNHC_12_ has one cationic charge on the chlorohydrate amino group of phenylalanine, and its pKa is 5.4; thus, aqueous solutions of this surfactant with pH values of 4.4–6.4 contain protonated and non-protonated species, whereas solutions with pH values higher than 7 contain non-protonated molecules.

The diamino acid-based compounds of the O-alkyl amide type (PANHCn and TANHCn) contain two cationic charges: the protonated chlorohydrate α-amino group of the aromatic amino acid and the chlorohydrate guanidine group of arginine. The pKa corresponding to the protonated guanidine group is expected to be around 9–10, indicating that this group maintained a cationic charge in all the experiments. The pKa of the protonated amino group of PANHCn decreased as the alkyl chain increased, ranging from 6.8 for PANHC_8_ to 6.0 for the C_14_ derivative. This tendency was not observed in TANHCn, in which the pKa values varied randomly from 7 to 8 regardless of the alkyl chain length.

The pKa values obtained for the α-amino group of PANHCn and TANHCn indicate weak acidic properties, and their cationic nature therefore depends on the pH of the solubilizing media. At pH 7, these surfactants had a percentage of molecules with two positive charges, the rest having only one positive charge. Similar pKa values were obtained for the α-amino group of glycerolipid arginine-based surfactants [23].

#### 3.1.6. Micellization

The aggregation properties of these amino acid-based surfactants in aqueous media were determined by fluorescence and conductivity measurements. The steady-state fluorescence was measured using pyrene as the solvatochromic probe.

**Figure 7:**
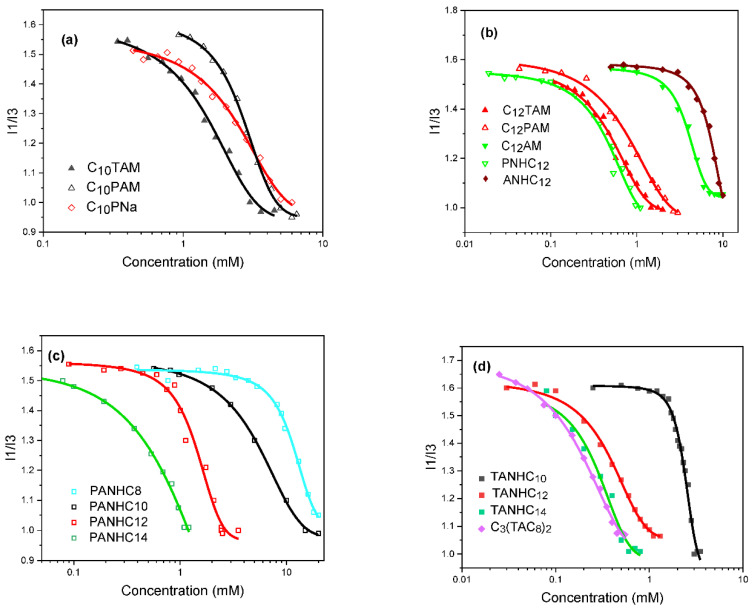
Variation of the I1/I3 ratio as a function of the surfactant concentration in aqueous solution, at 25 °C: the effects of (**a**) the charge, (**b**) polar head, (**c**) alkyl chain and (**d**) both the alkyl chain and dimerization of the monocatenary surfactants.

Figure 7 shows the I1/I3 ratio of pyrene as a function of the surfactant concentration. The abrupt sigmoidal decrease in the curves indicates that the pyrene migrated to a more hydrophobic environment and that molecular aggregates were formed in the aqueous surfactant solutions. The cmc values were taken from the intersection of the stable line corresponding to the low surfactant concentrations and the line corresponding to the abrupt change in the I1/I3 ratio (Table 2).

The cmc values of surfactants with 12-carbon alkyl chains, C_12_AM, ANHC_12_, PNHC_12_, C_12_PAM, C_12_TAM, PANHC_12_ and TANHC_12_, were 4, 7.2, 0.9, 1.1, 0.6, 1.6 and 0.6 mM, respectively. C_12_AM and ANHC_12_ share a common feature in the polar head: an arginine amino acid with a protonated guanidine group. However, as ANHC_12_ contains an extra cationic charge (the protonated amino group of arginine), its polarity and cmc are higher compared to C_12_AM. The cmc of these two derivatives is similar to those reported for monocatenary surfactants based on lysine and histidine amino acids [24] and for alkyl dimethyl ammonium bromides bearing a side chain with two additional CH_2_ groups [25].

Compared with C_12_AM/ANHC_12_, the cmc values of C_12_PAM, C_12_TAM, PANHC_12_ and TANHC_12_ were much lower. The incorporation of an aromatic amino acid with a hydrophobic character significantly reduces the polarity of the resulting surfactants, which has a pronounced effect on their micellization process. The higher hydrophobicity of these diamino acid surfactants enhances their tendency to form micelles in water. Quantitatively, the cmc of C_12_PAM was similar to that of the N^α^-acyl arginine methyl ester derivative bearing an alkyl chain with two additional CH_2_ groups, and the cmc of C_12_TAM was comparable to that of the N^α^-dodecyl arginine methyl ester derivative with four extra CH_2_ groups. According to the literature, the influence of structural changes in the polar head on cmc values depends on the polarity of the added moieties. We observed that PANHC_12_ and PNHC_12_ shared similar cmc values. Cholinium, imidazolinium and pyridinium ILs with phenylalanine on the polar head had much lower cmc values than their IL homologs without the aromatic amino acid [26]. The cmc reported for a series of cationic surfactants, ester hydrochlorides of phenylalanine, were lower than those of PANHCn and PNHC_12_ [27]. It has also been observed that the incorporation of a polar amino acid such as lysine on the molecular structure of C_12_AM increases its polarity and cmc [24]. Structural changes in the head group of surfactants with neutral moieties do not significantly affect the cmc [28].

Due to the presence of two aromatic rings on the side chain of tryptophan, C_12_TAM and TANHC_12_ showed lower cmc values than the phenylalanine derivatives C_12_PAM and PANHC_12_. On comparing the N^α^-derivatives C_12_PAM and C_12_TAM with the O-alkyl amide derivatives PANHC_12_ and TANHC_12_, it can be observed that very similar cmc values were obtained for surfactants with the same amino acid, which indicates a similar polarity of the polar head. Neither the second cationic charge nor the position of the alkyl chain affected the cmc of these surfactants. The micellization process seems to be governed by the alkyl chain length, the hydrophobicity of the aromatic amino acid, and the cationic charge of the guanidine group. The scarce effect of the second cationic charge may be due to the low pKa of the protonated amine group. It was also observed that the ionic character of the polar head did not affect the cmc. Notably, the cmc of the anionic surfactant C_10_PNa was similar to that of the cationic C_10_PAM with the same alkyl chain length. These results agree with those reported by Bahri et al. for SDS and DTAB [29].

As expected, cmc values also depended on the alkyl chain length. In homologs with the same polar head (PANHCn and TANHCn), the cmc decreased as the number of carbons in the hydrophobic chain increased. This behavior, common in all types of surfactants, is ascribed to the more hydrophobic character of molecules with longer alkyl chains [25]. The increase in hydrophobicity reduces water solubility, and the surfactants are forced to form molecular aggregates in solution. In an aqueous medium, the cmc usually decreases as the CH_2_ groups increase, until about 16 [25].

**Figure 8:**
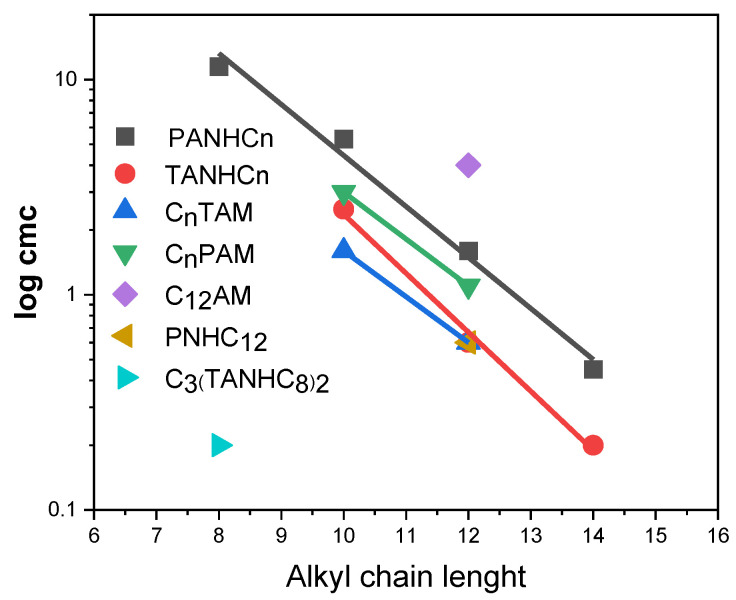
Effect of the alkyl chain length on the cmc of the PANHCn, TANHCn, CnPAM, CnTAM, PNHC12, C_12_AM, and gemini surfactants. (Series PANHCn log cmc = 3.019–0.23n, TANHC_n_ log cmc = 3.11–0.27n, C_n_TAM log cmc = 2.33–0.21n, and C_n_PAM log cmc = 2.65–0.21n).

In surfactant homologs, the cmc decreases logarithmically as the number of carbons in the alkyl chain increases, following the empirical Klevens equation [30]: log cmc = A − Bn, where A and B are constant parameters for a particular homologous series. Parameter A is related to the contribution of the polar head to micelle formation, whereas parameter B reflects the average contribution to the micelle formation of each additional CH_2_ group in the alkyl chain. The C_n_PAM, C_n_TAM, PANHC_n_ and TANHC_n_ series showed nearly parallel plots with similar slope values (0.21–0.27, Figure 8). This means that the incorporation of a CH_2_ group in the alkyl chain approximately halves the cmc values. The B value obtained for these surfactants is similar to that reported for cationic surfactants with different polar heads bearing phenylalanine [26,27] histidine [31], pyridinium, imidazolinium [32], and cholinium head groups [26].

Considering its C_8_ alkyl chain, the cmc value of the gemini C_3_(TANHC_8_) was very low, being similar to the cmc of the C_14_ derivative monomeric counterparts. In general, all types of gemini surfactants [33], including those based on amino acids [21], have lower cmc values and are more efficient in reducing surface tension than their monocatenery homologs. It seems that the hydrophobicity associated with the second alkyl chain and hydrophobic spacer chain largely compensates for the polarity conferred by the second head group.

**Figure 9:**
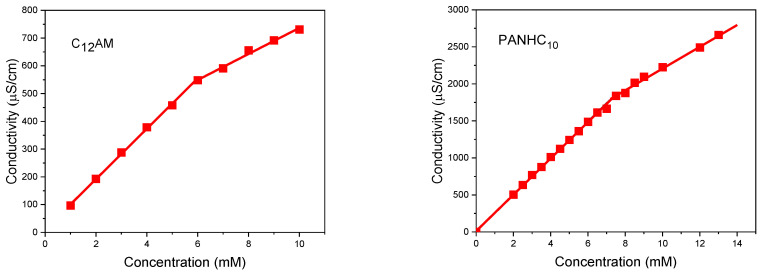
Specific conductivity-concentration curves for C_12_AM and PANHC_10_.

The cmc values of these amino acid-based surfactants were also determined by conductivity. As shown in Figure 9, in C_12_AM and PANHC_10_ the conductivity depends on the surfactant concentration. Similar plots were obtained for the other surfactants (see Supplementary Materials). The specific conductivity values fit into two straight lines with different slopes, and an abrupt change in the slopes corresponds to the cmc. In the low concentration range, the increase in conductivity (κ) is due to the increase in free cationic surfactant monomers and counterions. After the cmc, the dependence of κ on concentration has a smaller slope due to lower micelle mobility and the binding of a fraction of the counterions to the micellar surface [34]. The values obtained using this technique agree with those obtained by fluorescence measurements (Table 2) and confirm the cmc values obtained for the new surfactants. Table 2 also shows the retention time (Rt) obtained by HPLC measurements. This parameter follows the same tendency as the cmc, increasing with the reduction in polarity of the head group while the alkyl chain stays the same, and also increasing with the hydrophobicity of the alkyl chain, with the polar group remaining the same.

#### 3.1.7. Antimicrobial Activity

In the search for novel, safe and efficient antimicrobials, considerable attention has been focused on the development of cationic amino acid-based surfactants, whose antimicrobial activity depends on different structural features. In the present study, we prepared new amino acid-based surfactants in which a head group containing one or two amino acids and a hydrophobic alkyl chain were assembled using an amide bond. The different molecular structures prepared allowed us to determine the effect of several parameters (cationic charge density, alkyl chain length and position, and presence of aromatic groups) on the antimicrobial efficiency of these small amphiphilic molecules.

The antimicrobial activity of the new amino acid-based surfactants was tested against a range of representative Gram-positive bacteria (*S. aureus* ATCC 6538, *S. epidermidis* ATCC 1222, *B. subtilis* ATCC 6633, *L. monocytogenes* ATCC 15313) and Gram-negative bacteria (*E. coli* ATCC 8739, *P. aeruginosa* ATCC 9027, *A. baumannii* ATCC 19606) by determining their minimum inhibitory concentration (MIC), the concentration of surfactants required to completely inhibit the growth of microorganism, and the minimum bactericidal concentration (MBC), the concentration of surfactants required to kill microorganisms.

**Figure 10:**
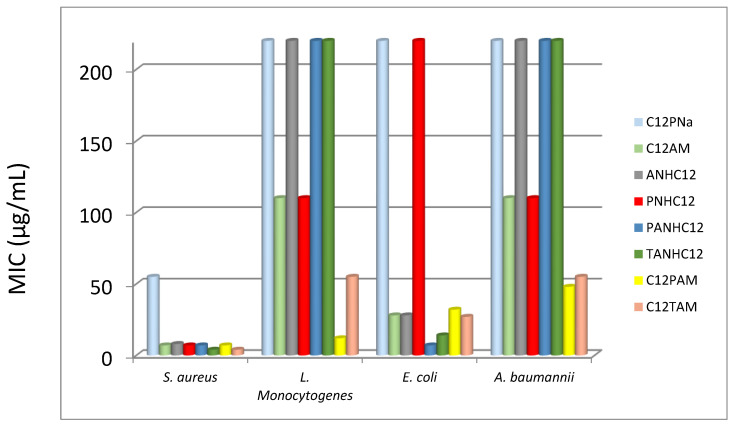
Effect of the polar head on the antimicrobial activity of the C_12_ derivatives.

Table 3 and Figure 10 show the MIC values corresponding to the monocatenary surfactants with a similar alkyl chain (C_12_ derivatives) but different polar group. The anionic analog only exhibited moderate activity against two of the bacteria, the Gram-positive *S. aureus* and *S. epidermidis.* As expected, the cationic charge on the polar head was found to be a key structural determinant of the antimicrobial activity of the amino acid-based surfactants.

The adsorption of cationic surfactants on bacterial cell membranes, usually negatively charged, is enhanced by electrostatic interactions [35]. The monocatenary surfactants with one amino acid on the polar head, PNHC_12_ (cationic charge on the α-amino group of phenylalanine, pKa = 5.5), ANHC_12_ (two cationic charges, one on the protonated amino group of arginine, pKa = 5.7, and the other on the protonated guanidine moiety, pKa = 10) and C_12_AM (one cationic charge on the protonated guanidine group), exhibited similar activity against the Gram-positive bacteria. However, the MIC values against Gram-negative bacteria were quite different: whereas C_12_AM and ANHC_12_ showed comparable activity against *P. aeruginosa E. coli*, PNHC_12_ was roughly five-fold less active than either of them. This behavior could be attributed to the higher pKa of the protonated guanidine group. Notably, the antimicrobial activity was determined at pH = 7.2, the value at which most of the PNHC_12_ molecules are expected to be unprotonated. Similarly, at pH = 7, almost all ANHC_12_ molecules have only one positive charge on the guanidine group. This highlights the role of the cationic charge density and consequently the pKa values in the antibacterial efficiency of cationic surfactants. pKa-dependent antimicrobial activity was also observed in other pH-sensitive amino acid-based surfactants, being good when pKa values were higher than nine and less so below seven, especially against Gram-negative bacteria [18]. These results are of great interest, given the growing demand for antibacterial agents effective against opportunistic human pathogens such as *P. aeruginosa* and *E. coli*. *P. aeruginosa* is currently a leading cause of hospital-acquired infections and shows resistance to almost all clinically approved antibiotics [36].

To elucidate the role of polar head aromatic groups in antibacterial activity, we synthesized series of PANHC_n_, TANHC_n_, C_n_TAM and C_n_PAM. All these compounds have a protonated guanidine group and an aromatic group of tryptophan or phenylalanine amino acids. The incorporation of an aromatic core did not affect the activity of C_12_AM against Gram-positive bacteria, as compounds C_12_TAM and C_12_PAM displayed comparable MIC values against all the tested Gram-positive bacteria. However, these surfactants showed higher activity against *L. monocytogenes* and *A. baumannii*. Different results have been reported regarding the effect of the aromatic group on antibacterial activity. For example, benzalkonium bromide bearing a benzyl group on the head group was more active than its counterpart dodecyl trimethyl ammonium by around one order of magnitude [37,38].

Ghosh et al. observed that in surfactants with short alkyl chains, the aromatic group has a stronger influence than the hydrophobic chain on antimicrobial effectivity, and vice versa in surfactants with long alkyl chains [35]. Cationic antimicrobial surfactants containing two amino acids on the polar head, tryptophan-proline, phenylalanine-proline, and phenylalanine-tryptophan, markedly inhibited both Gram-positive (MIC between 5 and 150 μg/mL) and Gram-negative bacteria (MIC between 5 and 150 μg/mL) [39]. It seems that the ability of cationic surfactants to disrupt bacterial membranes can be enhanced by the incorporation of two amino acids on the polar head, especially when this hydrophilic moiety contains an aromatic amino acid combined with a cationic charge. In fact, arginine and tryptophan are found in high proportion in numerous antimicrobial peptides [40]. However, Taleb et al. found that the incorporation of phenyl groups into gemini surfactants reduced their antimicrobial activity [41]. These reports suggest that antimicrobial properties can be enhanced by the incorporation of hydrophobic groups, including aromatic ones, but they are reduced if too many are added.

Increasing the cationic charge density on the polar head is one of the most frequently used strategies to boost surfactant antibacterial properties [42]. Activity against Gram-negative bacteria of lysine-based surfactants was found to decrease with the pKa values and cationic charge density [24]. In contrast, TANHC_12_ and PANHC_12_, which have two positive charges on the polar head, exhibited lower activity against Gram-negative bacteria than their homologs C_12_PAM and C_12_TAM, with only one positive charge. This anomalous behavior can be ascribed to the low pKa value of the cationic charge on the protonated amine group of TANHC_12_ and PANHC_12_.

Our results indicate that the alkyl chain position also affects antimicrobial activity. C_12_PAM and C_12_TAM, in which the alkyl chain is bonded to the α-amino group of the amino acid, were more efficient against two problematic pathogens, *L. monocytogenes* and *A. baumannii*, compared to PANHC_12_ and TANHC_12_, in which it is bonded to the carboxylic group. This behavior could be attributed to the different polarity of their polar heads. Nα-derivatives contain two amide bonds on the polar head, whereas the O-alkyl homologs have one amide bond and one partially protonated amine group at pH = 7.0. Amide appears to be more polar than amine, and consequently, it can both hydrogen bond and accept hydrogen bonds. The better activity of C_12_PAM and C_12_TAM suggests that the presence of two amide bonds provides the optimum hydrophobic/hydrophilic balance to interact with bacterial membranes. On other hand, this dissimilar polarity can promote the self-assembly of structures of diverse shape and stability, parameters that affect the antimicrobial activity of cationic surfactants [43,44].

From these results, it can be concluded that the amino acid-based surfactants were effective against Gram-positive bacteria, regardless of the type of polar head group, but changes in the polar head and alkyl chain position significantly affected activity against *L. monocytogenes* and Gram-negative bacteria. It should be stressed that effective agents against these microorganisms are urgently needed. The incidence of listeriosis (an infection caused by *L. monocytogenes*) in developed countries has grown significantly due to several factors: a higher proportion of immunocompromised persons, increased use of refrigeration to extend the shelf life of perishable food, and growing consumer demand for refrigerated or frozen foods. Cationic surfactants usually exhibit lower activity against Gram-negative bacteria, whose outer membranes are less permeable due to the presence of peptidoglycan, glycerophospholipids and lipopolysaccharides, which hinders electrostatic interactions with cationic amphiphiles [45]. Consequently, to prepare surfactants with good activity against Gram-negative bacteria, more fine-tuning is required to obtain an appropriate hydrophobic-hydrophilic balance.

The alkyl chain length, associated with the hydrophobic character of amphiphilic compounds, is another structural parameter that affects antimicrobial efficiency. Table 4 shows the MIC values of the tryptophan and phenylalanine derivatives with variable alkyl chains. The derivative with the shortest alkyl chain, PANHC_8_, was found to be moderately active against three of the four Gram-positive bacteria tested (*S. aureaus, S. epidermidis, B. subtilis*) and non-active against *L. monocytogenes* and the Gram-negative *E. coli, P. aeruginosa* and *A. baumannii*. Increasing the chain length improved the antibacterial activity. In the PANHC_n_ and TANHC_n_ series, C_12_ derivatives were the most effective, whereas the longer alkyl chain of the C_14_ derivatives compromised activity against some microorganisms, including *P. aeruginosa*. Similar behavior was observed for C_n_TAM and C_n_PAM, where antibacterial efficiency was improved when the alkyl chain length was increased from 10 to 12 carbons; moreover, the difference between C_10_ and C_12_ was more striking in these series. The most potent C_12_ derivatives were C_12_TAM and C_12_PAM, given that they were active against all the bacteria tested, with MIC values between 4 and 64 μg/mL. The structure–activity study indicates that the optimum chain length for these families of amino acid-based surfactants lies between dodecyl and tetradecyl. A similar performance has been reported for numerous series of surfactants. Antibacterial activity and other biological properties usually exhibit a non-linear dependence on the alkyl chain length, known as the cutoff effect; efficiency tends to reach a peak with C_12_ and C_14_ derivatives, gradually decreasing thereafter. Different reasons have been postulated to explain this behavior. Inacio et al. [46] suggest that the cmc values of surfactants play an important role. The formation and elimination of free volume in the bilayer hydrophobic region of the bacterial membrane are also thought to be involved [13,47,48]. Additionally, recent studies show that C_12_PAM and C_12_TAM exhibit very good antifungal and antibiofilm activity against some resistant candida strains [49]. The antimicrobial activity of these surfactants was higher than that reported for cationic surfactants with only phenylalanine [27], histidine [31], or tyrosine [27] on the polar head. Likewise, similar antimicrobial efficiency has been described for peptoid mimics containing two amino acids on the polar head (phenylalanine-proline, tryptophan-proline and tryptophan-phenylalanine) [39]. The greater efficacy of surfactants bearing two amino acids on the hydrophilic moiety can be ascribed to the larger polar head, which increases both the molecular area at the membrane–water interface and the cone angle, thus enhancing membrane damage. Moreover, in the aforementioned dipeptide, the delocalized cationic charge in the guanidine group facilitated transmembrane translocation [50]. It seems that effectivity increases when the polar head contains an aromatic as well as a cationically charged amino acid. In fact, arginine and tryptophan are found in very high proportions in several antimicrobial peptides [51].

The in vitro antibacterial activity of a gemini surfactant based on arginine-tryptophan was also investigated, and the results are provided in Table 4. The MIC values indicate that this compound is an effective bacteriostatic agent and was also able to kill problematic Gram-negative bacteria (*E. coli*, *P. aeruginosa* and *A. baumannii*) at low concentrations. This gemini surfactant was clearly more active than the most effective monocatenary C_12_TAM. These results are not surprising, as studies have shown that gemini Dabco surfactants, gemini surfactants of the bisQuat type [37,52,53], arginine-based dimeric surfactants [54] and histidine-based gemini amphiphiles are more active than their corresponding single-chain derivatives [34]. The antimicrobial activity of cationic surfactants is governed by their electrostatic interaction with anionic compounds in biological membranes as well as hydrophobic interactions with hydrophobic lipid bilayers. The presence of two cationic charged head groups may induce a faster electrostatic interaction with negatively charged cell membrane molecules (lipopolysaccharides in Gram-negative bacteria and teichoic acids in Gram-positive bacteria). Accordingly, antibacterial activity of cationic surfactants is reported to increase with the number of positive charges, due to the multidentate interaction with bacterial membranes. Studies of penetration kinetics indicate that arginine-based gemini surfactants insert more readily into anionic DPPG than zwitterionic DPPC lipid monolayers, suggesting that lipid-antimicrobial interaction is mostly governed by electrostatic forces. Additionally, the two alkyl chains can facilitate hydrophobic interactions with lipid bilayers [55]. These results show that by varying both the alkyl chain length and its position, we were able to fine-tune surfactant antimicrobial efficiency against some recalcitrant microbes such as *L. monocytogenes* and Gram-negative bacteria.

The action of an antibacterial agent on a bacterial strain can also be characterized by the MBC, the dosage at which >99.9% of bacteria are killed. The MBC of all the synthesized compounds is shown in Table 5, where it can be observed that the MBC/MIC ratio ranged from 1 to 2, indicating that these surfactants exhibited a potent bactericidal effect against the tested microorganisms.

To explore the antimicrobial mechanisms of these amino acid-based surfactants, two problematic species were chosen, the Gram-positive S. *aureus* and Gram-negative *P. aeruginosa*. The experiments were carried out using one of the most powerful antimicrobial surfactants prepared (C_12_PAM).

**Figure 11:**
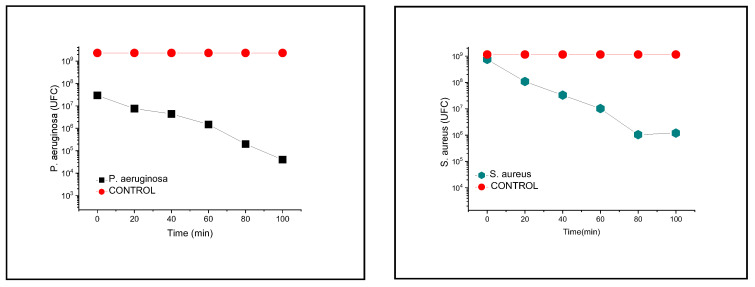
Effect of C_12_PAM on the bacterial count of *P. aeruginosa* and *S. aureus* at the respective MIC.

Figure 11 shows the reduction in bacterial growth on exposure to C_12_PAM at the MIC. The reduction in viability of *P. aeruginosa* and *S. aureus* after 20 min of contact was 77% and 85%, respectively. The maximum reduction was observed at 100 min for *P. aeruginosa* (more than 99%) and at 80 min for *S. aureus*. A notable and rapid reduction in cell viability has previously been observed in different bacteria treated with lauroyl arginine ethyl ester (LAE) at the MIC [56,57,58].

**Figure 12:**
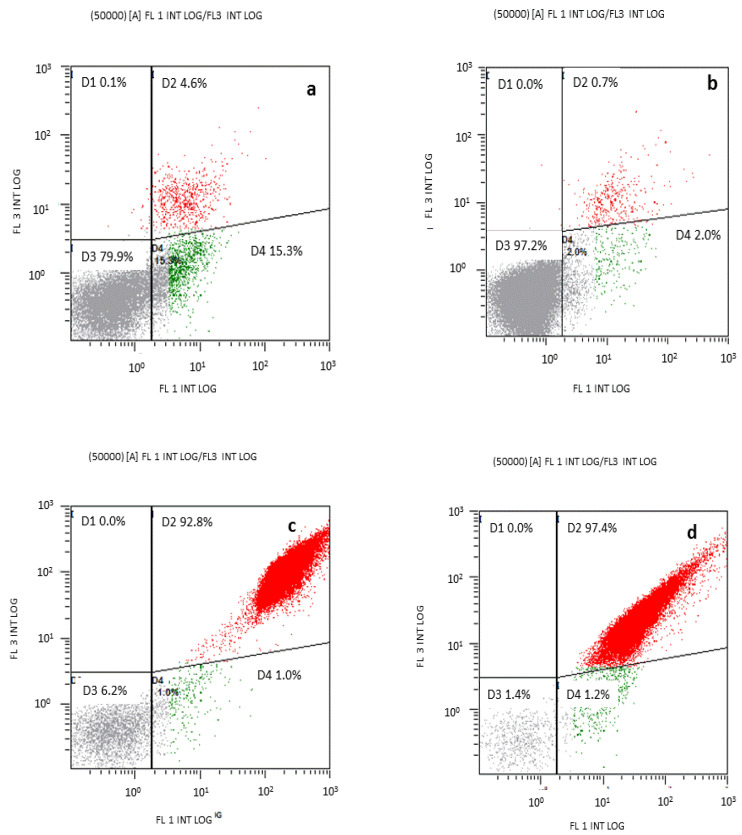
Dual parameters of stained *S. aureus* and *P. aeruginosa* cells (bis-oxonol and propidium iodide): (**a**) *P. aeruginosa* control, (**b**) *S. aureus* control (**c**) *P. aeruginosa* treated with C_12_PAM, and (**d**) *S. aureus* treated with C_12_PAM.

Flow cytometry has been widely used to measure physical and chemical parameters in cells. Fluorescence signals obtained by labelling cells with specific probes allow the detection of certain indicators of cell functionality. In the present study, the loss of membrane potential was evaluated by the anionic dye bis-oxonol, which binds to lipid-rich components after entering depolarized cells. Cell membrane permeability was assessed by the positively charged dye PI, which enters the cell through permeabilized membranes and intercalates between RNA and DNA. Figure 12 shows the cell damage caused by C_12_PAM in the two bacteria tested. When *E. coli* and *S. aureus* were treated with C_12_PAM in the presence of PI and bis-oxonol, three subpopulations of cells were observed: (a) unstained intact cells (D3 in grey), (b) depolarized cells stained with bis-oxonol (D4 in green) and (c) cells with dissimilar degrees of permeabilized membranes stained with PI (D1, D2 in red). When C_12_PAM was added to an *S. aureus* culture, only 1.4% of cells remained intact and 1.2% of cells retained bis-oxonol, indicating a depolarized membrane. However, almost the entire population (97.4%) retained PI, indicating cell death by membrane permeabilization. These results agree with the almost 100% reduction in viability depicted in Figure 11. The same behavior was found in the Gram-negative *P. aeruginosa* treated with the same surfactant.

Given its amphiphilic nature, this amino acid-based surfactant operates by mechanisms similarly to those described for quaternary ammonium compounds (QUATs). Electrostatic interactions between the positive charge of cationic surfactants and the negatively charged bacterial cellular membranes are followed by the permeation of the hydrophobic chains into the intermembrane region, giving rise to a leakage of cytoplasmatic material and cell lysis. By targeting bacterial cell membranes, these surfactants can affect a broad spectrum of microorganisms. This mechanism of action is supported by the higher activity exhibited against Gram-positive than Gram-negative bacteria. The former have a single phospholipid cellular membrane and a thicker cell wall composed of peptidoglycan, whereas the latter are encapsulated by two cellular membranes and a rather thin peptidoglycan cell wall. Consequently, membrane-targeting antimicrobials exhibit lower activity against Gram-negative bacteria [14]. The mode of action of the cationic LAE has been found to depend on the concentration: at the MIC, membrane permeabilization was the main mechanism responsible for cell death, whereas at 2/3 MIC a notable percentage of cells showed membrane depolarization [57]. When *P. digitatum* and *P. carotovorum* were treated with LAE, the permeability of their cell membranes increased dramatically, and cellular material was released from the inactivated microorganisms [59] Recent studies show that the antifungal properties of C_12_PAM seem to be based on its ability to damage and structurally change cell membranes [49].

#### 3.1.8. Aerobic Biodegradability

Biodegradation of amino acid-based surfactants was assessed by applying the CO_2_ Headspace Test (OECD 310), a reference method approved by the European Union to evaluate the aerobic biodegradability of surfactants in European legislation. The extent of biodegradation is expressed as a percentage of the theoretical maximum generation of inorganic carbon based on the initial surfactant amount added. Table 6 displays the biodegradation percentages for the amino acid-based surfactants and the 95% confidence interval for the 28-day biodegradation mean.

All the amino acid-based surfactants underwent some degree of mineralization under the test conditions over the 28-day period (Table 6), but the percentage of biodegradation differed significantly, ranging from 10 to 79% (Table 6). The biocidal activity demonstrated in most of the investigated amino acid-based surfactants could negatively affect their biodegradation. To determine if the low degradation rate of some of the surfactants could be attributed to their potential toxicity against the inoculum, inhibitory checks were included in the biodegradation test design. The toxicity of the amino acid-based surfactants for aerobic microorganisms was assessed by determining the inhibition of the biodegradation of the reference substance (sodium benzoate) in binary mixtures with the test surfactant (Table 7).

**Figure 13:**
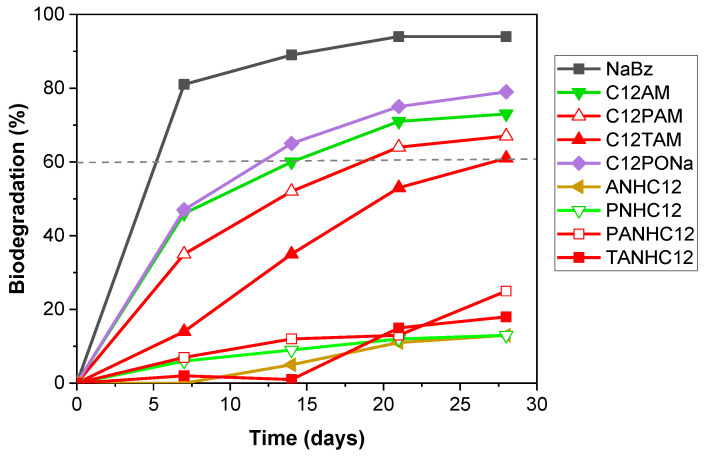
Biodegradation curves of amino acid-based surfactants containing one amino acid on the polar head and one hydrocarbon chain of 12 carbons.

**Figure 14:**
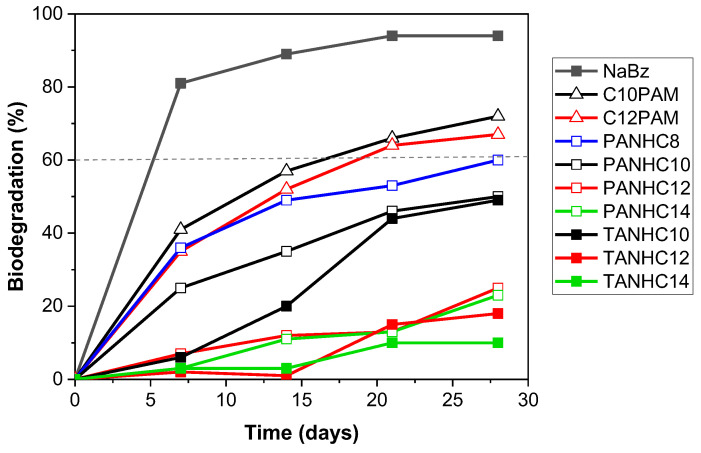
Biodegradation curves of amino acid-based surfactants with two amino acids on the polar head and one alkyl chain of different lengths (C_8_–C_14_).

The amino acid-based surfactants were found not to inhibit the inoculum activity and did not present a high percentage of inhibition (Table 7), all the values being clearly below 25%, which is the minimum value indicating inhibitory effects in this standard biodegradation test [60]. Therefore, none of the surfactants negatively affected the activity of the aerobic microorganisms and the incomplete degradation observed is not attributable to biocidal effects but solely to non-amenability to the microbial attack under the test conditions. Biodegradation curves for amino acid-based surfactants with one amino acid on the polar head and a 12-carbon alkyl chain or with two amino acids on the polar head and an alkyl chain of different lengths are shown in Figure 13 and Figure 14, respectively.

Among monocatenary surfactants with one amino acid on the polar head and a 12-carbon alkyl chain (Figure 13), C_12_AM and C_12_PNa exceeded the 60% threshold and can be regarded as readily biodegradable compounds. In contrast, the biotransformation of ANHC_12_ and PNHC_12_ was a modest 13% at the end of the test. Among surfactants containing two amino acids on the polar head group and different alkyl chain lengths (Figure 14), C_n_PAM and C_n_TAM underwent more than 60% biodegradation and can be classified as easily biodegradable, whereas the PANHC_n_ and TANHC_n_ homologs did not pass this threshold. It should be noted that in the latter surfactants, biological oxidation decreased significantly with increasing alkyl chain length, biodegradation values varying from ≥50% for C_8_–C_10_ to ≤ 25% for C_12_–C_14_ homologs (Figure 14). This indicates that the hydrophobicity of the hydrocarbon chain of the surfactant plays a crucial role in its biodegradation.

Hydrophilic and hydrophobic moieties of amino acid-based surfactants are linked by a primary amide bond susceptible to base- or enzyme-catalyzed hydrolysis. As amidases are widespread in nature [61], it is generally accepted that the aerobic biodegradation pathway of primary amides involves initial hydrolysis of the amide bond [62].

Our results underline the important role of the alkyl chain linkage with the polar head group in the biodegradation of monocatenary amino acid-based surfactants. Thus, if the hydrocarbon chain is linked to the carbonyl carbon atom of the amide functional group (C_12_AM, C_12_PONa, C_n_PAM and C_n_TAM), the surfactants undergo rapid and complete aerobic oxidation (>60%) (Table 6, Figure 13 and Figure 14). However, when the hydrocarbon chain is directly linked to the N atom of the amide group (ANHC_12_, PNHC_12_, PANHC_n_ and TANHC_n_), the degree of biodegradation varies from low to moderate and does not reach the 60% pass level of the biodegradation test (Table 6, Figure 13 and Figure 14).

The biodegradation results can be explained by the following mechanisms. In surfactants with the hydrocarbon chain attached to the carbonyl carbon atom of the amide group, hydrolysis of the amide bond leads to the release of fatty acids and amino acids. The fatty acids are then rapidly broken down by the ubiquitous β-oxidation process [63]. The expected rapid and complete oxidation was confirmed by the results. In surfactants with the hydrophobic chain attached to the N atom, hydrolysis of the amide functional group results in the formation of primary amines and amino acids. Ultimate biodegradation depends on the biodegradability of the released fatty amines. Although primary amines are generally considered to be readily or inherently biodegradable (ECCC 2021) [64], fatty amines are relatively insoluble and have a high tendency to adsorb onto glass surfaces [65] which reduces their availability to microorganisms (EC2008). This could be the main reason for the partial oxidation observed for these surfactants in the test conditions. Moreover, the reduction in biodegradation with increasing hydrophobicity supports that the lower availability of longer-chain amines generated by amide bond hydrolysis was responsible for the incomplete surfactant degradation.

## 4. Conclusions

Biodegradable and antimicrobial cationic surfactants were prepared with systematic structural modifications (head group architecture and hydrophobic moiety) using renewable raw materials such as amino acids, fatty acids, and fatty amines. The surfactant cmc depended mainly on the hydrophobic/hydrophilic balance, but antimicrobial activity and biodegradability were also affected by the type of amino acid in the polar head and the position of the hydrophobic moiety. Antimicrobial efficacy was improved by the presence of an aromatic amino acid and the optimum alkyl chain proved to be C_12_. Among N^α^-acyl surfactants, those with the alkyl chain linked to the α-amino group of the amino acid exhibited better activity against problematic microorganisms such as *L. monocytogenes* and Gram-negative bacteria. The surfactant mechanism of action involves the disintegration of bacterial cell membranes and release of cellular material. All the investigated amino acid-based surfactants underwent biological degradation under aerobic conditions, the extent of which was crucially dependent on the attachment of the hydrocarbon chain to the polar head group. Thus, the biodegradation of N^α^-acyl amino acid surfactants was rapid and complete (>60%), whereas surfactants with the hydrocarbon chain attached to the amino acid carboxyl group generally underwent a partial biological oxidation related to the alkyl chain length. Incomplete degradation of the homologues with the longest alkyl chains is attributable to the reduced bioavailability of the fatty amines released in the initial hydrolysis of the amide bond.

The development of bacterial resistance to some of these amino acid-based surfactants seems a priori improbable for two main reasons: (a) their mode of action, the disintegration of bacterial membranes, is nonspecific and fundamental to bacterial survival, and (b) high levels of biodegradation render their accumulation in the environment unlikely, and therefore, bacterial populations would not be subjected to lethal doses of these surfactants. Thus, these highly effective biodegradable surfactants show great potential as novel antibacterial agents for future biomedical applications. 

## Figures and Tables

**Table 1:** pKa values of the amino acid-based surfactants.

Compound	pKa1	Surf.	pKa1	Surf.	pKa1	Surf.	pKa1	pKa2
C_10_TAM	-	PANHC_8_	6.9	TANHC_10_	7.2	C_12_AM		9.3
C_12_TAM	-	PANHC_10_	6.9	TANHC_12_	7.8	ANHC_12_	5.3	-
C_10_PAM	-	PANHC_12_	6.1	TANHC_14_	7.0	PNHC_12_	5.5	-
C_12_PAM	-	PANHC_14_	5.9	C_3_(TANHC_8_)_2_				

**Table 2:** The cmc values (mM) and retention time (Rt, min) of the amino acid-based surfactants.

Compound	Cmc Fluorescence (mM)	Cmc Conductivity (mM)	Rt (min)
C_12_AM	4	6	12.4
ANHC_12_	7.2	9.4	10.8
PNHC_12_	0.9	1.2	14.8
C_12_PNa	non soluble	non soluble	
C_10_PNa	2.9		
C_10_PAM	2.6	3.7	13.4
C_12_PAM	1.1	1.2	15.4
C_10_TAM	1.6	-	14.4
C_12_TAM	0.6	0.45	15.9
PANHC_8_	11.5	10.1	9.2
PANHC_10_	5.3	7.2	11.8
PANHC_12_	1.6	2.2	13.5
PANHC_14_	0.45	0.7	15.0
TANHC_10_	2.5	1.7	13.5
TANHC_12_	0.6	0.6	15.1
TANHC_14_	0.2	0.3	16.2
C_3_(TANHC_8_)	0.2	-	17.1

**Table 3:** MIC values (μg/mL) of the C_12_ derivatives: SA (*S. aureus*), SE (*S. epidermidis*), BS (*B. subtilis*), LM (*L. monocytogenes*), EC (*E. coli*), PA (*P. aeruginosa*), AB (*A. baumannii*).

	SA	SE	BS	LM	EC	PA	AB
C_12_AM	8	8	16	128	32	32	128
ANHC_12_	8	8	4	>256	28	28	>256
PNHP_12_	8	4	8	128	>256	>256	128
C_12_PNa	64	128	>256	>256	>256	>256	>256
PANHC_12_	8	8	8	>256	8	64	>256
TANHC_12_	4	4	4	256	16	32	256
C_12_TAM	4	4	4	16	32	32	64
C_12_PAM	8	4	4	64	32	64	64

**Table 4:** MIC values (μg/mL) of surfactants with different alkyl chain: SA (*S. aureus*), SE (*S. epidermidis*), BS (*B. subtilis*), LM (*L. monocytogenes*), EC (*E. coli*), PA (*P. aeruginosa*), AB (*A. baumannii*).

	SA	SE	BS	LM	EC	PA	AB
PANHC_8_	64	32	8	>256	>256	>256	>256
PANHC_10_	16	8	8	>256	64	128	>256
PANHC_12_	8	8	8	>256	8	64	>256
PANHC_14_	8	8	8	32	16	>256	>256
TANHC_10_	4	4	8	256	32	32	256
TANHC_12_	4	4	4	256	16	32	128
TANHC_14_	8	4	4	256	16	128	256
C_10_TAM	16	16	16	-	128	128	-
C_12_TAM	4	4	4	16	32	32	64
C_10_PAM	16	16	32	256	64	64	128
C_12_PAM	8	4	4	64	32	64	64
C_3_(TANHC_8_)_2_	2	4	2	4	32	32	4

**Table 5:** MBC values (μg/mL) SA (*S. aureus*), SE (*S. epidermidis*), BS (*B. subtilis*), LM (*L. monocytogenes*), EC (*E. coli*), PA (*P. aeruginosa*), AB (*A. baumannii*).

	SA	SE	BS	LM	EC	PA	AB
C_12_AM							
ANHC_12_	8	16	32	256	256	64	>256
PNHC_12_	8	8	8	>256	>256	>256	256
PANHC_8_	64	32	16	>256	>256	>256	>256
PANHC_10_	32	32	64	>256	>256	256	>256
PANHC_12_	8	16	8	>256	16	64	>256
PANHC_14_	8	8	8	256	16	>256	>256
TANHC_10_	8	8	8	256	32	128	256
TANHC_12_	8	4	4	256	32	32	256
TANHC_14_	6	8	8	256	64	256	256
C_10_TAM							
C_12_TAM	4	4	8	64	32	32	64
C_10_PAM	32	32	64	256	128	128	>256
C_12_PAM	16	8	32	64	64	128	128
C_3_(TANHC_8_)_2_	4	8	4	8	64	128	16

**Table 6:** Biodegradation study (CO_2_ Headspace test) of amino acid-based surfactants. Percentages of biodegradation of surfactants and the reference substance, sodium benzoate (NaBz) (95% confidence interval over 28 days based on four replicates).

Compound	Biodegradation (%)			
	7 Days	14 Days	21 Days	28 Days
NaBz	81	89	94	94 ± 2.3
C_12_AM	46	60	71	71 ± 6.1
C_12_PNa	47	65	75	79 ±4.5
ANHC_12_	0	5	11	13 ± 0.5
PNHC_12_	6	9	12	13 ± 2.6
C_10_PAM	41	57	66	72 ± 5.8
C_12_PAM	35	52	64	67 ± 4.7
C_12_TAM	14	35	53	61 ± 5.3
PANHC_8_	36	49	53	60 ± 7.5
PANHC_10_	25	35	46	50 ± 6.1
PANHC_12_	7	12	13	25 ± 5.7
PANHC_14_	3	11	13	23 ± 9.9
TANHC_10_	6	20	44	49 ± 4.2
TANHC_12_	2	1	15	18 ± 4.1
TANHC_14_	2	3	10	10 ± 4.8

**Table 7:** Inhibition of sodium benzoate (reference substance) by amino acid-based surfactants.

Compound	Concentration (mg C/L)	Inhibition (%)
NaBz + C_12_AM	20 +15	0
NaBz + C_12_PONa	20 +15	0
NaBz + ANHC_12_	20 +15	0
NABz + PNHC_12_	20 +15	3 ± 2
NaBz + C_10_PAM	20 + 15	0
NaBz + C_12_PAM	20 + 15	0
NaBz + C_12_TAM	20 + 15	0
NaBz + PANHC_8_	20 + 15	0
NaBz + PANHC_10_	20 + 15	0
NaBz + PANHC_12_	20 + 15	7 ± 3
NaBz + PANHC_14_	20 + 15	0
NaBz + TANHC_10_	20 + 15	0
NaBz + TANHC_12_	20 + 15	9 ± 3
NaBz + TANHC_14_	20 + 15	14 ± 4

## Data Availability

The data presented in this study are available in the article and Supplementary Materials.

## Supplementary Materials

The following are available online at https://www.mdpi.com/article/10.3390/pharmaceutics14122602/s1, Figure S1: HPLC, ESI_MS, ^1^HNMR, ^13^CNMR and Dept of C_12_AM. Figure S2: HPLC, ESI_MS, ^1^HNMR, ^13^CNMR and Dept of ANHC_12_. Figure S3: HPLC, ESI_MS, ^1^HNMR, ^13^CNMR and Dept of C_12_PNa. Figure S4: HPLC, ESI_MS, ^1^HNMR, ^13^CNMR and Dept of PNHC_12_. Figure S5: HPLC, ESI_MS, ^1^HNMR, ^13^CNMR and Dept of C_10_PAM. Figure S6: HPLC, ESI_MS, ^1^HNMR, ^13^CNMR and Dept of C_12_PAM. Figure S7: HPLC, ESI_MS, ^1^HNMR, ^13^CNMR and Dept of C_10_TAM. Figure S8: HPLC, ESI_MS, ^1^HNMR, ^13^CNMR and Dept of C_12_TAM. Figure S9: HPLC, ESI_MS, ^1^HNMR, ^13^CNMR and Dept of Cbz-PANHC_8_. Figure S10: HPLC, ESI_MS, ^1^HNMR, ^13^CNMR and Dept of Cbz-PANHC_10_. Figure S11: HPLC, ESI_MS, ^1^HNMR, ^13^CNMR and Dept of Cbz-PANHC_12_. Figure S12: HPLC, ESI_MS, ^1^HNMR, ^13^CNMR and Dept of Cbz-PANHC_14_. Figure S13: HPLC, ESI_MS, ^1^HNMR, ^13^CNMR and Dept of PANHC_8_. Figure S14: HPLC, ESI_MS, ^1^HNMR, ^13^CNMR and Dept of PANHC_10_. Figure S15: HPLC, ESI_MS, ^1^HNMR, ^13^CNMR and Dept of PANHC_12_. Figure S16: HPLC, ESI_MS, ^1^HNMR, ^13^CNMR and Dept of PANHC_14_. Figure S17: HPLC, ESI_MS, ^1^HNMR, ^13^CNMR and Dept of Cbz-TANHC_8_. Figure S18: HPLC, ESI_MS, ^1^HNMR, ^13^CNMR and Dept of Cbz-TANHC_10_. Figure S19: HPLC, ESI_MS, ^1^HNMR, ^13^CNMR and Dept of Cbz-TANHC_12_. Figure S20: HPLC, ESI_MS, ^1^HNMR, ^13^CNMR and Dept of Cbz-TANHC_14_. Figure S21: HPLC, ESI_MS, ^1^HNMR, ^13^CNMR and Dept of TANHC_8_. Figure S22: HPLC, ESI_MS, ^1^HNMR, ^13^CNMR and Dept of TANHC_10_. Figure S23: HPLC, ESI_MS, ^1^HNMR, ^13^CNMR and Dept of TANHC_12_. Figure S24: HPLC, ESI_MS, ^1^HNMR, ^13^CNMR and Dept of TANHC_14_. Figure S25: HPLC, ESI_MS, ^1^HNMR, ^13^CNMR and Dept of C_3_(TANHC_8_)_2_. Figure S26: Specific conductivity–concentration curves for the amino acid-based surfactants.
