# Supplementary material for: Cationic Surfactants Based on Arginine-Phenylalanine and Arginine-Tryptophan: Synthesis, Aggregation Behavior, Antimicrobial Activity, and Biodegradation
Source: Pharmaceutics. 2022 Nov 25;14(12):2602. doi: 10.3390/pharmaceutics14122602 (PMC9784262; doi:10.3390/pharmaceutics14122602)
Supplement: Supplementary file 1 [file pharmaceutics-14-02602-s001.zip › pharmaceutics-2048241-supplementary.pdf]

ELECTRONIC SUPPLEMENTARY INFORMATION

Cationic Surfactants Based on Arginine-Phenylalanine and Arginine-Tryptophan: Synthesis, Aggregation Behavior, Antimicrobial Activity, and Biodegradation

L.Pérez, M.T. Garcia, A. Pinazo, E. Pérez, Z. Hafidi, E. Bautista

Department of Surfactants and Nanobiotechnology, IQAC-CSIC, c/Jordi Girona, 18-26, 08034 Barcelona, Spain

CONTENT

Figure S1. HPLC, ESI-MS, ^{1}H NMR, and ^{13}C NMR of C_{12}AM
Figure S2. HPLC, ESI-MS, ^{1}H NMR, and ^{13}C NMR of $ANHC_{12}$
Figure S3. HPLC, ^{1}H NMR, ^{13}C NMR and Dept of C_{12}PNa
Figure S4. HPLC, and ^{1}H NMR, of $PNHC_{12}$
Figure S5. HPLC, ESI-MS, ^{1}H NMR,^{13}C NMR and Dept of C_{10}PAM
Figure S6. HPLC, ESI-MS, ^{1}H NMR,^{13}C NMR and Dept of C_{12}PAM
Figure S7. HPLC,^{1}H NMR,^{13}C NMR and Dept of C_{10}TAM
Figure S8. HPLC, ESI-MS, ^{1}H NMR,^{13}C NMR and Dept of C_{12}TAM
Figure S9. HPLC, ESI-MS, ^{1}H NMR,^{13}C NMR and Dept of Cbz-$PANHC_{8}$
Figure S10. HPLC, ESI-MS, ^{1}H NMR,^{13}C NMR and Dept of Cbz-$PANHC_{10}$
Figure S11. HPLC, ESI-MS, ^{1}H NMR,^{13}C NMR and Dept of Cbz-$PANHC_{12}$
Figure S12. HPLC, ESI-MS, ^{1}H NMR,^{13}C NMR and Dept of Cbz-$PANHC_{14}$
Figure S13. HPLC, ESI-MS, ^{1}H NMR,^{13}C NMR and Dept of $PANHC_{8}$
Figure S14. HPLC, ESI-MS, ^{1}H NMR,^{13}C NMR and Dept of $PANHC_{10}$
Figure S15. HPLC, ESI-MS, ^{1}H NMR,^{13}C NMR and Dept of $PANHC_{12}$
Figure S16. HPLC, ESI-MS, ^{1}H NMR,^{13}C NMR and Dept of $PANHC_{14}$
Figure.S17. HPLC, ESI-MS, ^{1}H NMR,^{13}C NMR and Dept of Cbz-$TANHC_{8}$
Figure S18. HPLC, ESI-MS, ^{1}H NMR,^{13}C NMR and Dept of Cbz-$TANHC_{10}$
Figure S19. HPLC, ESI-MS, ^{1}H NMR,^{13}C NMR and Dept of Cbz-$TANHC_{12}$
Figure S20. HPLC, ESI-MS, ^{1}H NMR,^{13}C NMR and Dept of Cbz-$TANHC_{14}$
Figure S21. HPLC, ESI-MS, ^{1}H NMR,^{13}C NMR and Dept of $TANHC_{8}$
Figure S22. HPLC, ESI-MS, ^{1}H NMR,^{13}C NMR and Dept of $TANHC_{10}$
Figure S23. HPLC, ESI-MS, ^{1}H NMR,^{13}C NMR and Dept of $TANHC_{12}$
Figure S24. HPLC, ESI-MS, ^{1}H NMR,^{13}C NMR and Dept of $TANHC_{14}$
Figure S25. HPLC, ^{1}H NMR,^{13}C NMR and Dept of $C_{3}(TANHC_{8})_{2}$
Figure S26. Specific conductivity-concentration curves for the amino acid-based surfactants

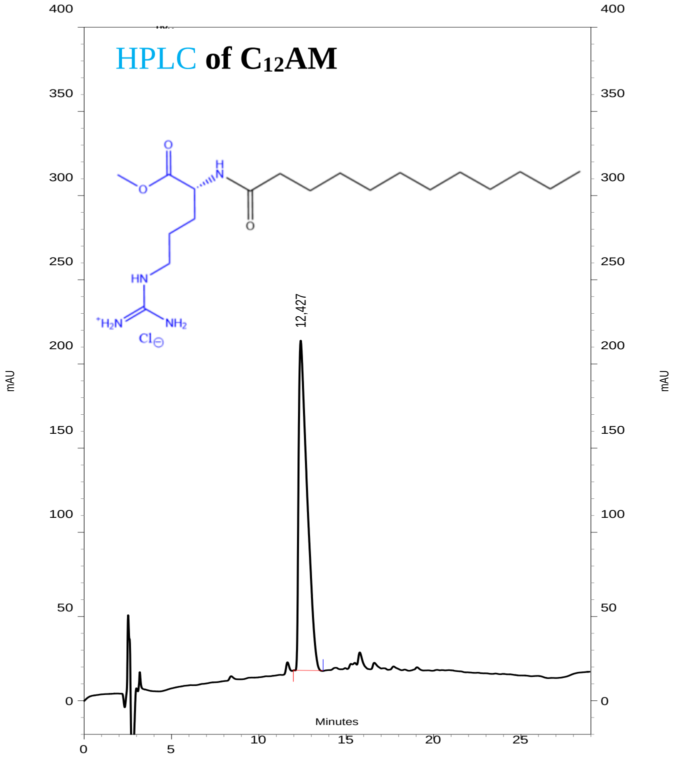

ESI-MS of C_{12}AM

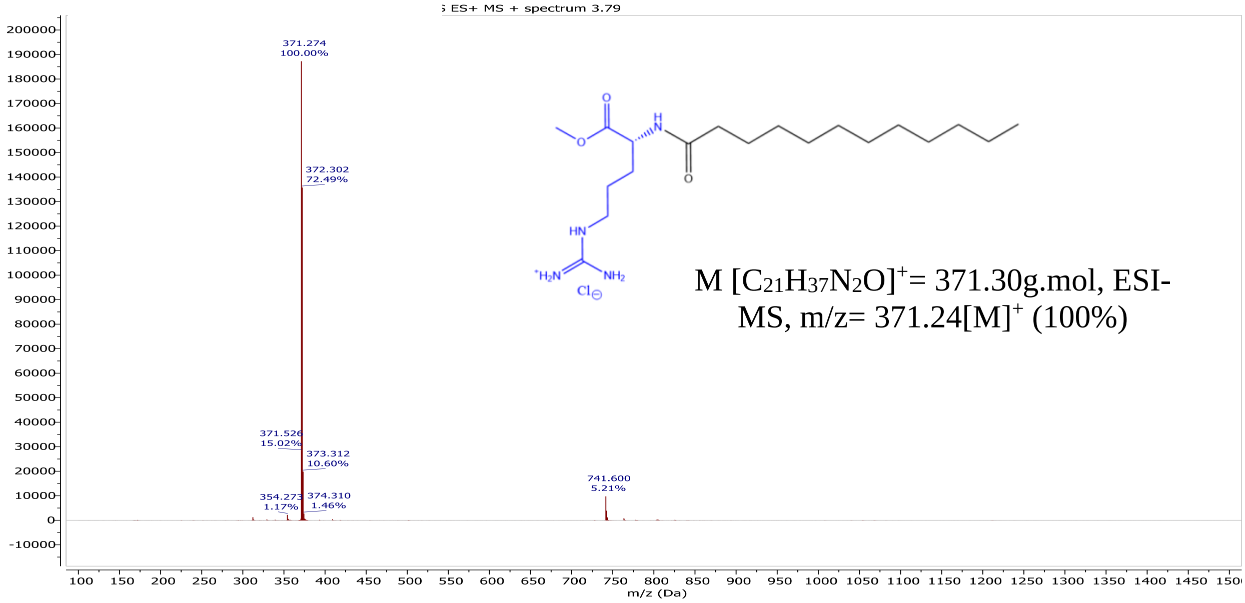

^{1}H NMR of C_{12}AM

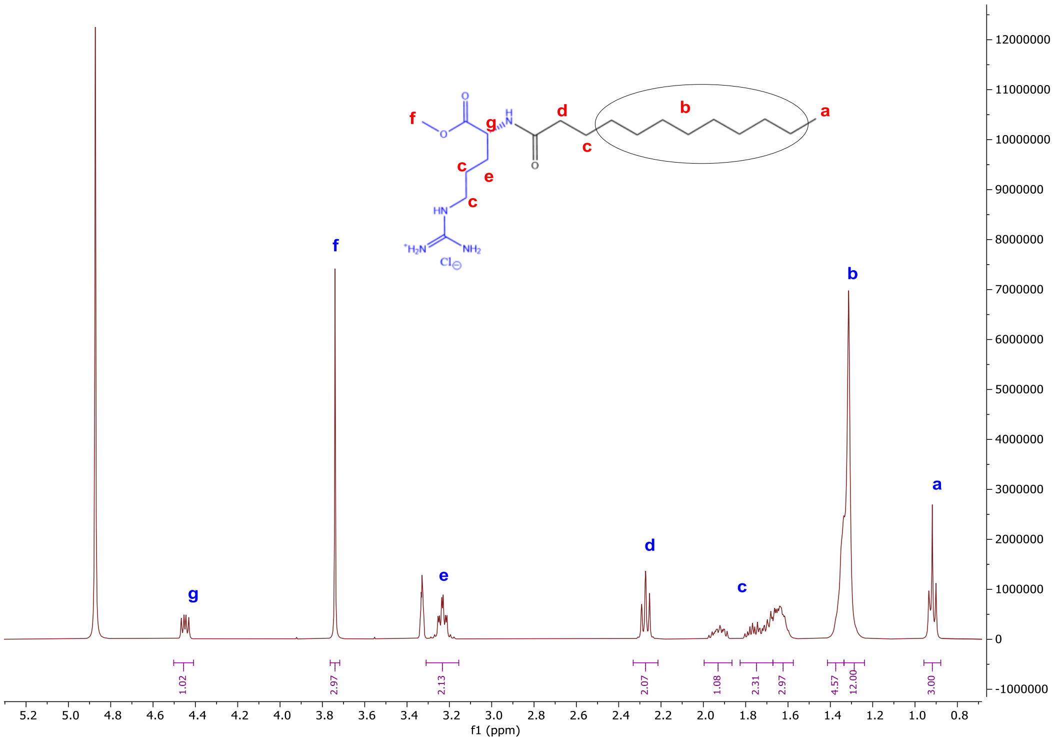

^{13}C NMR of C_{12}AM

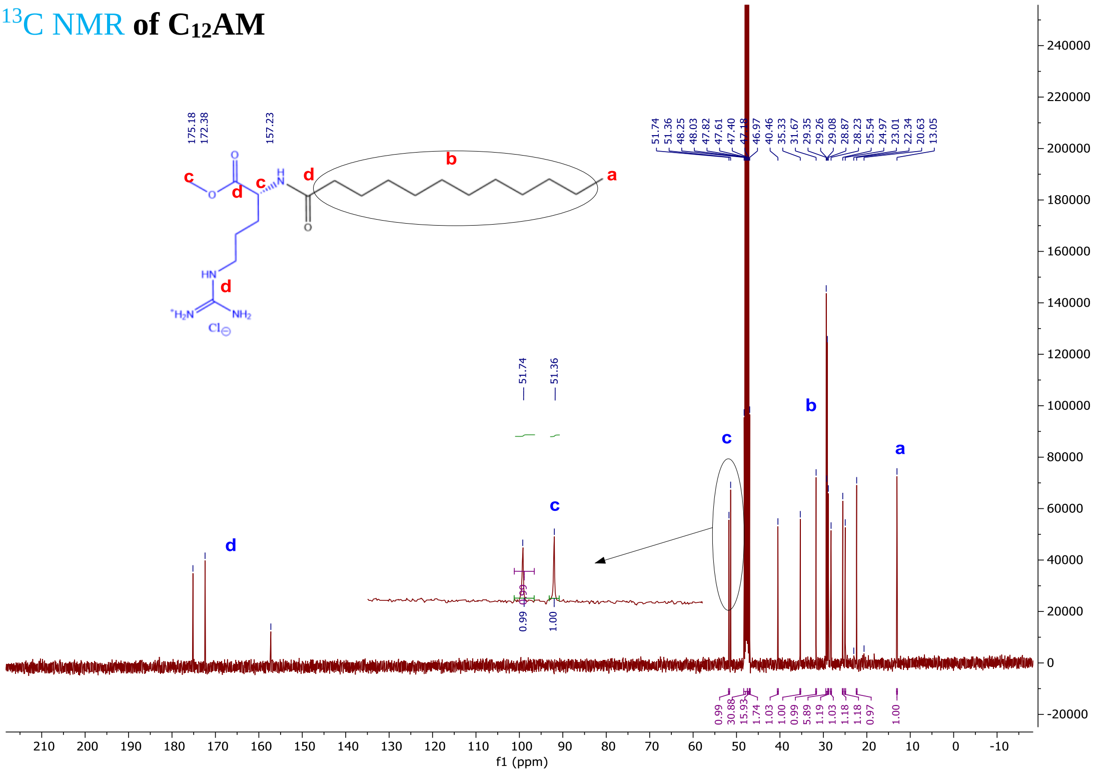

Figure S1. HPLC, ESI-MS, ^{1}H NMR, and ^{13}C NMR of C_{12}AM

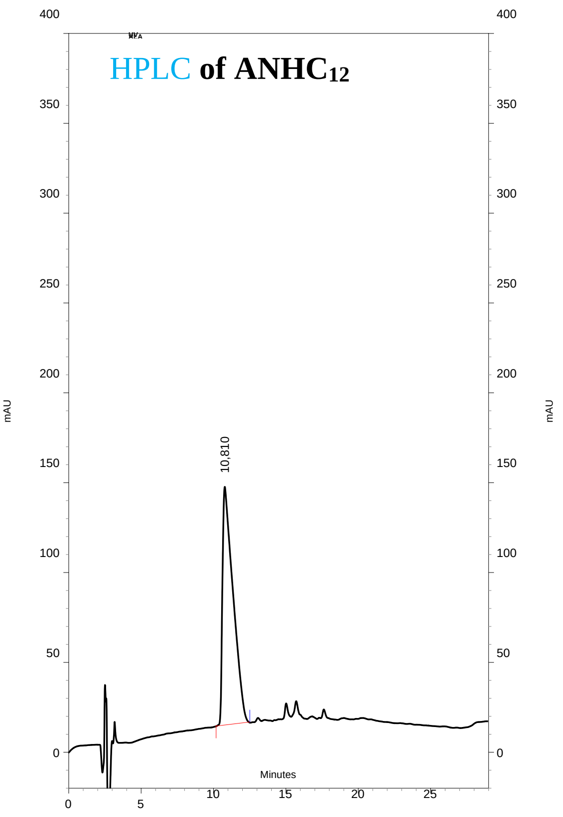

^{1}H NMR of ANHC$_{12}$

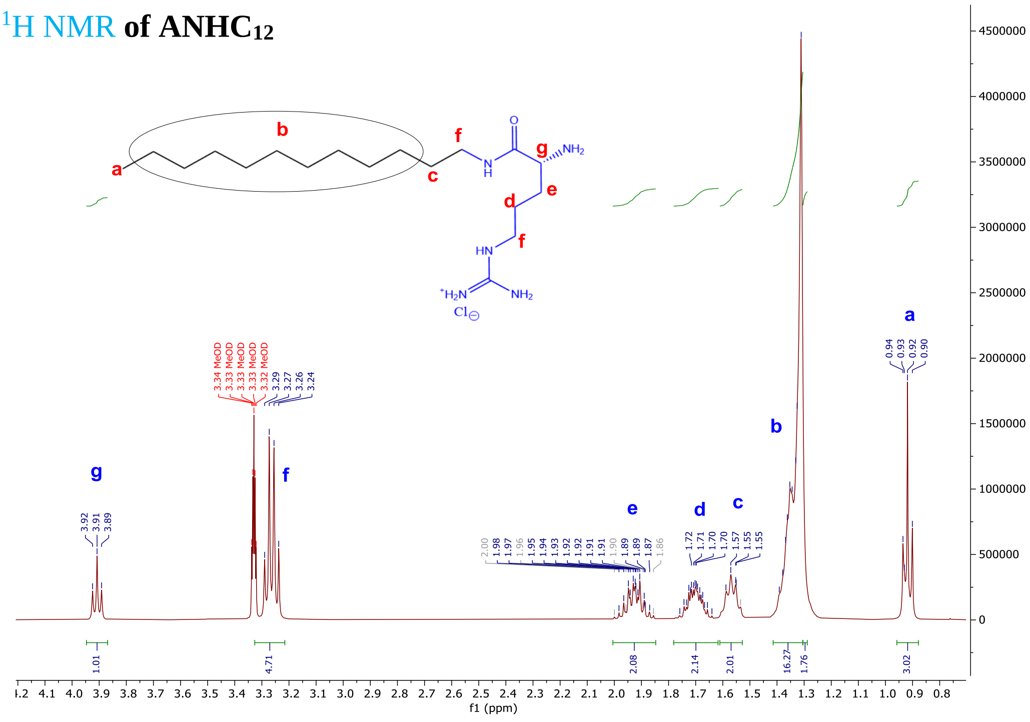

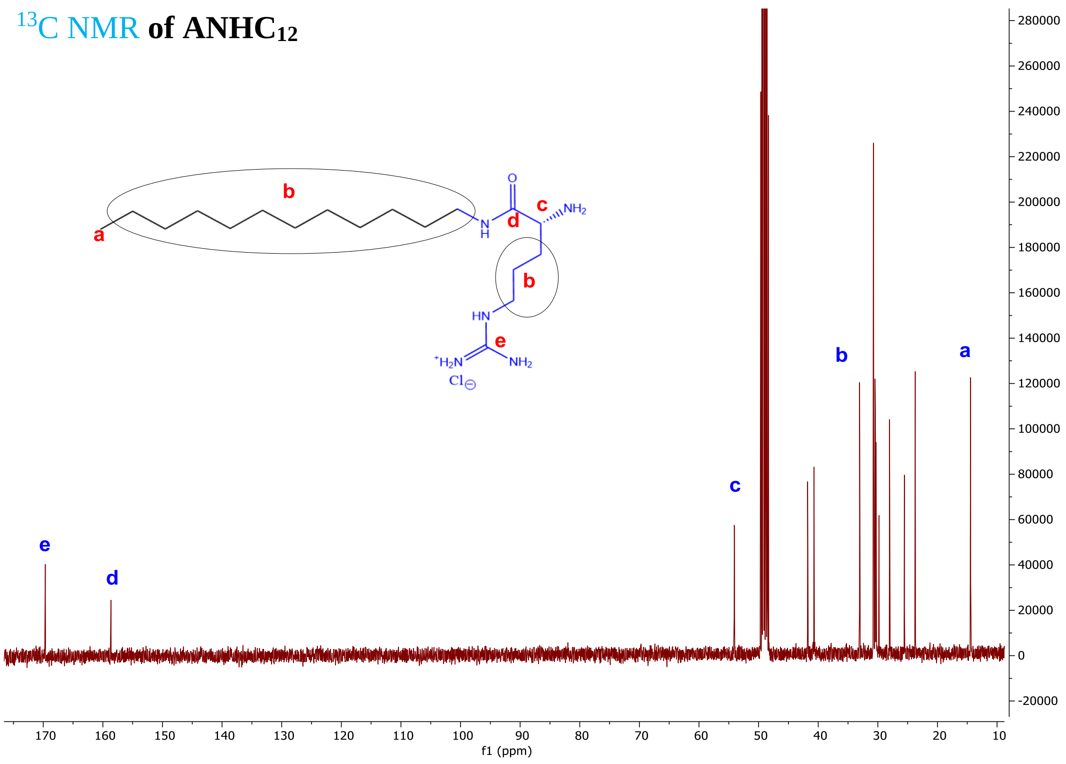

Figure S2. HPLC, ESI-MS, ^{1}H NMR, and ^{13}C NMR of $ANHC_{12}$

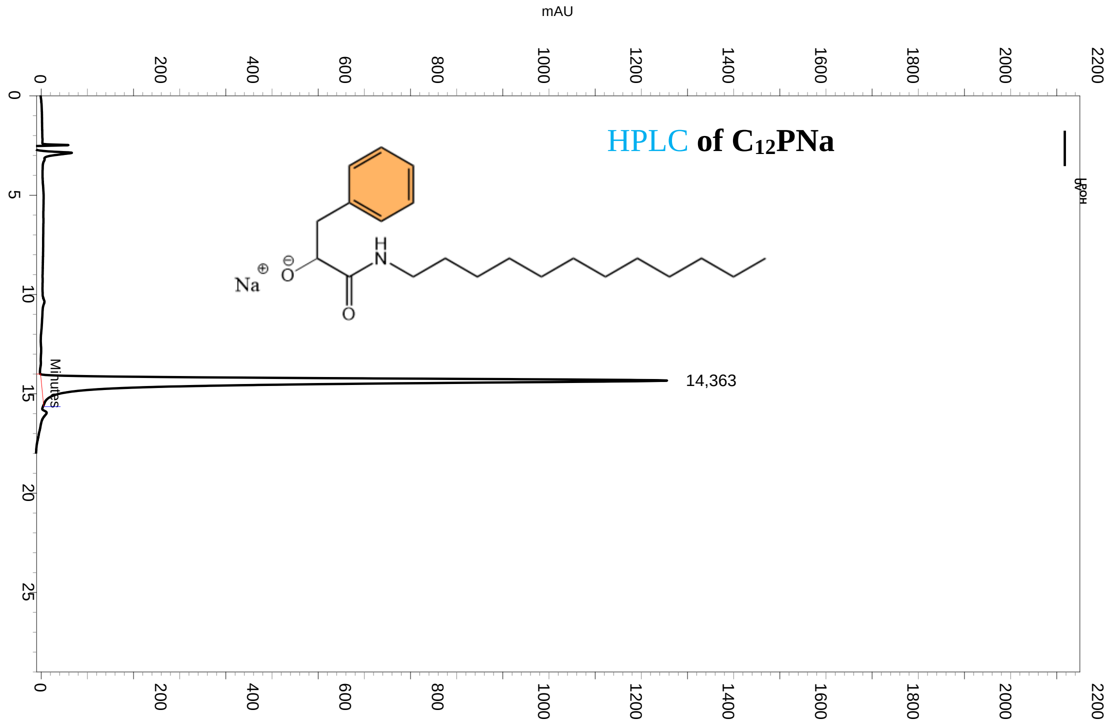

HPLC of C12PNa
mAU
Minutes
14,363
Na
O
H
N
O

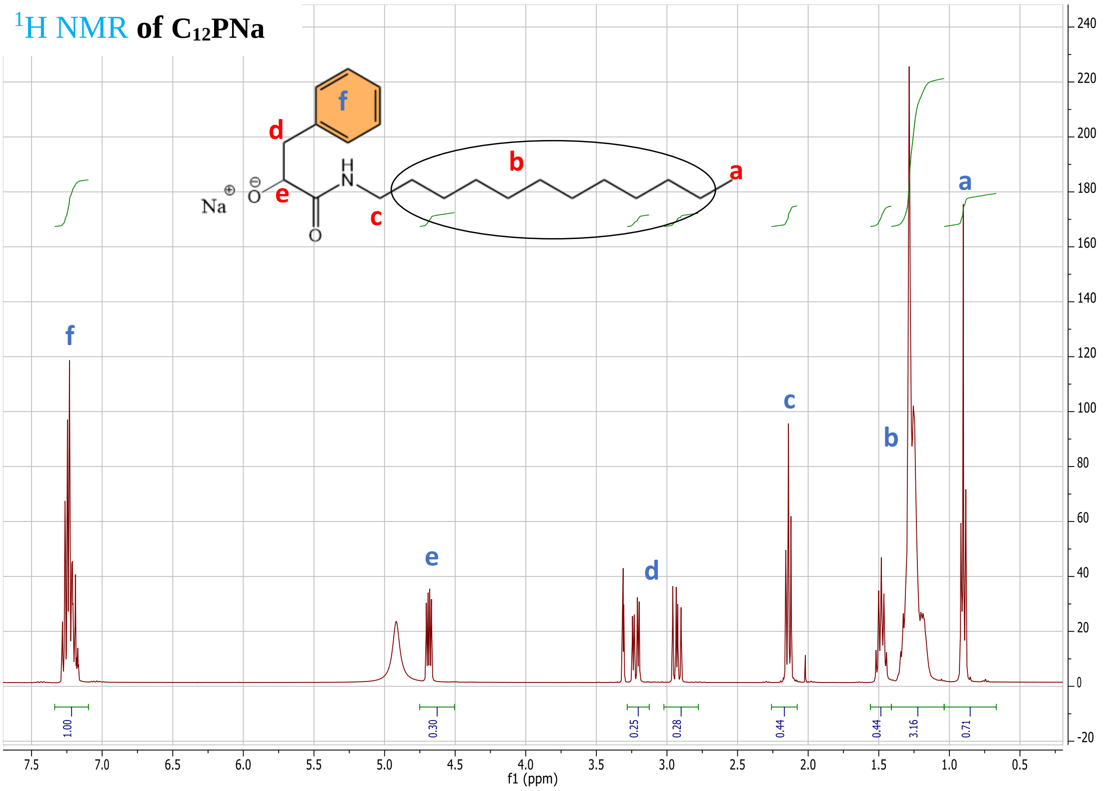

1H NMR of C12PNa
f
d
e
c
b
a
Na
O
H
N
O
1.00
0.30
0.25
0.28
0.44
0.44
3.16
0.71
f1 (ppm)

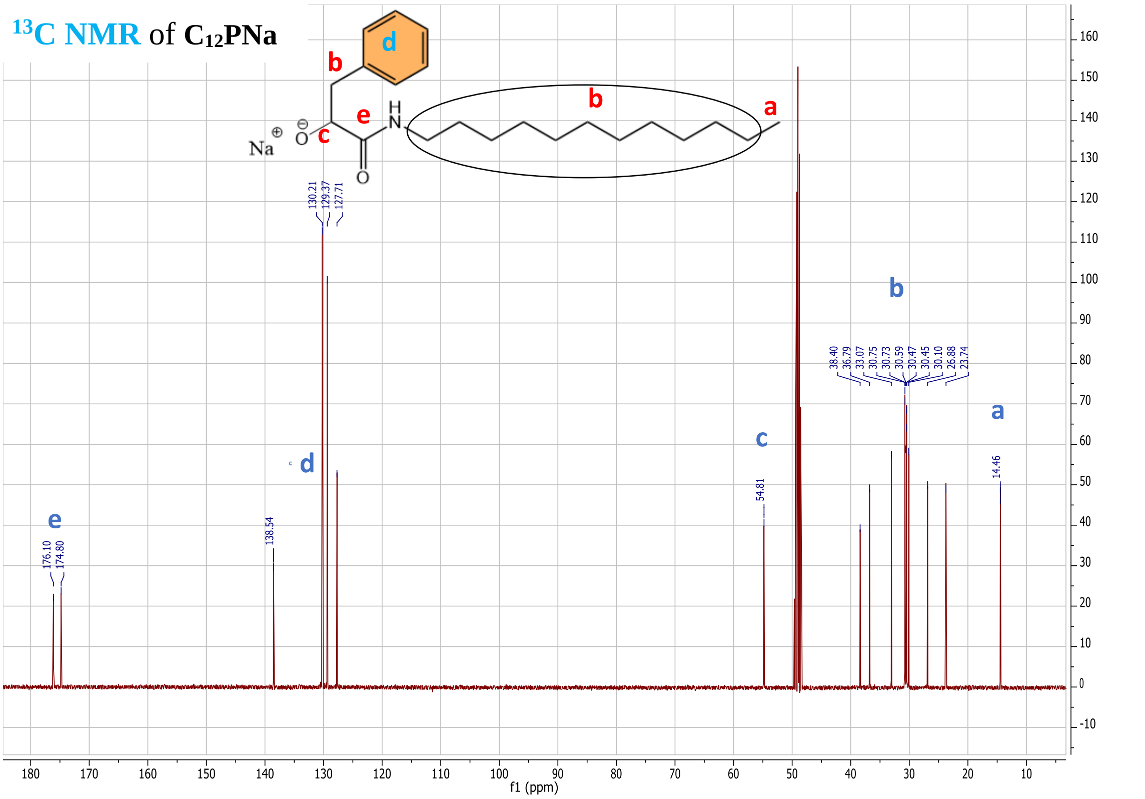

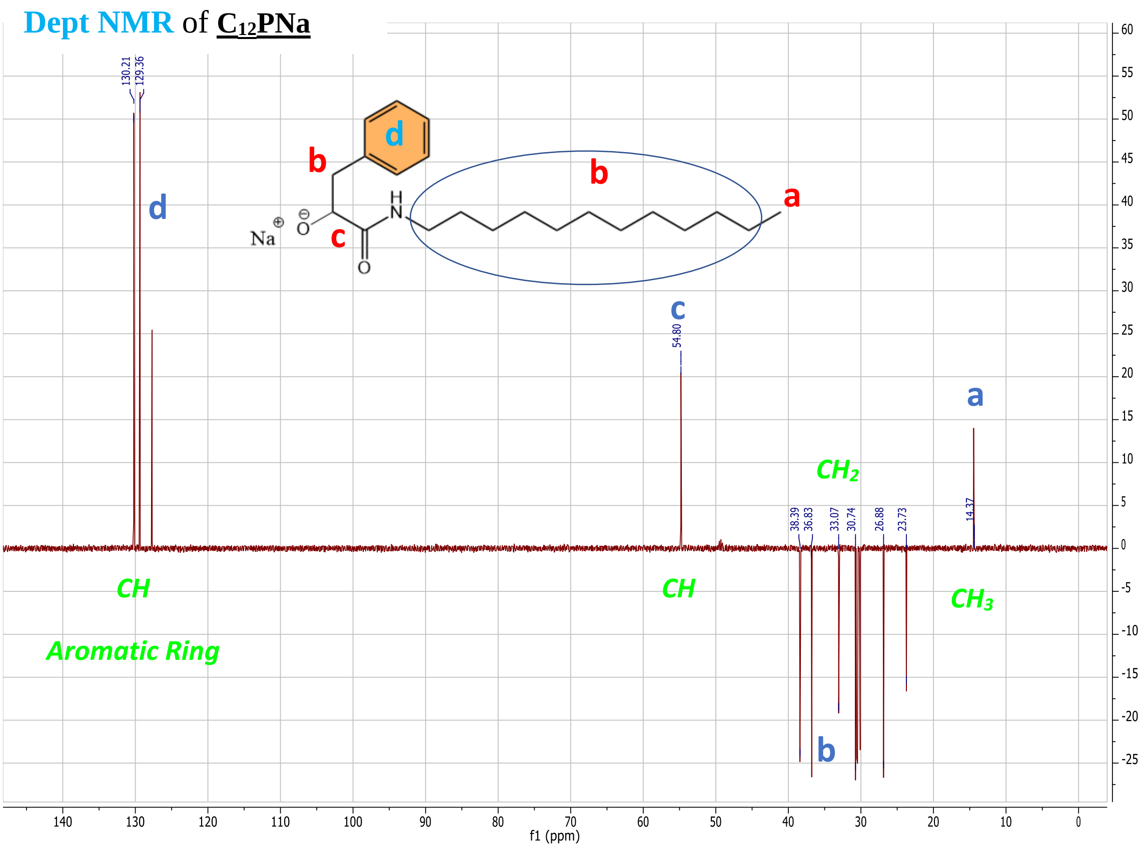

Figure S3. HPLC, ^{1}H NMR, ^{13}C NMR and Dept of $C_{12}PNa$

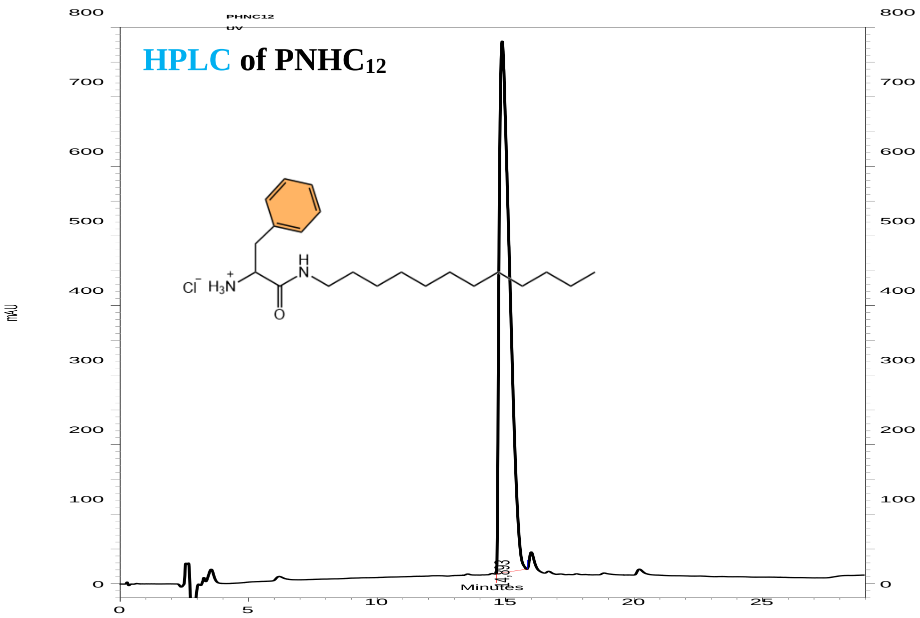
HPLC of PNHC12
PHNC12
UV
mAU
Minutes
14.333

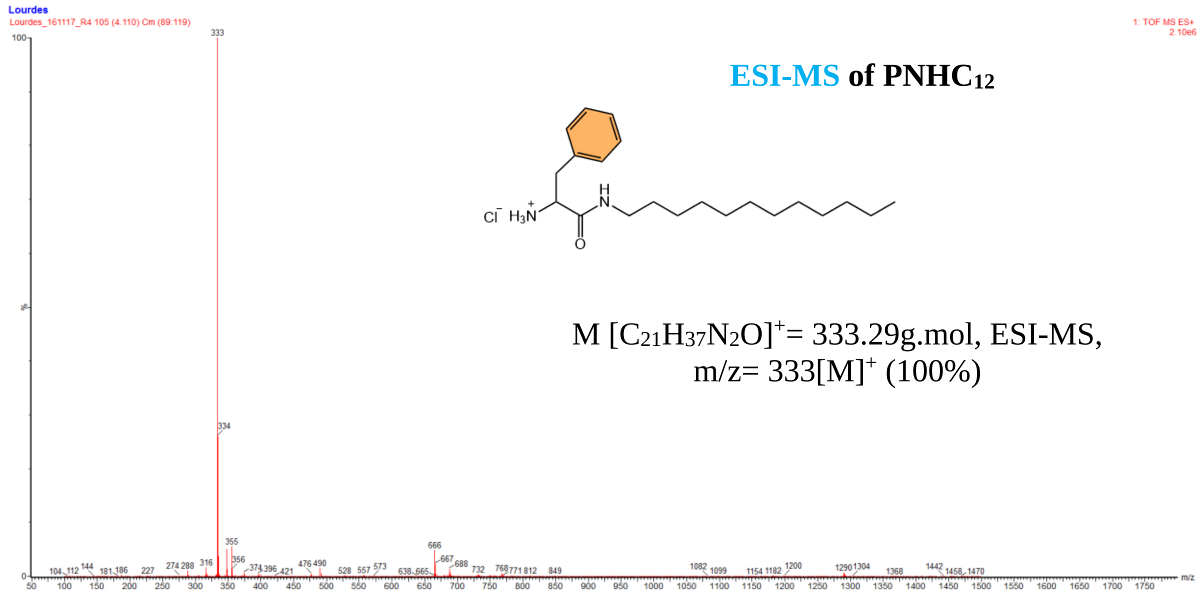
Lourdes
Lourdes_161117_R4 105 (4.110) Cm (89:119)
1: TOF MS ES+
2.10e6
ESI-MS of PNHC12
M [C21H37N2O]+= 333.29g.mol, ESI-MS,
m/z= 333[M]+ (100%)
m/z

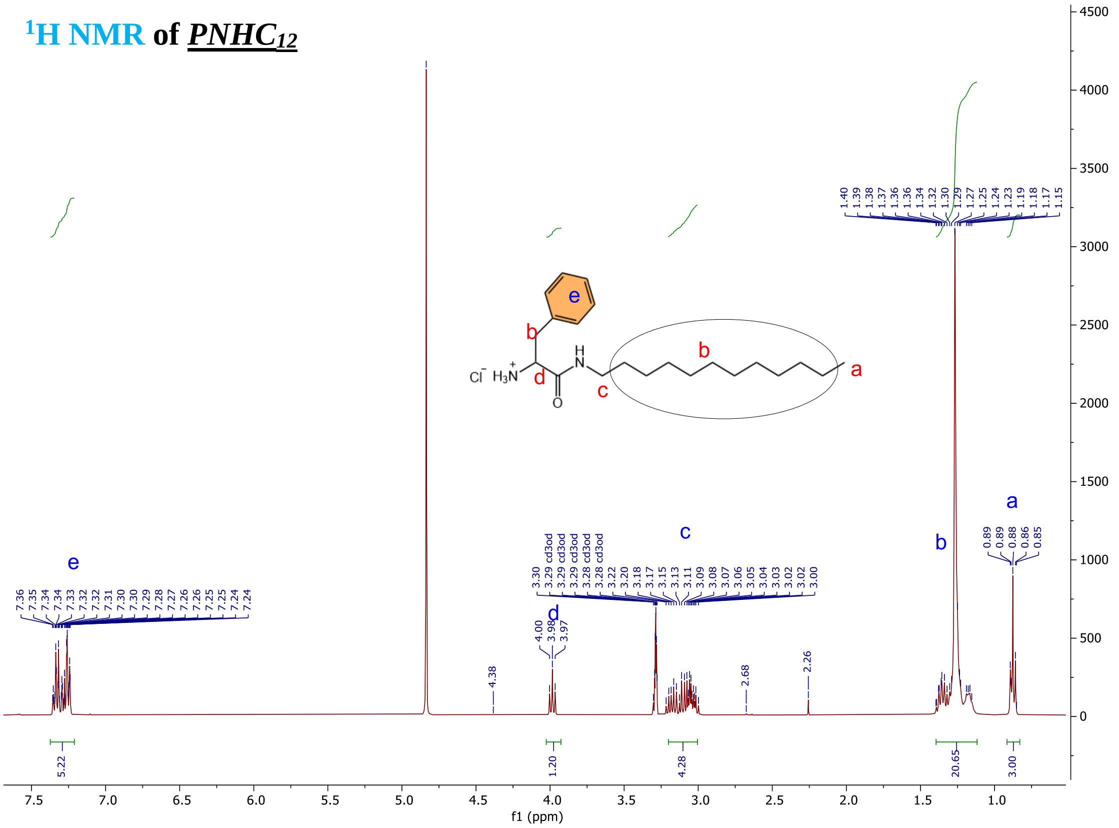

Figure S4. HPLC, ESI-MS and ^{1}H NMR, of $PNHC_{12}$

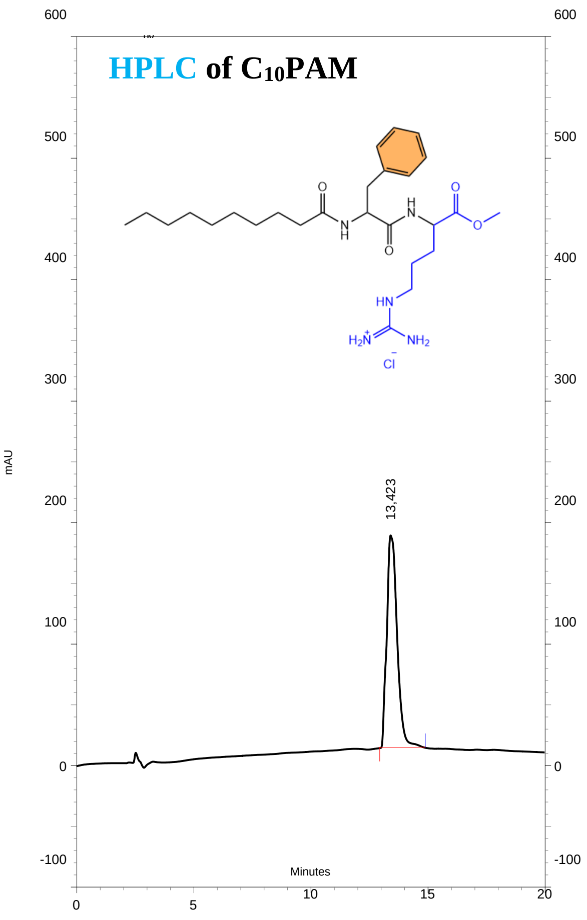

ESI-MS of C_{10}PAM

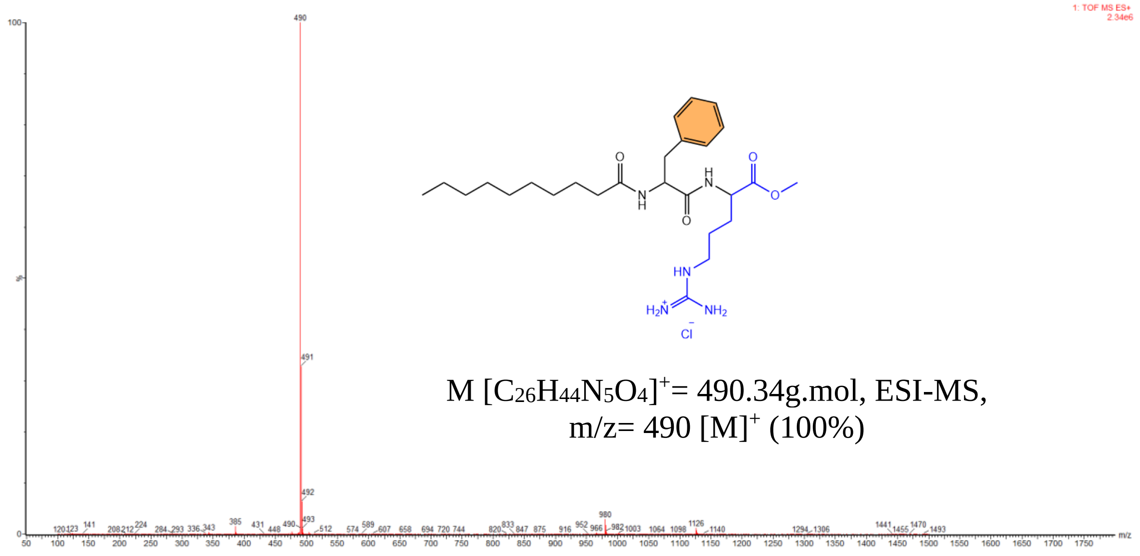

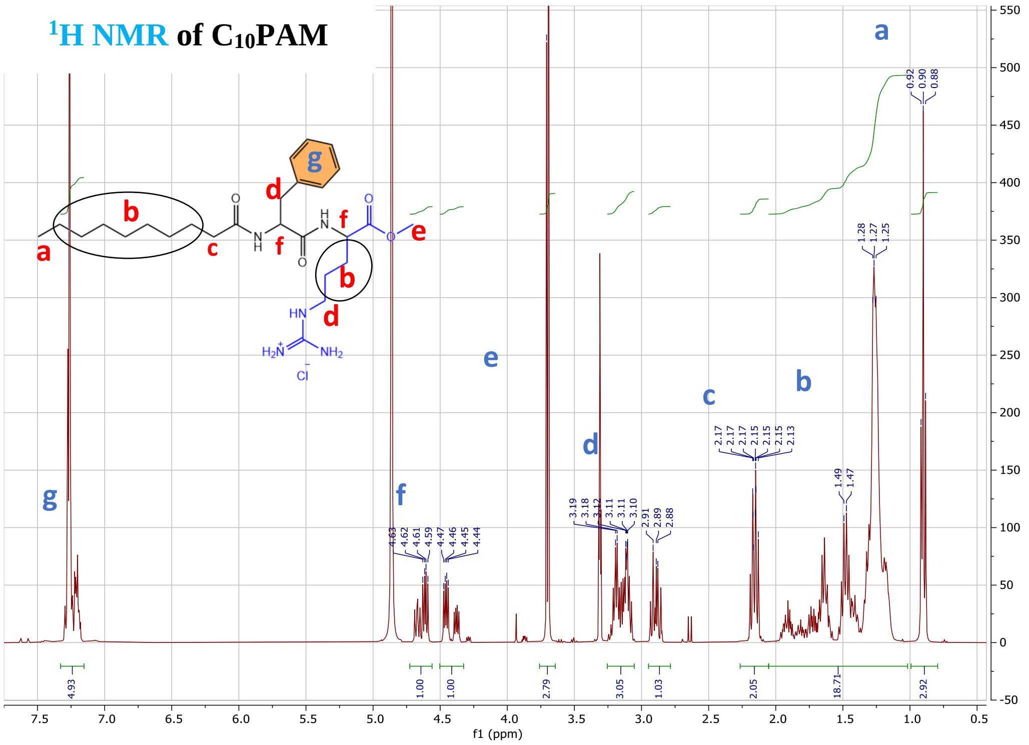
1H NMR of C10PAM
a
b
c
d
e
f
g
HN
H2N
NH2
Cl
0.92 0.90 0.88
1.28 1.27 1.25
1.49 1.47
2.17 2.17 2.17 2.15 2.15 2.15 2.13
3.19 3.18 3.12 3.11 3.11 3.10 2.91 2.89 2.88
4.63 4.62 4.61 4.59 4.47 4.46 4.45 4.44
4.93
1.00
1.00
2.79
3.05
1.03
2.05
18.71
2.92
f1 (ppm)

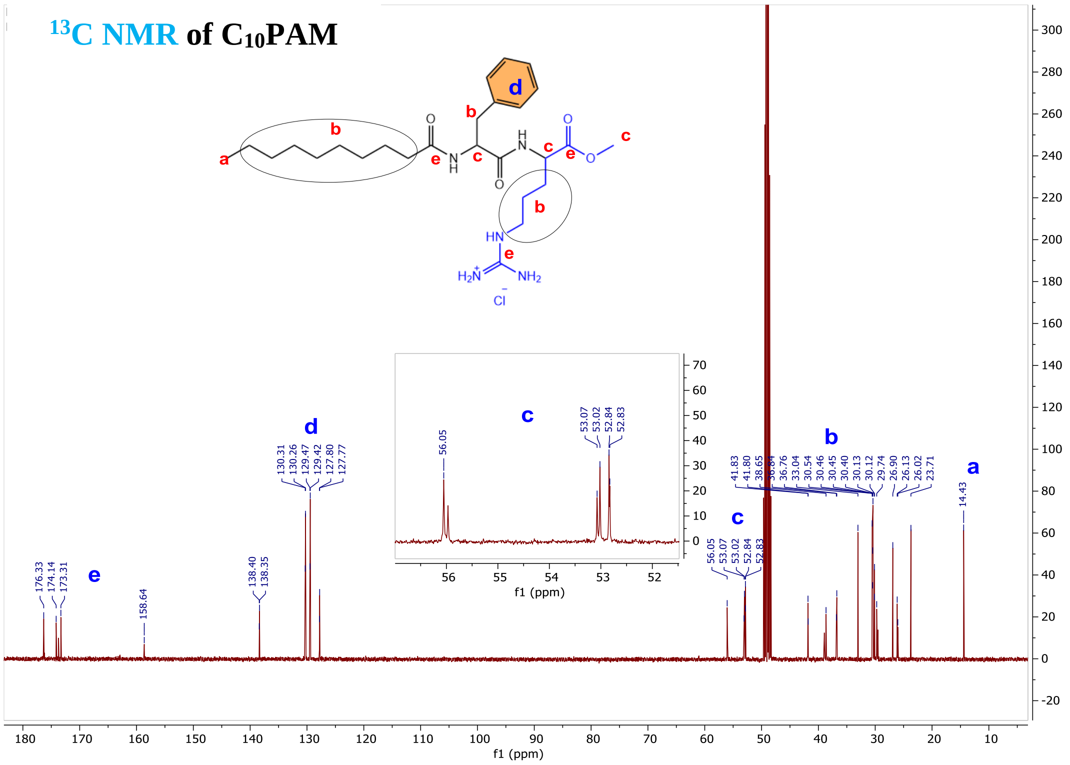
13C NMR of C10PAM
a
b
c
d
e
HN
H2N
NH2
Cl
176.33 174.14 173.31
158.64
138.40 138.35
130.31 130.26 129.47 129.42 127.80 127.77
56.05 53.07 53.02 52.84 52.83
41.83 41.80 38.65 38.64 36.76 33.04 30.54 30.46 30.45 30.40 30.13 30.12 29.74 26.90 26.13 26.02 23.71
14.43
56.05
53.07 53.02 52.84 52.83
f1 (ppm)

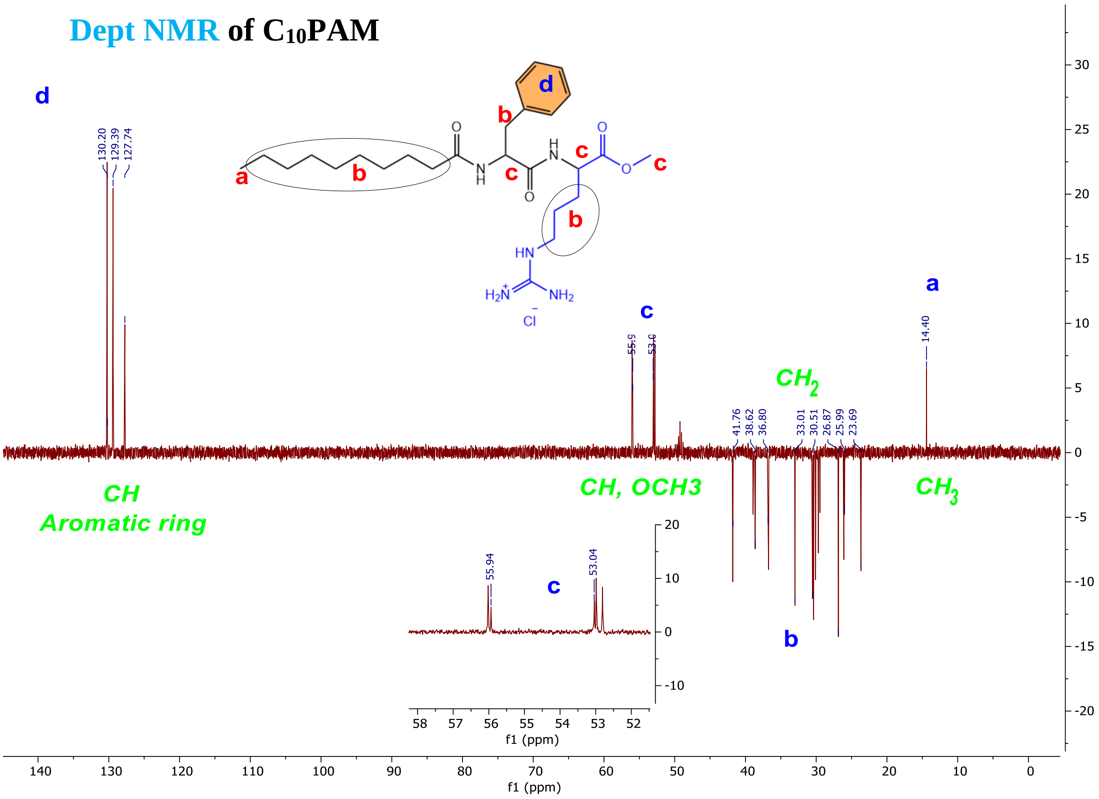

Figure S5. HPLC, ESI-MS, ^{1}H NMR, ^{13}C NMR and Dept of C_{10}PAM

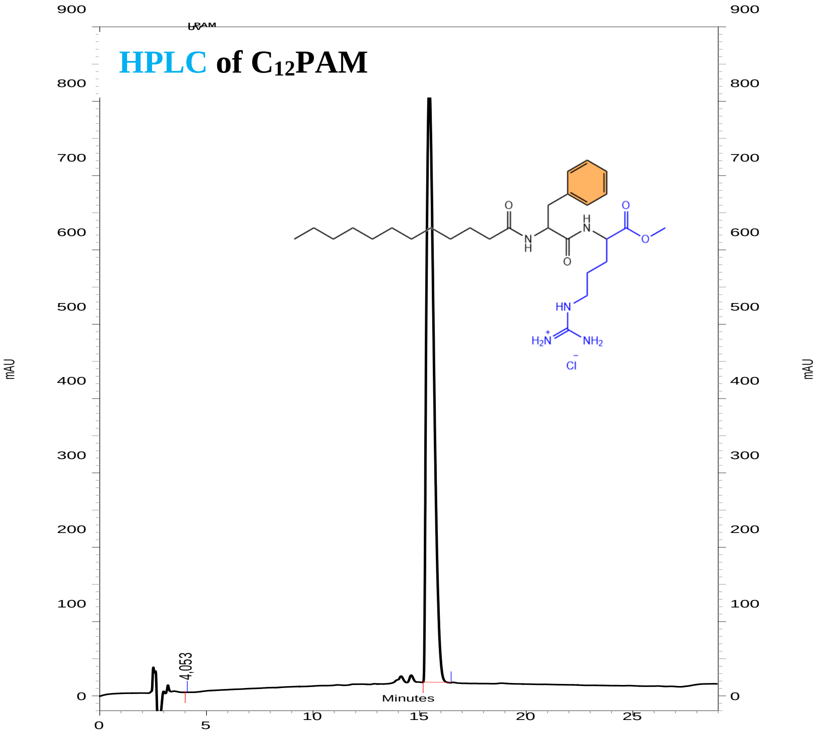
HPLC of C12PAM
mAU
Minutes
4.053
O
N
H
HN
H2N
NH2
Cl

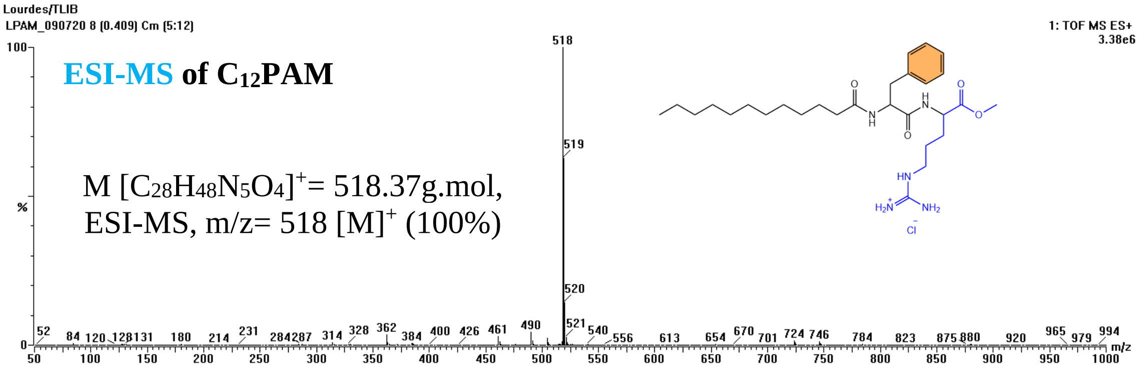
Lourdes/TLIB
LPAM_090720 8 (0.409) Cm (5:12)
1: TOF MS ES+
3.38e6
ESI-MS of C12PAM
M [C28H48N5O4]+= 518.37g.mol,
ESI-MS, m/z= 518 [M]+ (100%)
%
m/z
518
519
520
521
O
N
H
HN
H2N
NH2
Cl

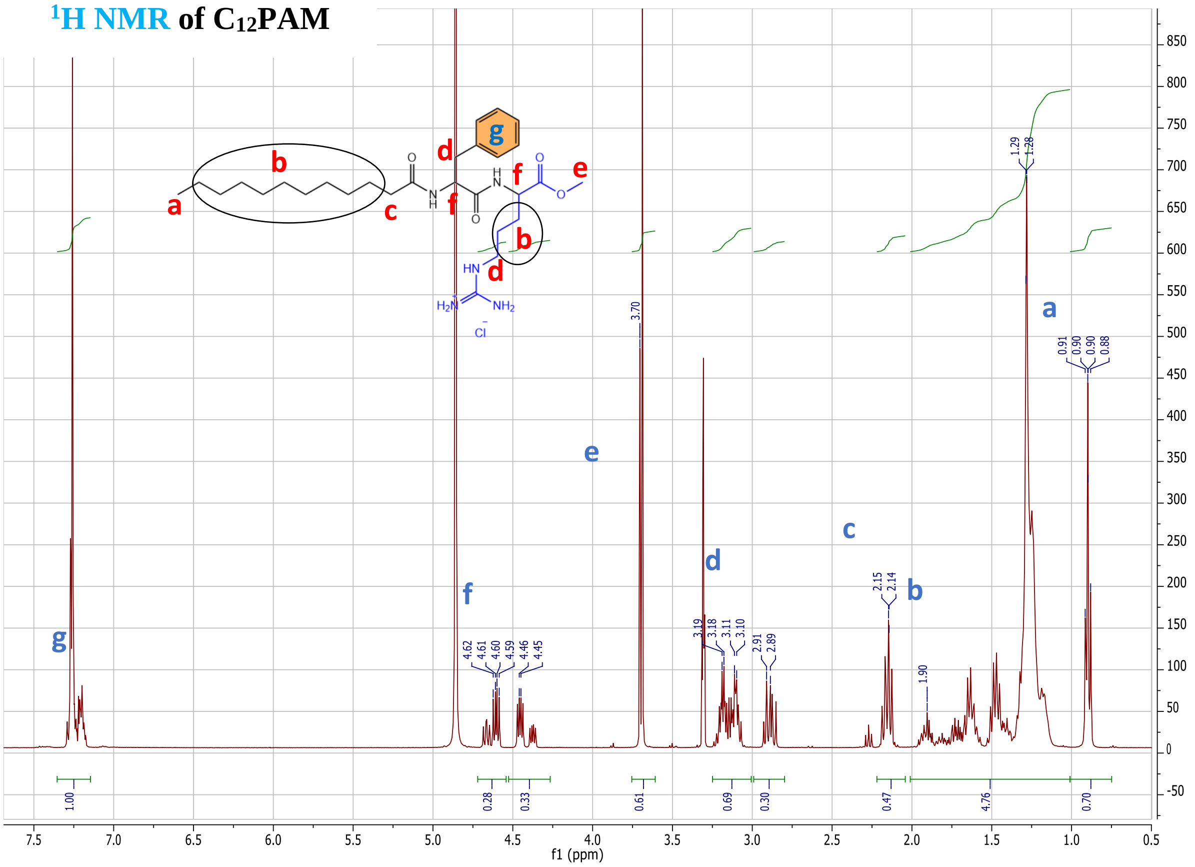
1H NMR of C12PAM
f1 (ppm)

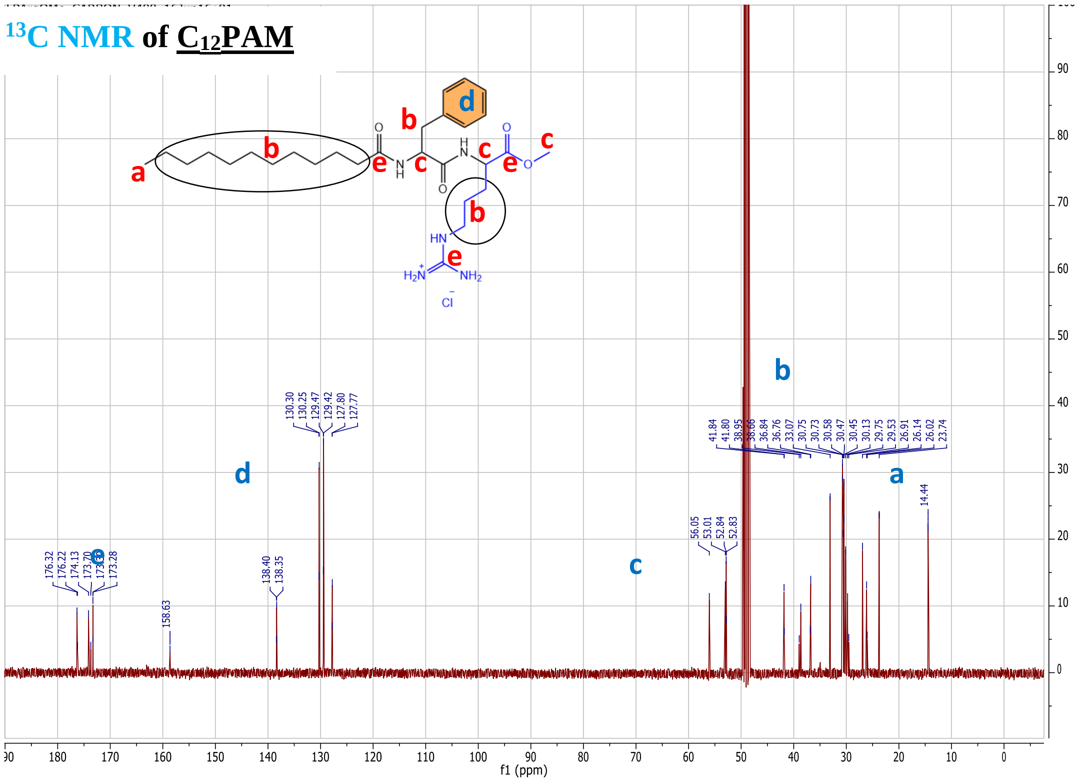
13C NMR of C12PAM
f1 (ppm)

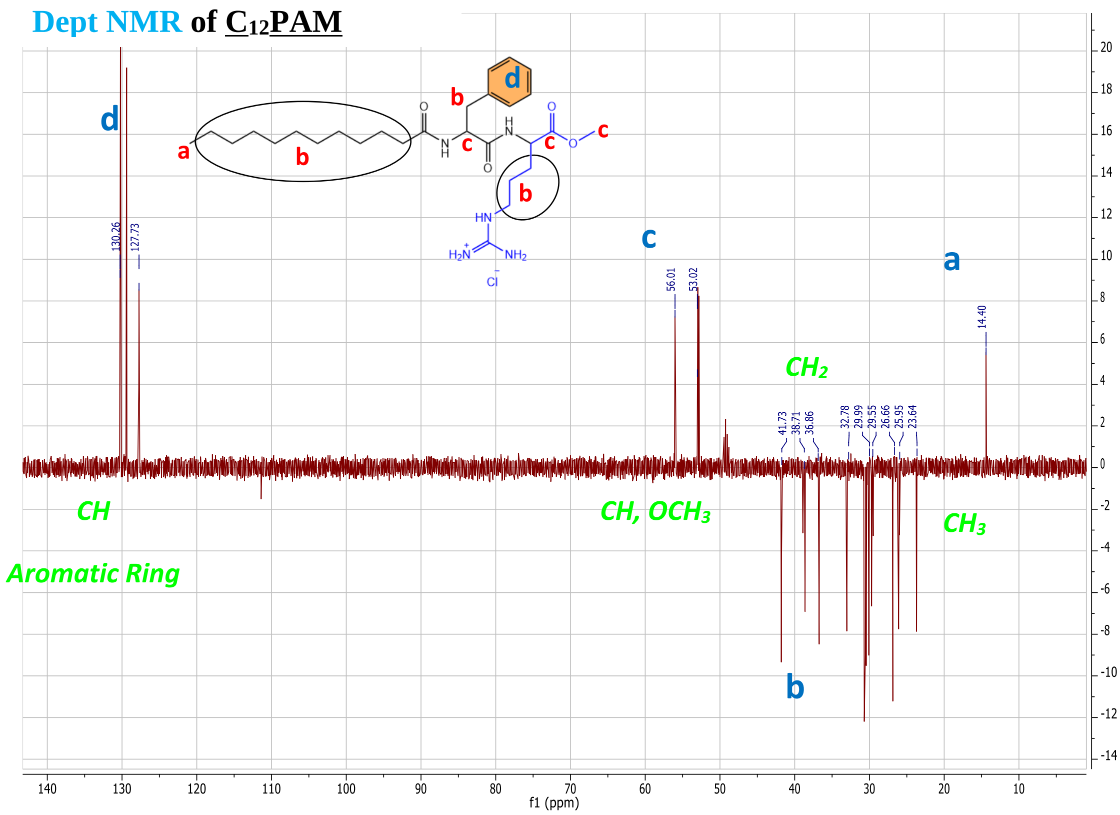

Figure S6. HPLC, ESI-MS, ^{1}H NMR, ^{13}C NMR and Dept of C_{12}PAM

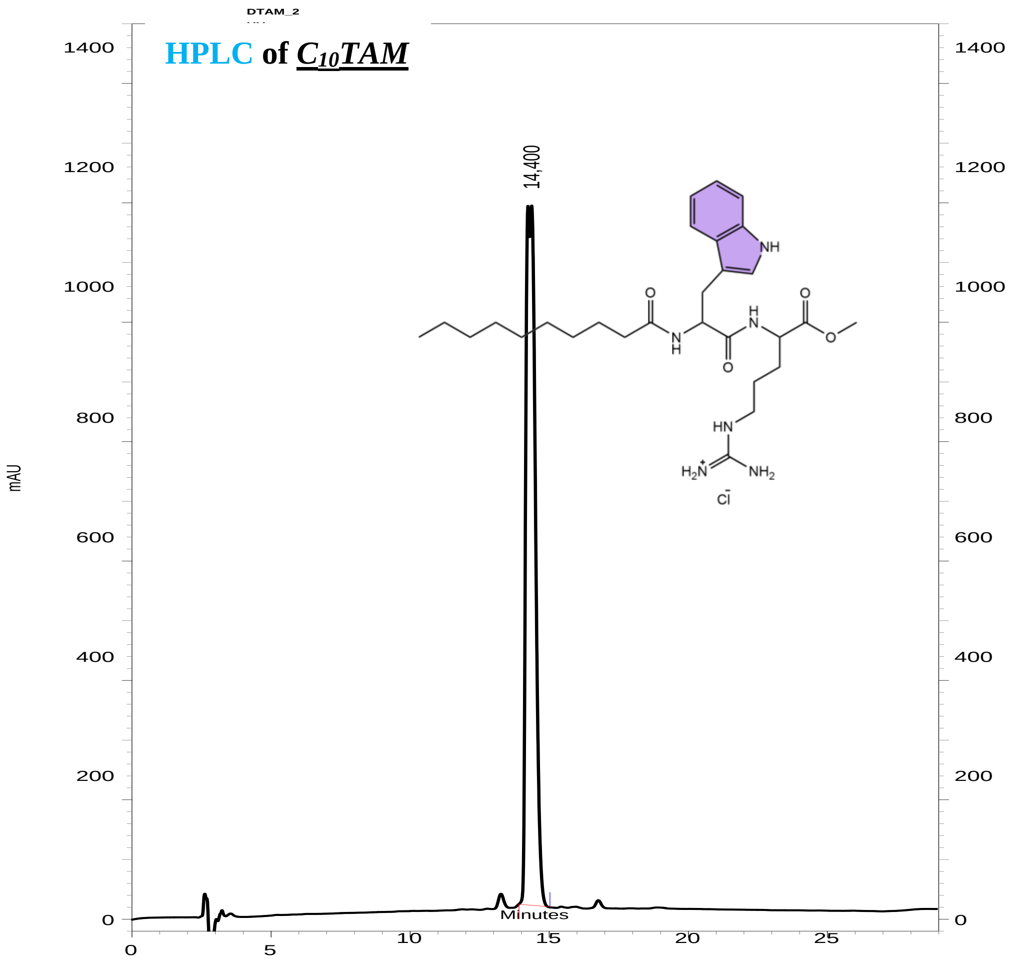

HPLC of C10TAM
14,400
mAU
Minutes

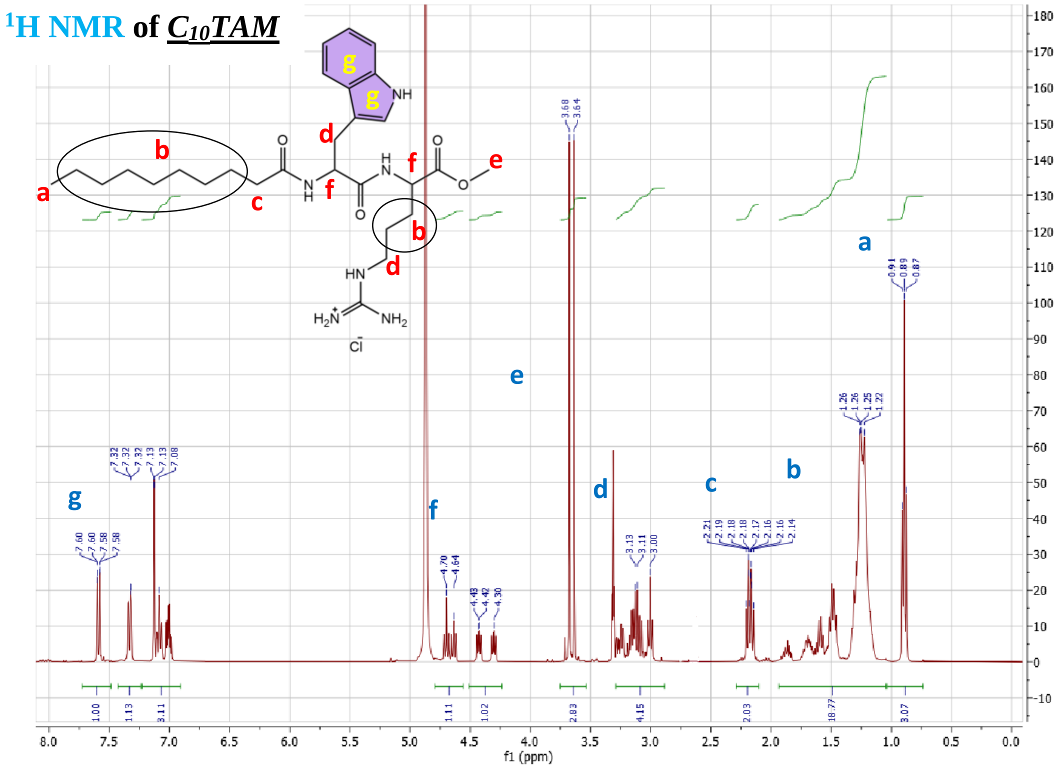

1H NMR of C10TAM
f1 (ppm)

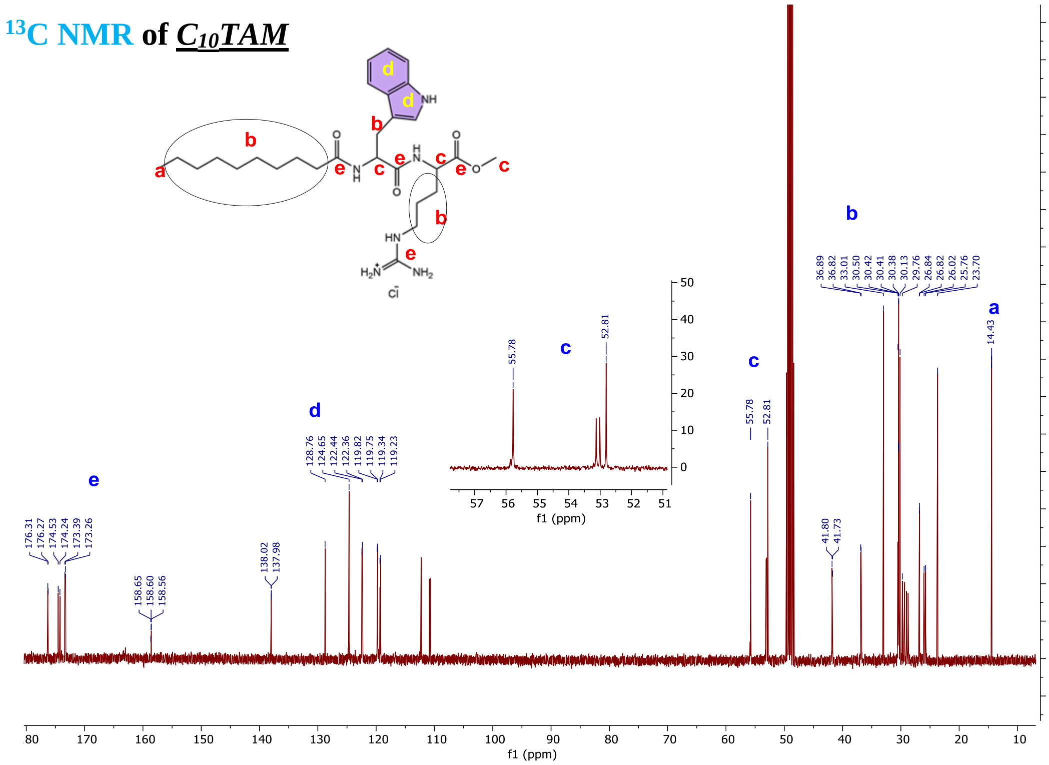

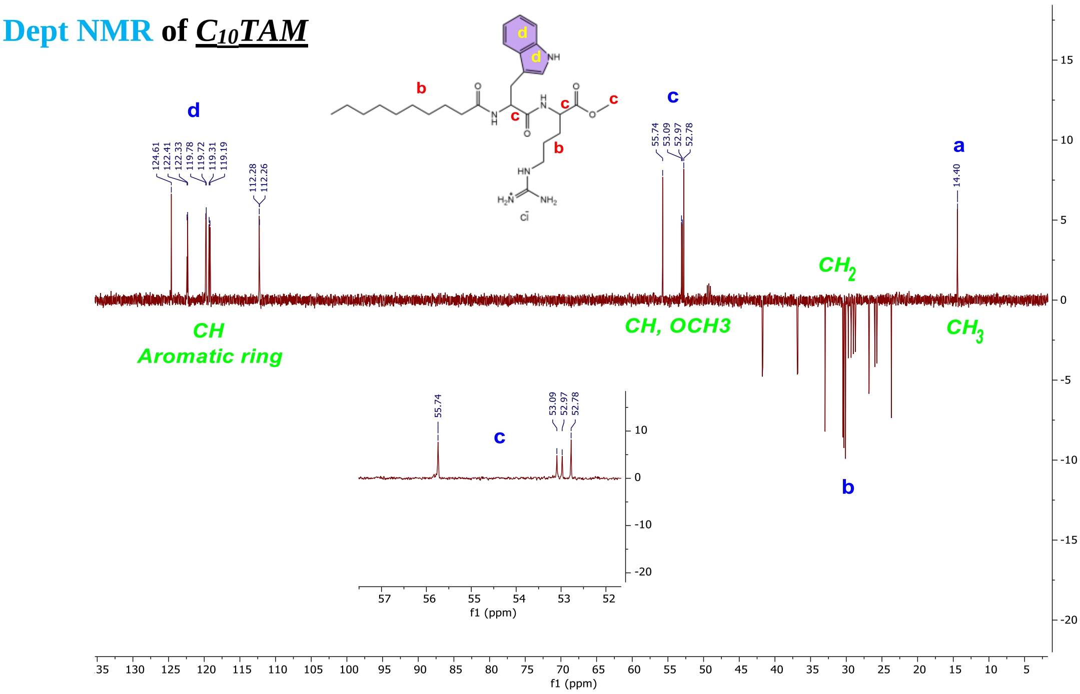

Figure S7. HPLC,^{1}H NMR,^{13}C NMR and Dept of C_{10}TAM

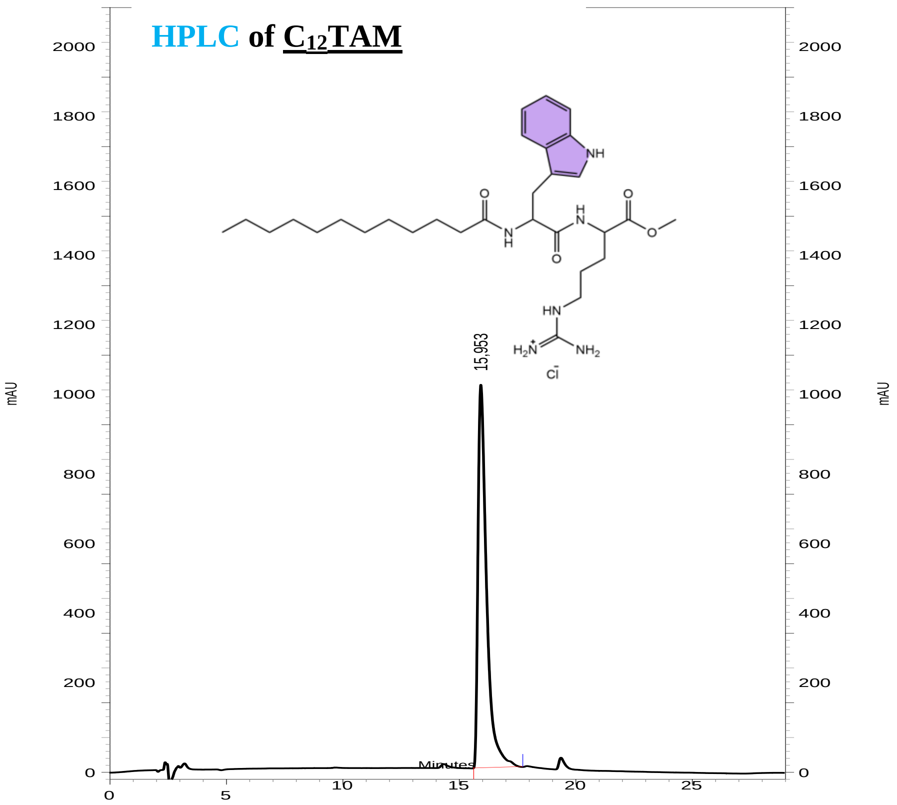
HPLC of C_{12}TAM
mAU
15,953
Minutes
NH
HN
H_2N^+
NH_2
Cl^-

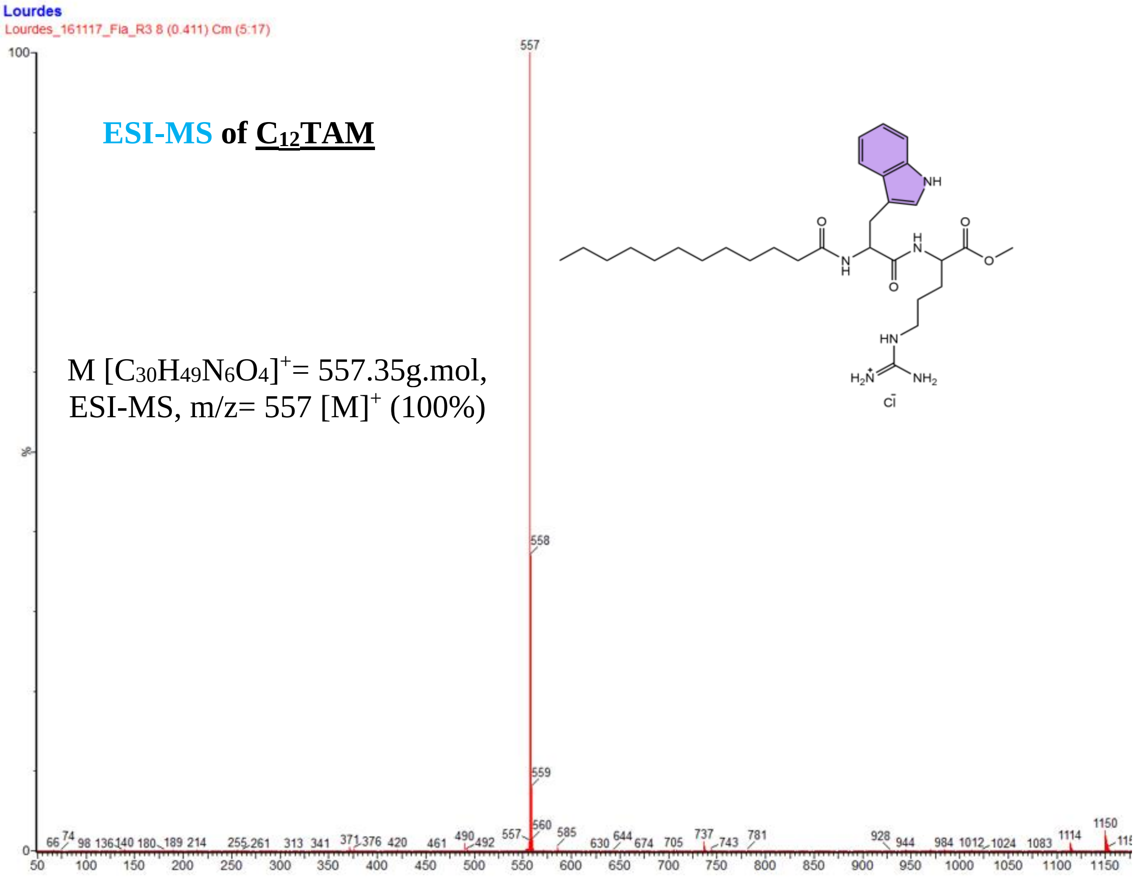
Lourdes
Lourdes_161117_Fia_R3 8 (0.411) Cm (5:17)
ESI-MS of C_{12}TAM
M $[C_{30}H_{49}N_6O_4]^+$= 557.35g.mol,
ESI-MS, m/z= 557 $[M]^+$ (100%)
557
558
559
560
%

^{1}H NMR of C_{12}TAM

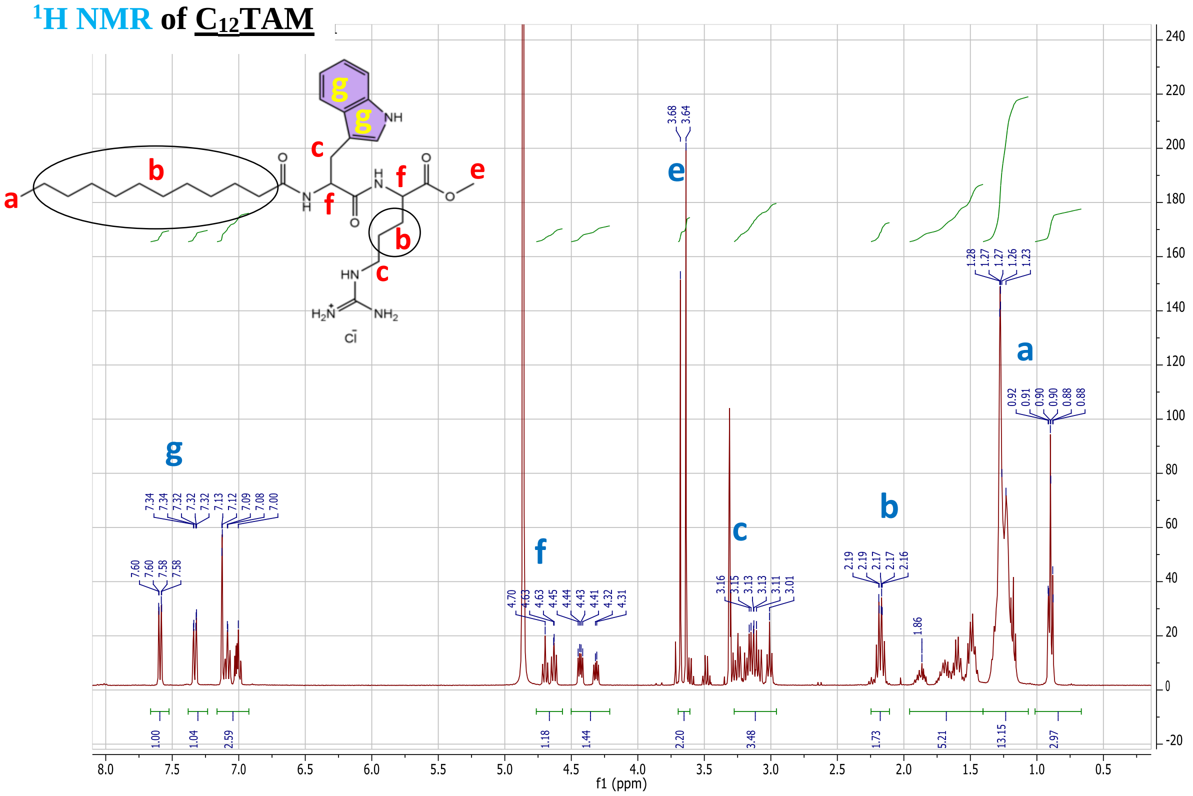

^{13}C NMR of C_{12}TAM

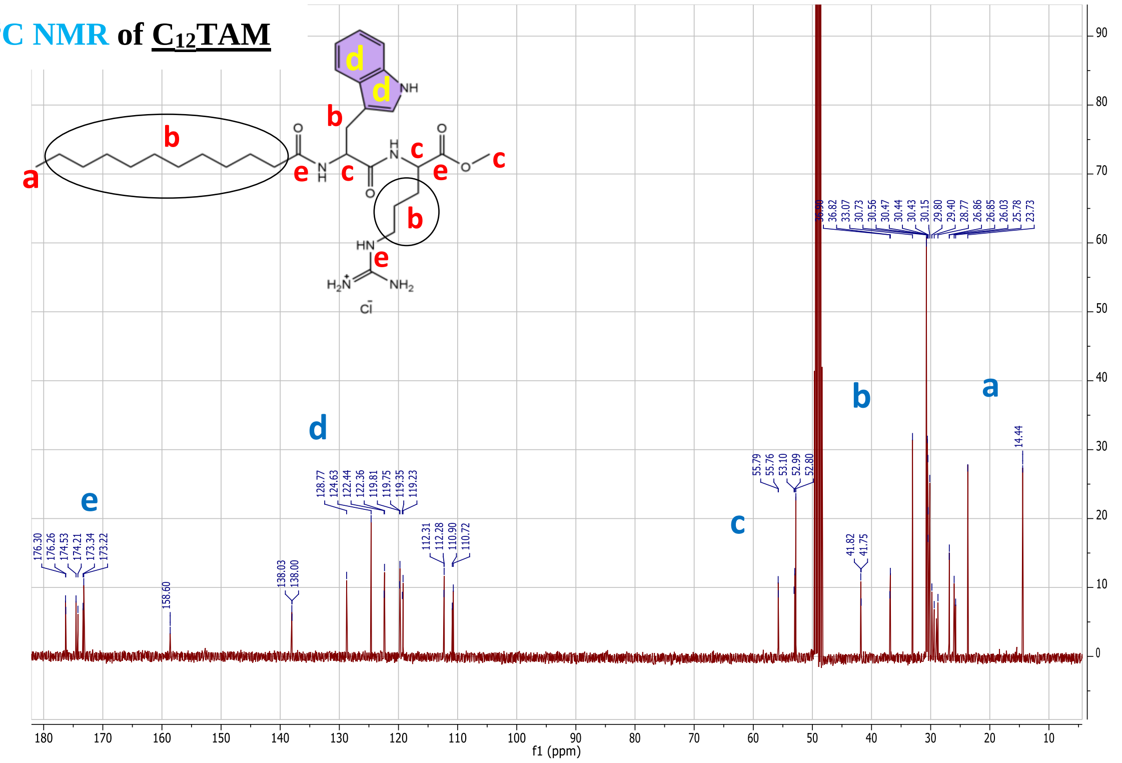

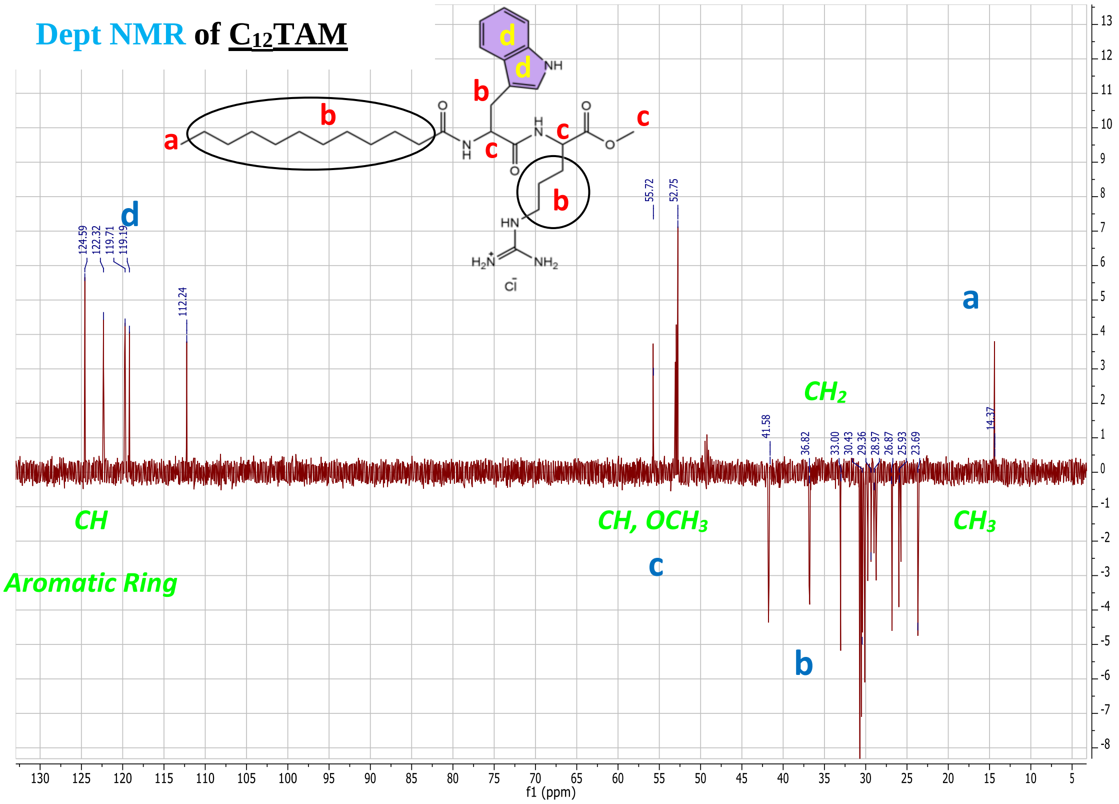

Figure S8. HPLC, ESI-MS, ^{1}H NMR, ^{13}C NMR and Dept of $C_{10}TAM$

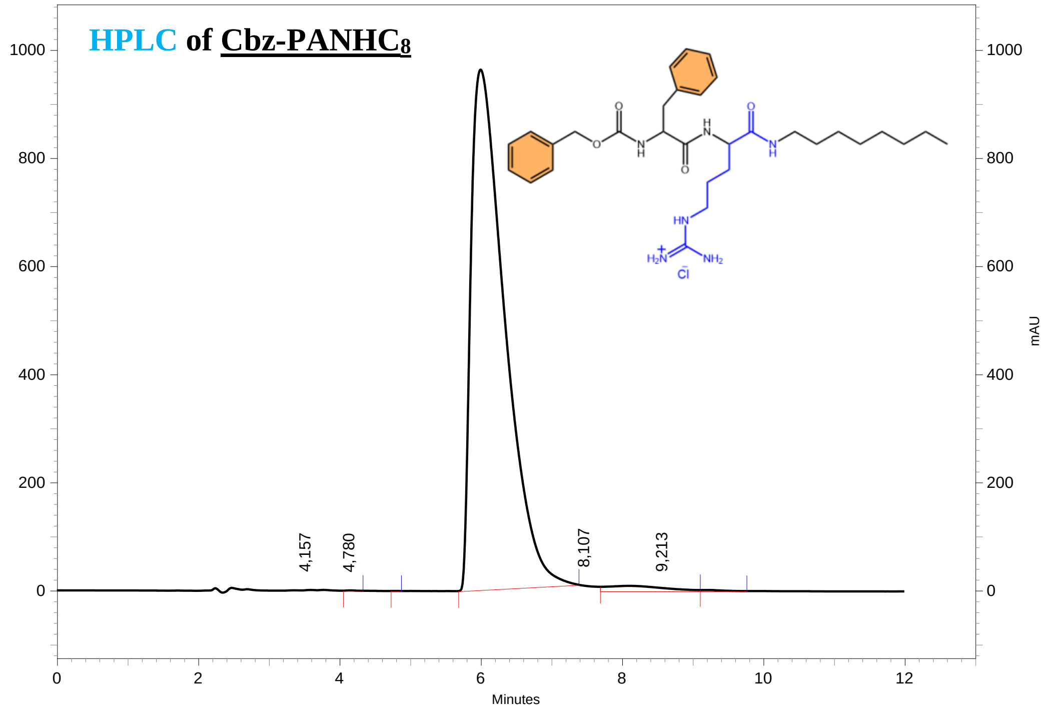

ESI-MS of Cbz-PANHC8

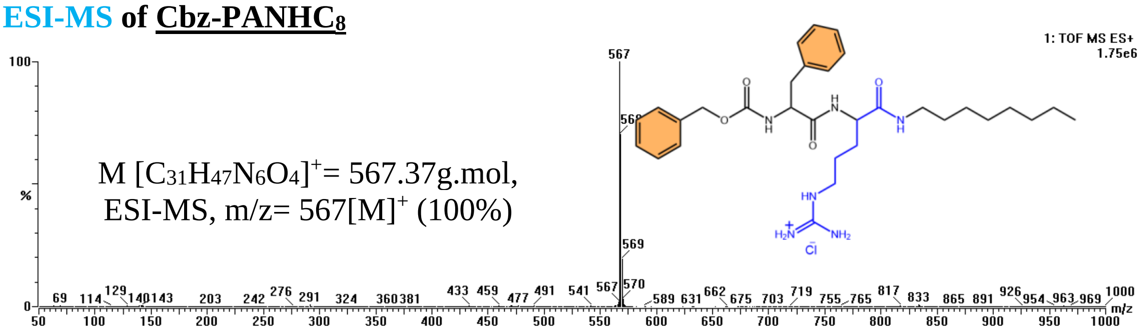

^{1}H NMR of Cbz-PANHC8

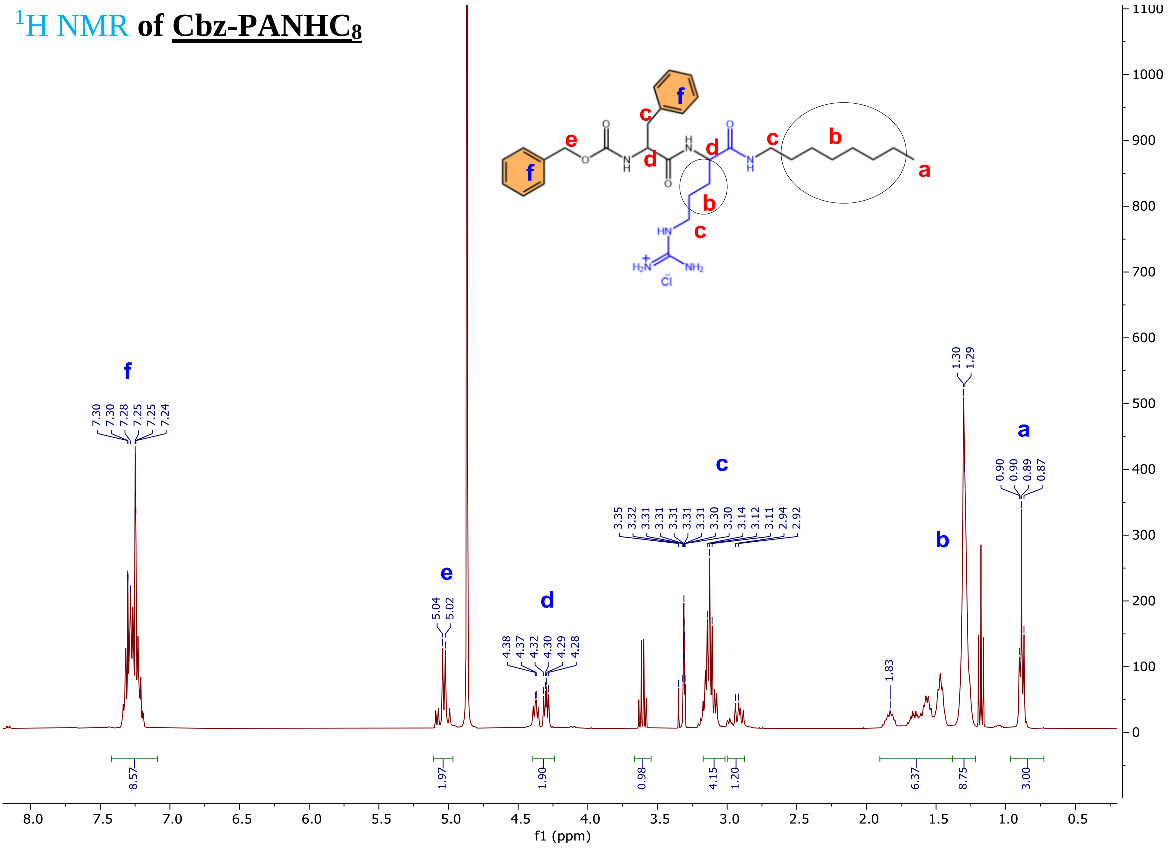

^{13}C NMR of Cbz-PANC8

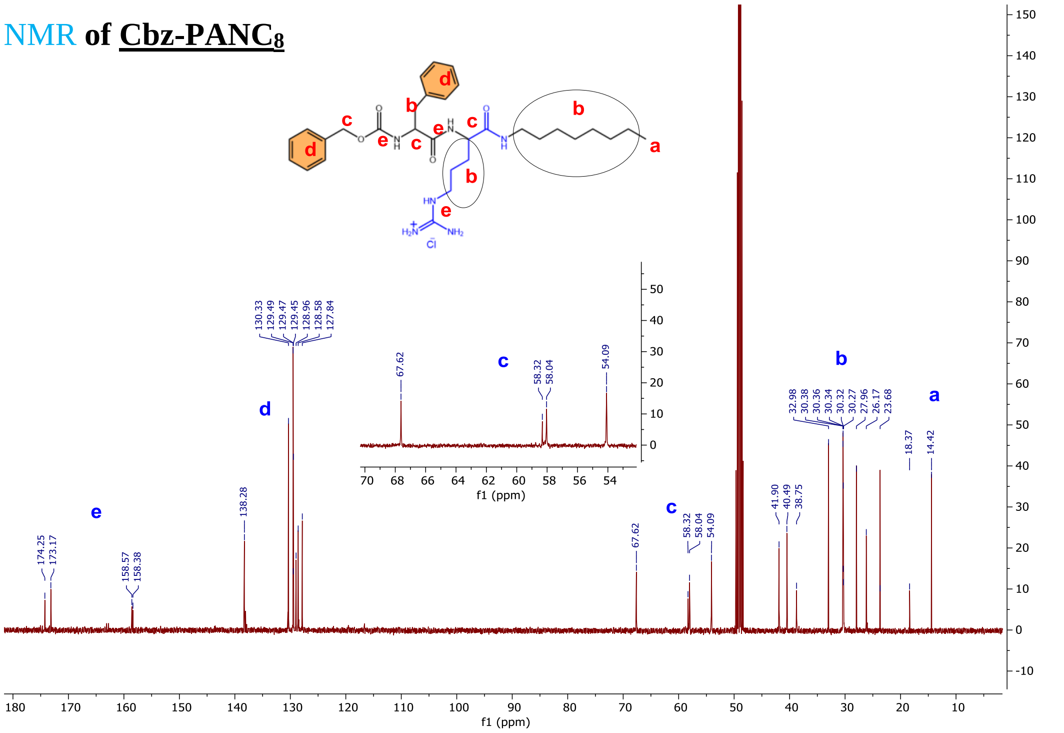

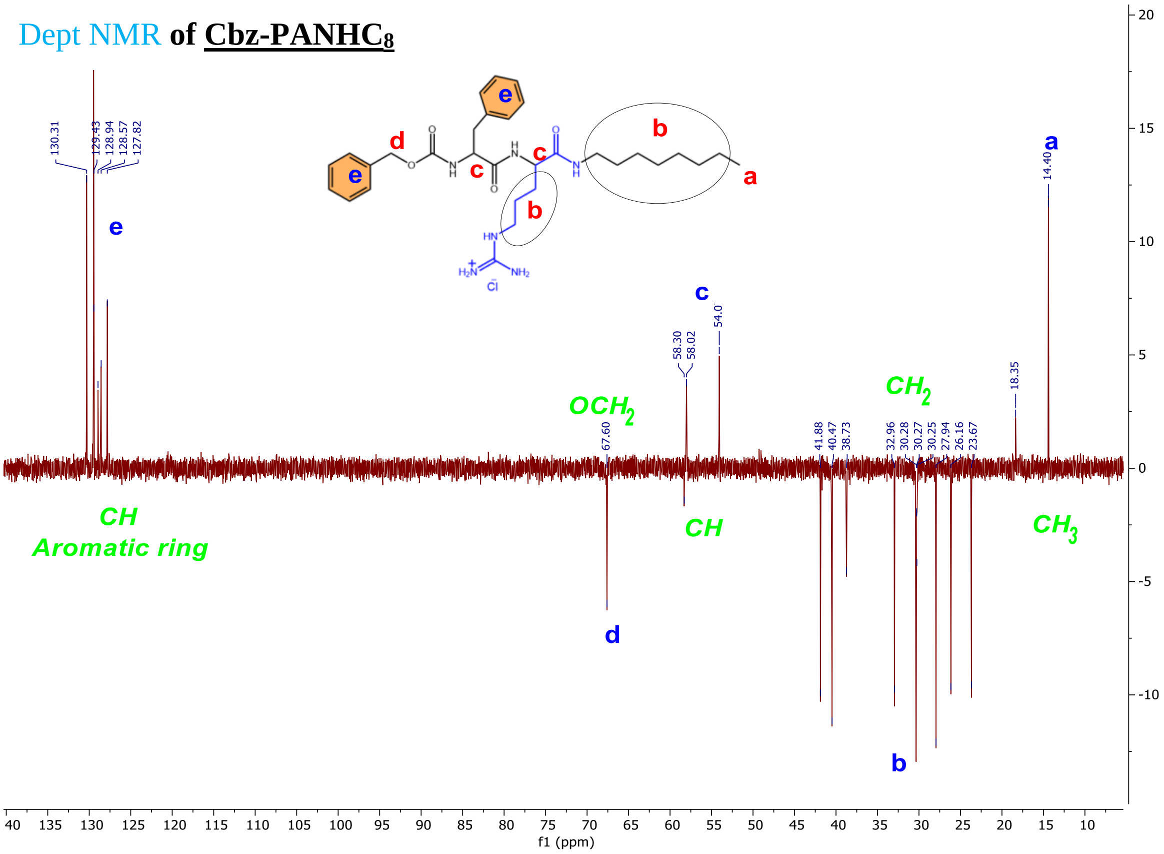

Figure S9. HPLC, ESI-MS, ^{1}H NMR,^{13}C NMR and Dept of Cbz-PANHC8

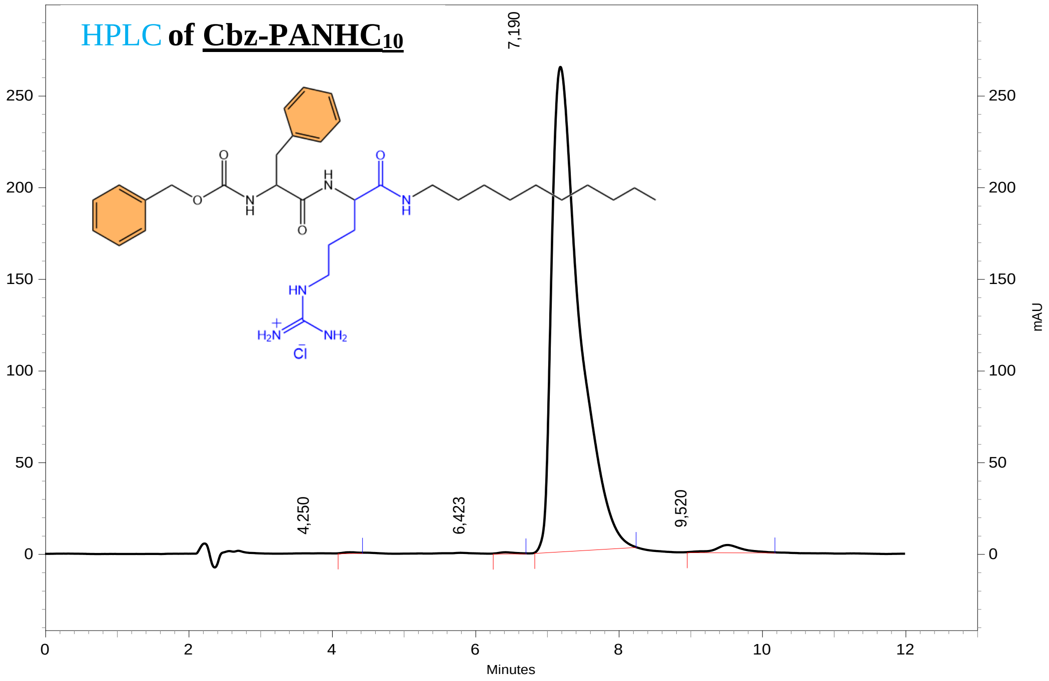

ESI-MS of Cbz-PANHC$_{10}$

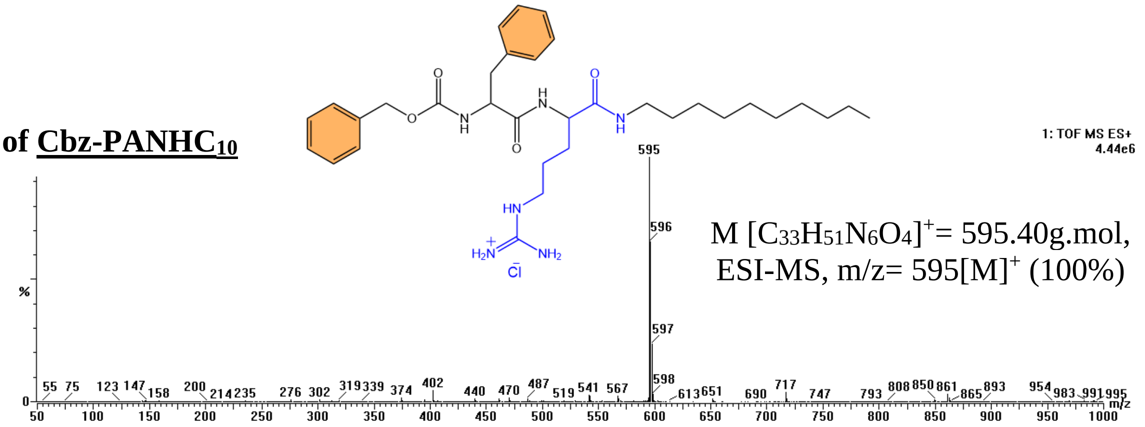

^{1}H NMR of Cbz-PANHC$_{10}$

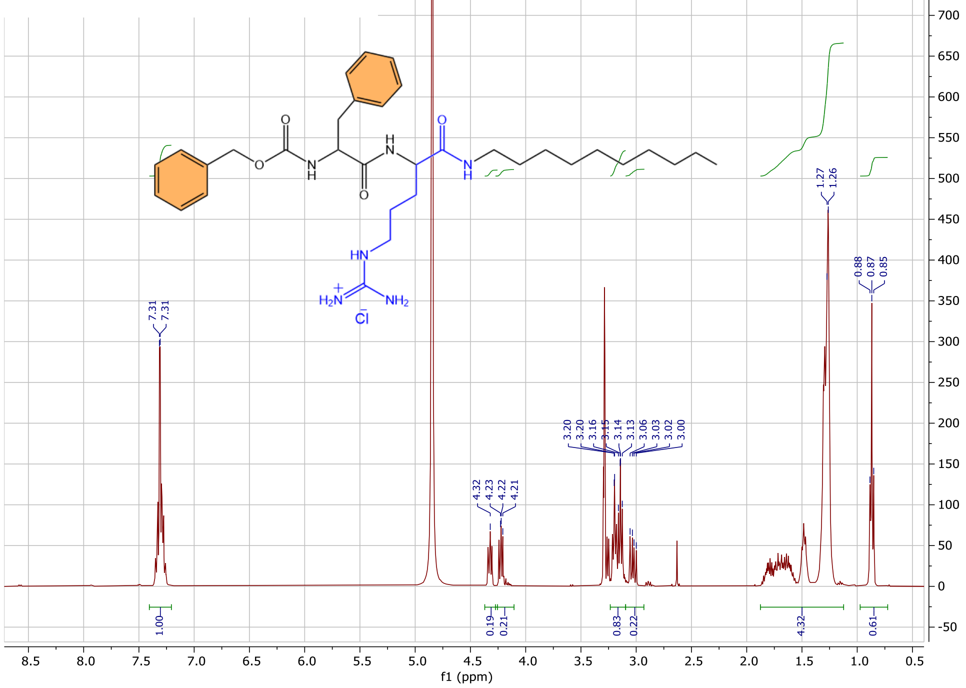

^{13}C NMR of Cbz-PANC$_{10}$

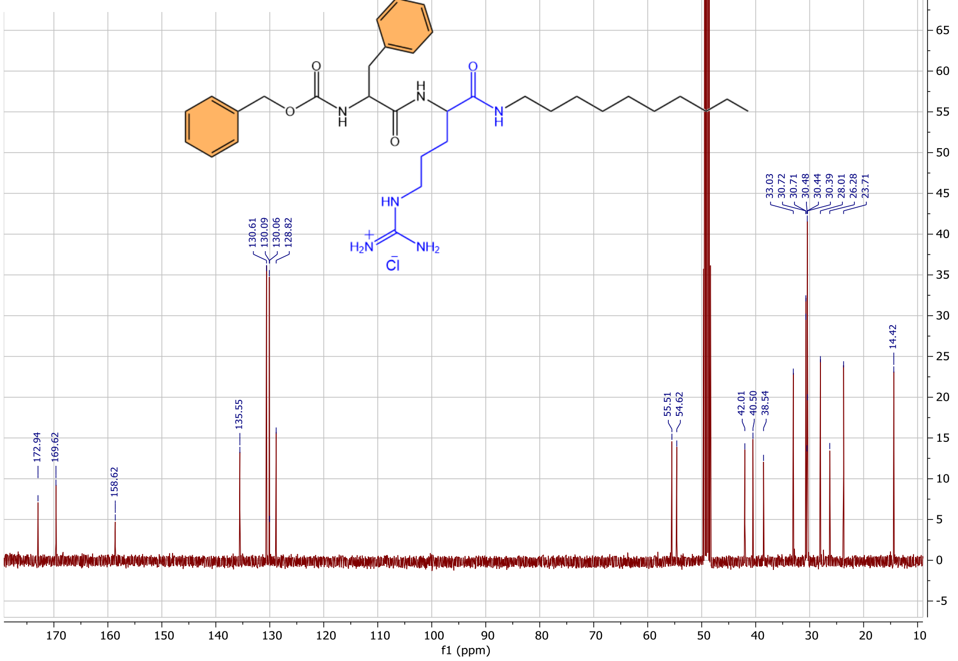

Dept NMR of Cbz-PANHC10

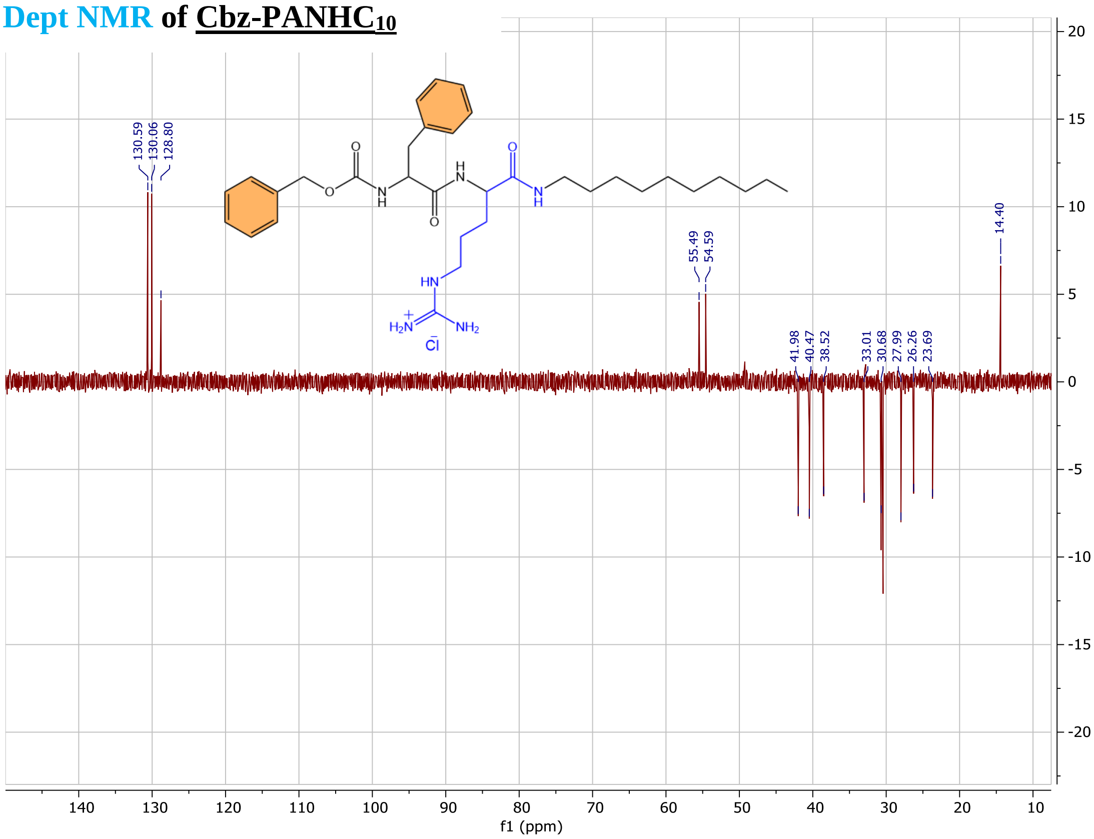

Figure S10. HPLC, ESI-MS, ^{1}H NMR, ^{13}C NMR and Dept of Cbz-PANHC$_{10}$

HPLC of **Cbz-PANHC$_{12}$**

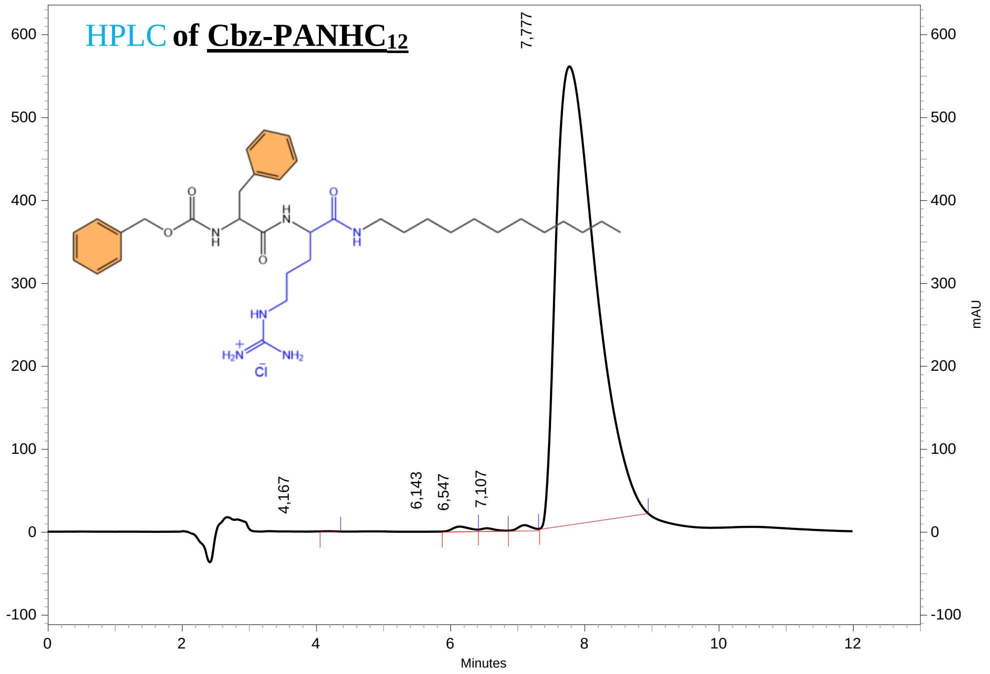

ESI-MS ***of*** **Cbz-PANHC$_{12}$**

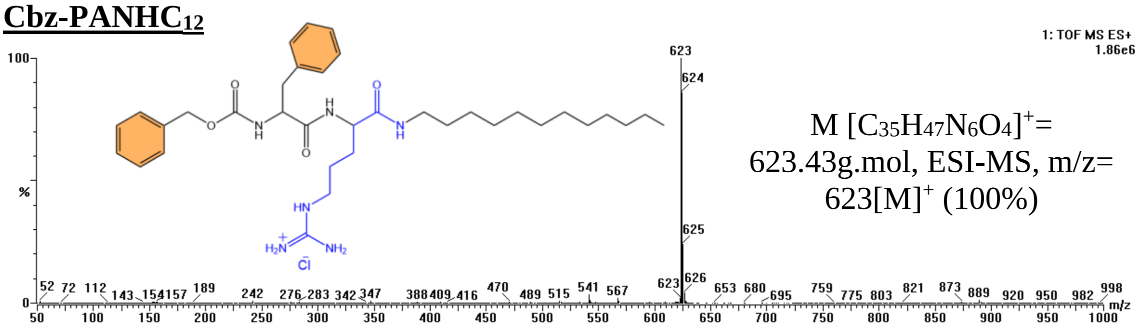

M $[C_{35}H_{47}N_6O_4]^+$= 623.43g.mol, ESI-MS, m/z= $623[M]^+$ (100%)

^{1}H NMR **of <u>Cbz-PANHC$_{12}$</u>**

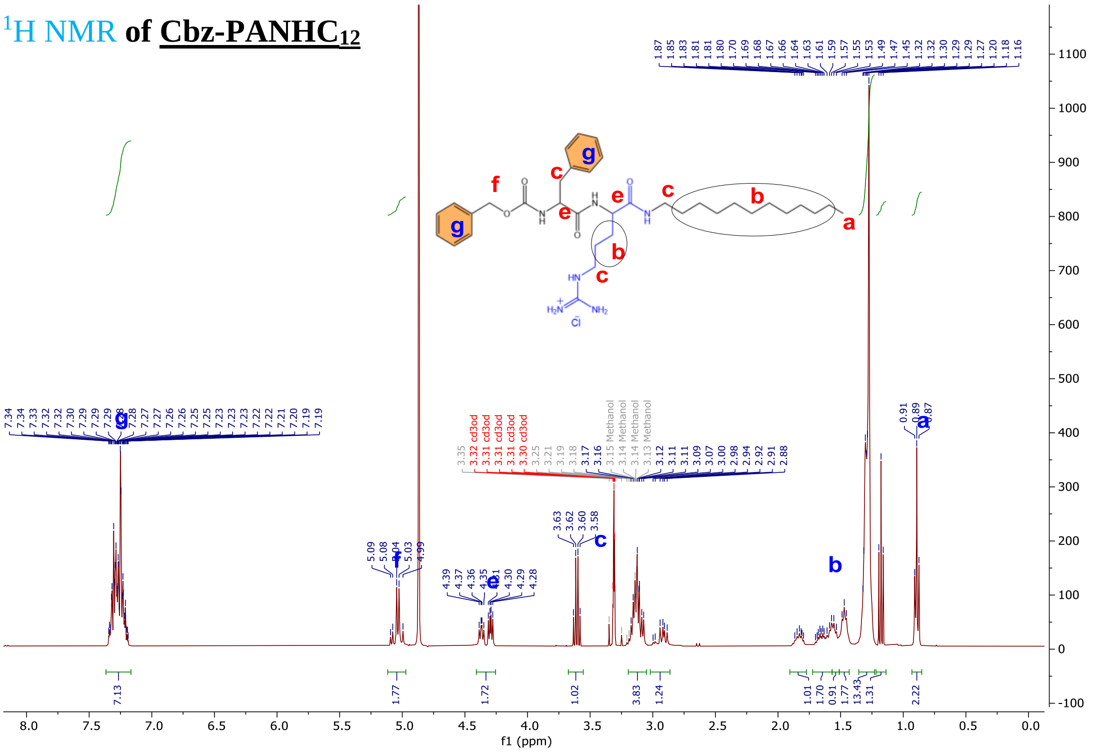

^{13}C NMR **of <u>Cbz-PANHC$_{12}$</u>**

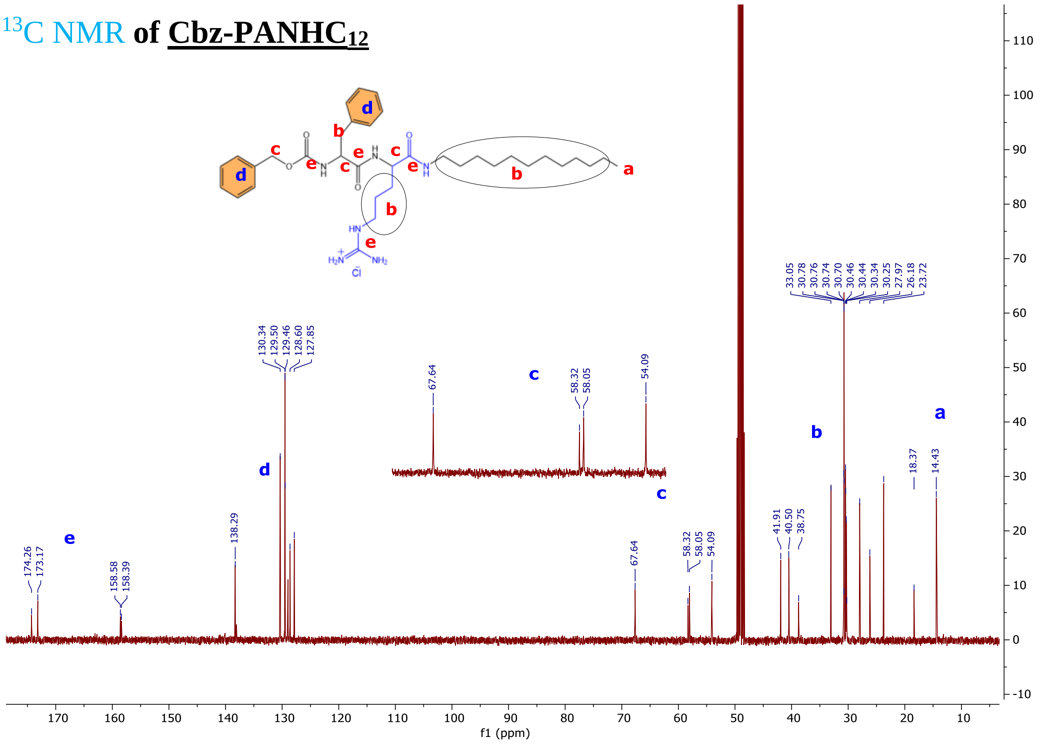

Dept NMR **of <u>Cbz-PANHC₁₂</u>**

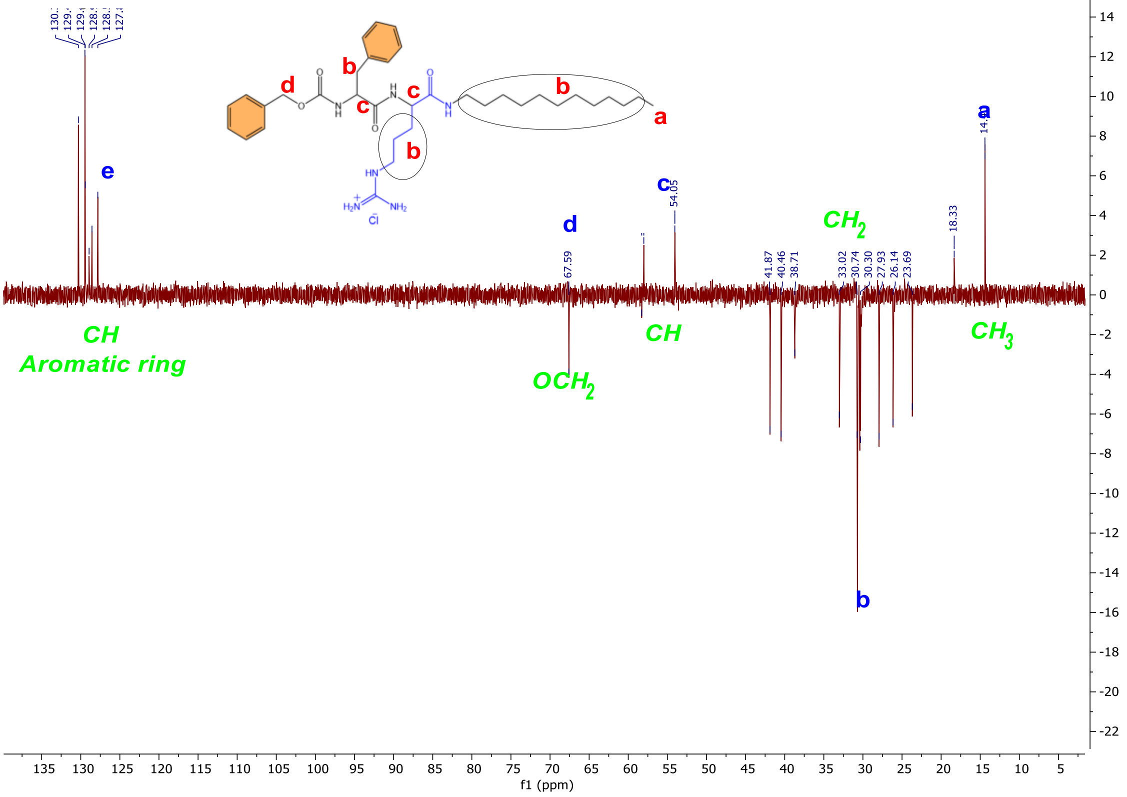

Figure S11. HPLC, ESI-MS, ^{1}H NMR,^{13}C NMR and Dept of Cbz-PANHC$_{12}$

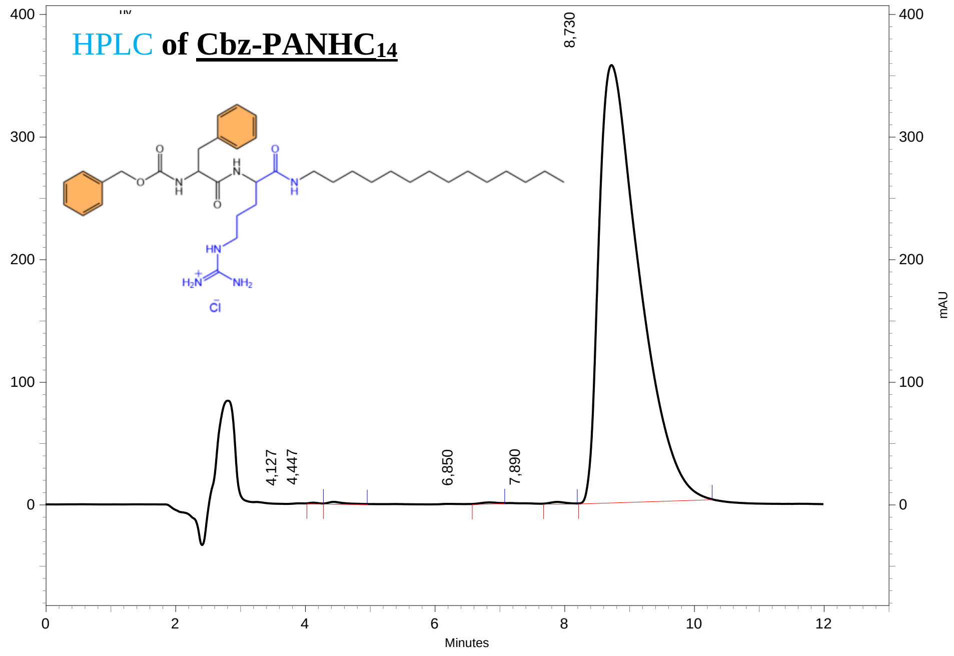

ESI-MS of **Cbz-PANHC14**

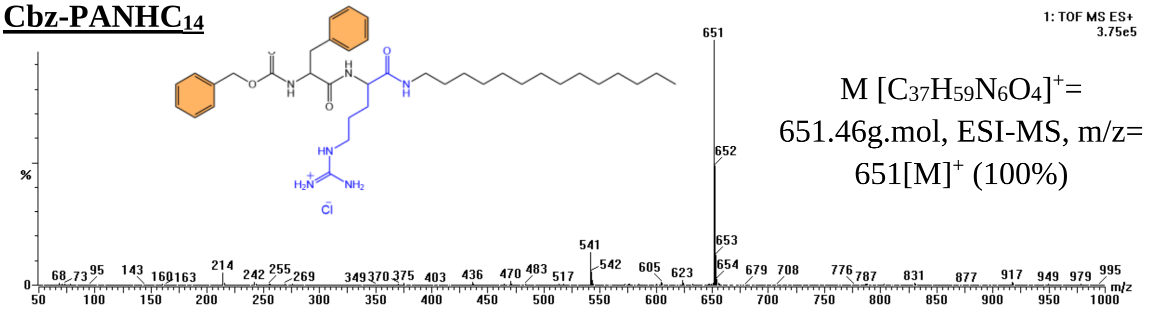

^{1}H NMR **of Cbz-PANHC$_{14}$**

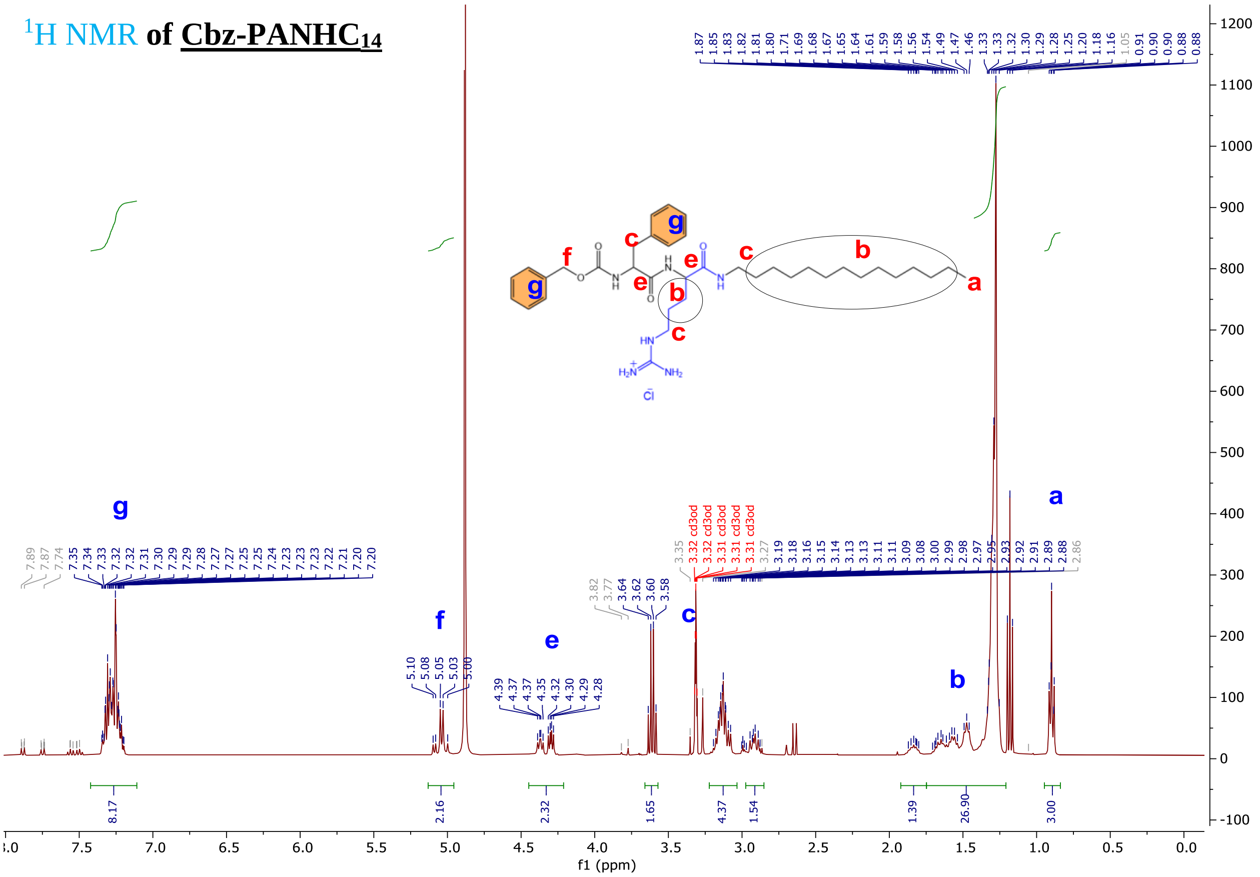

^{13}C NMR *of* **Cbz-PANHC$_{14}$**

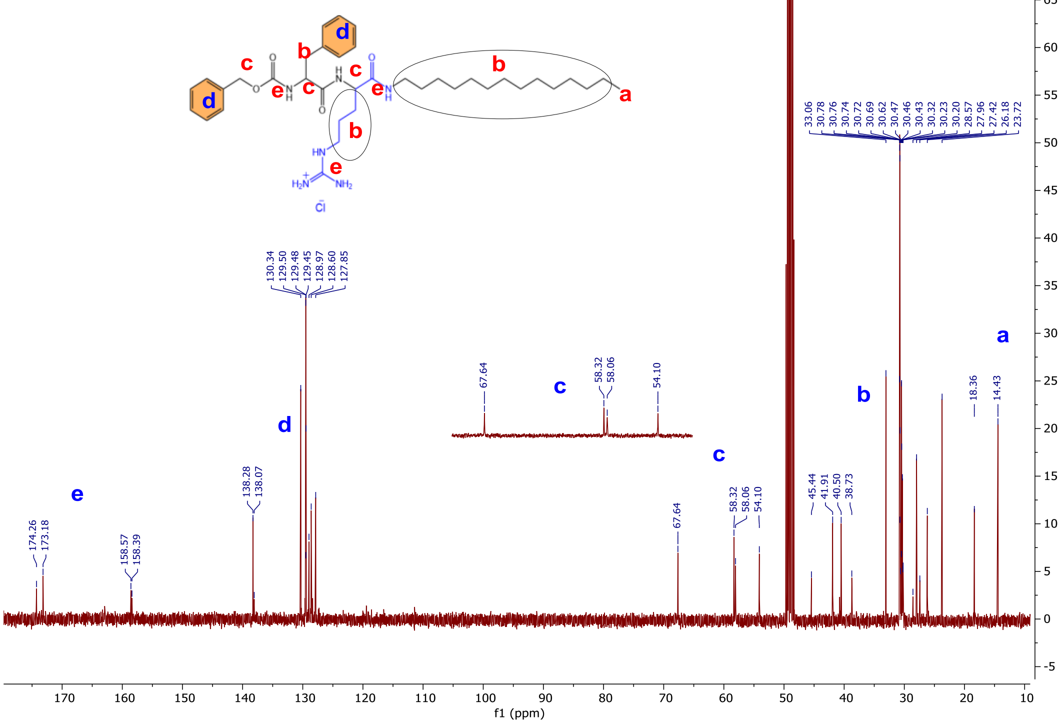

Dept NMR *of* **Cbz-PANHC$_{14}$**

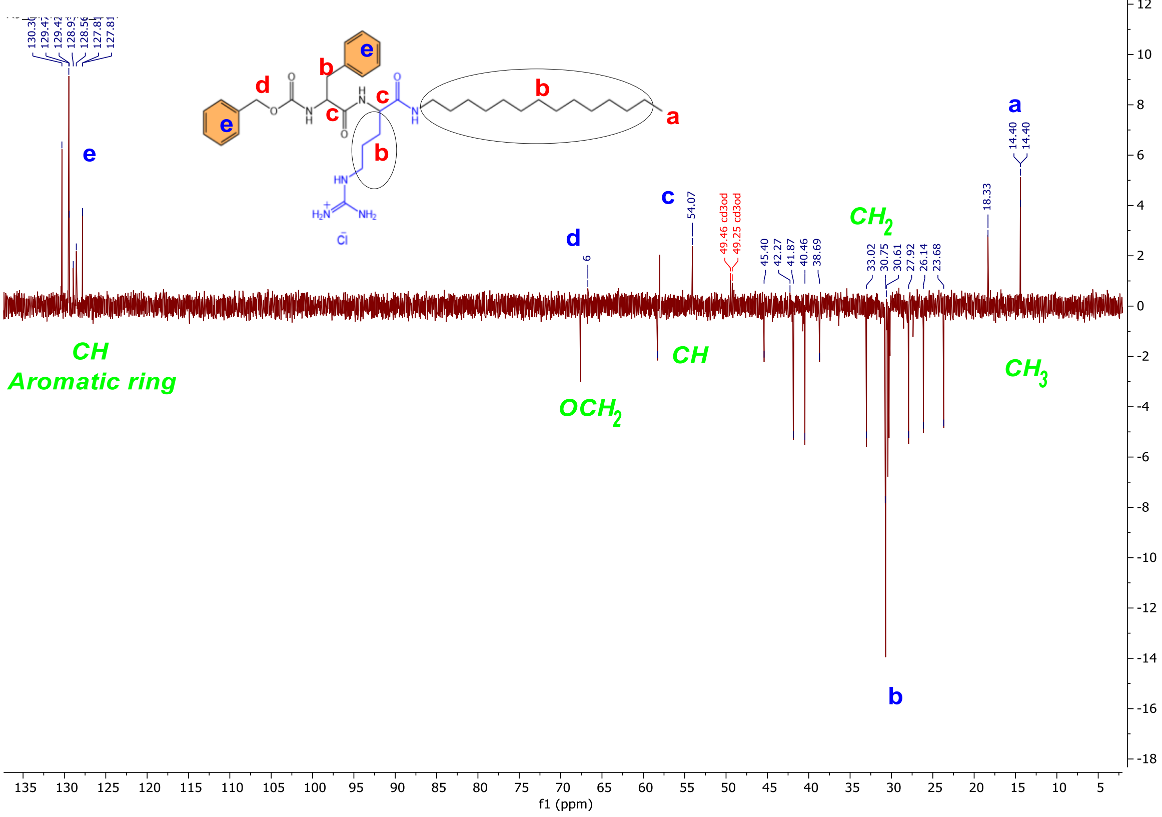

Figure S12. HPLC, ESI-MS, ^{1}H NMR, ^{13}C NMR and Dept of Cbz-PANHC$_{14}$

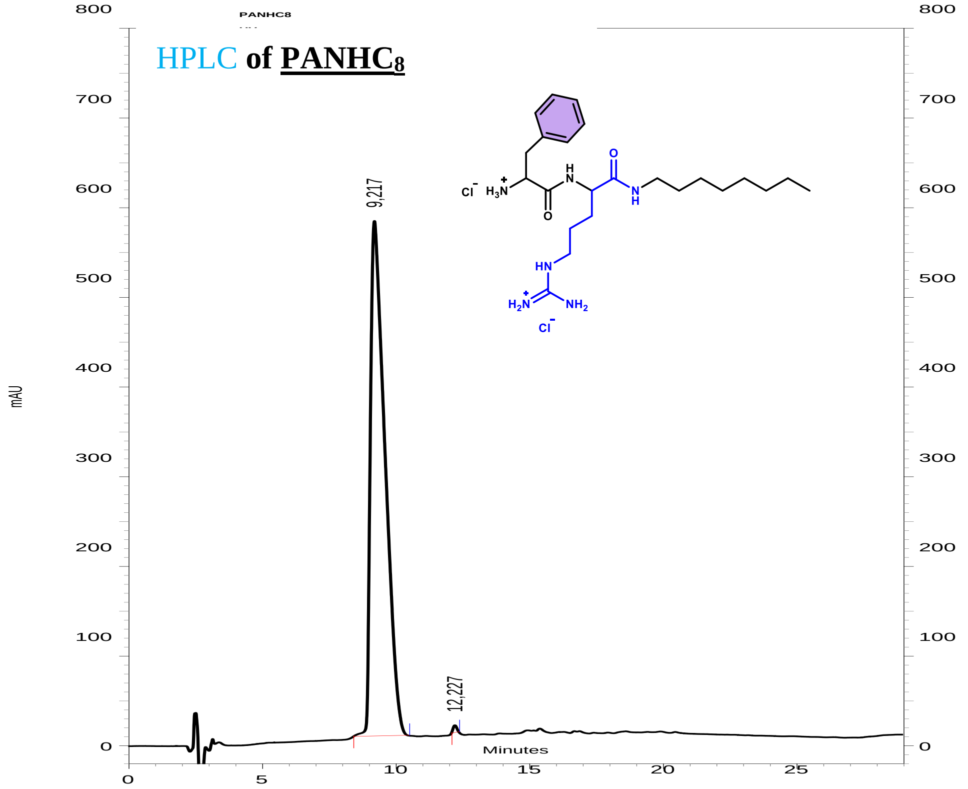

ESI-MS *of* **PANHC8**

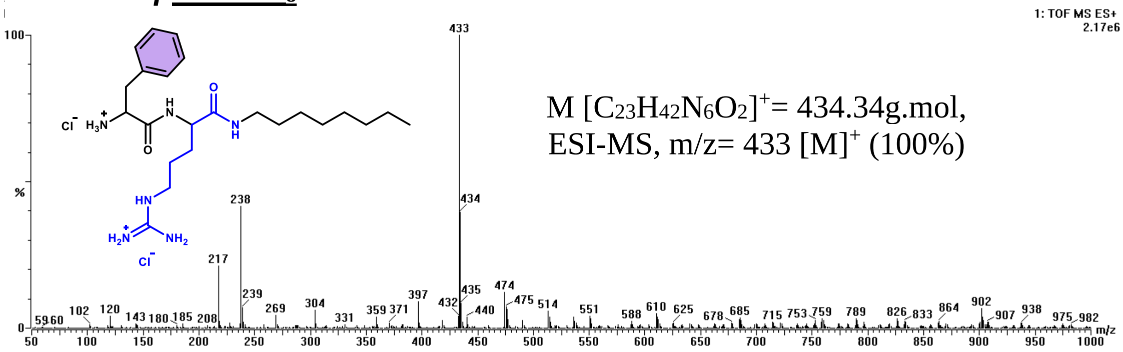

^{1}H NMR *of* **PANHC$_8$**

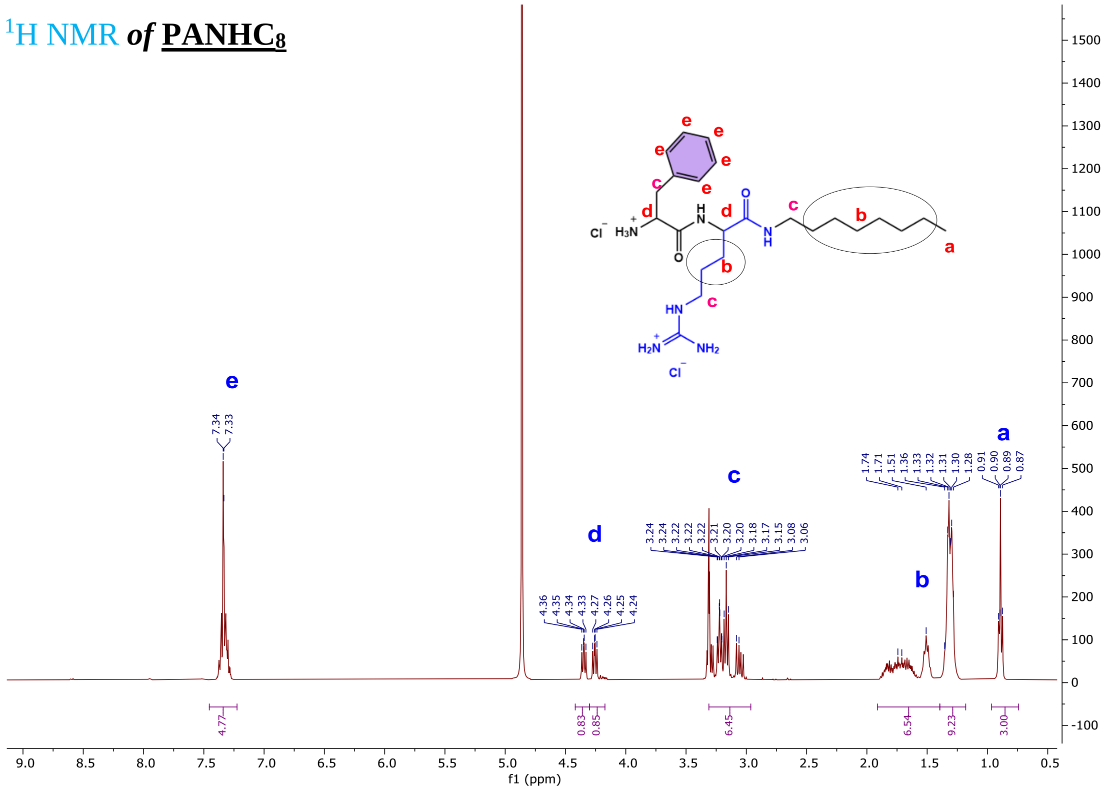

^{13}C NMR *of* **PANHC$_8$**

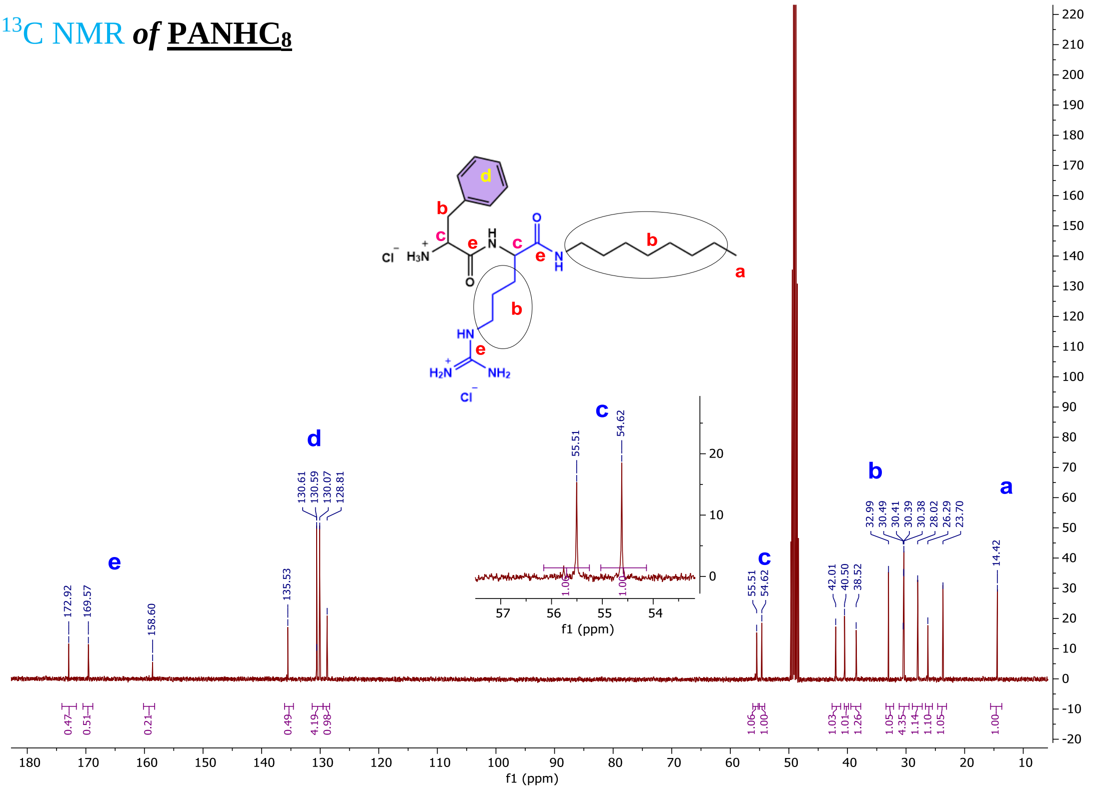

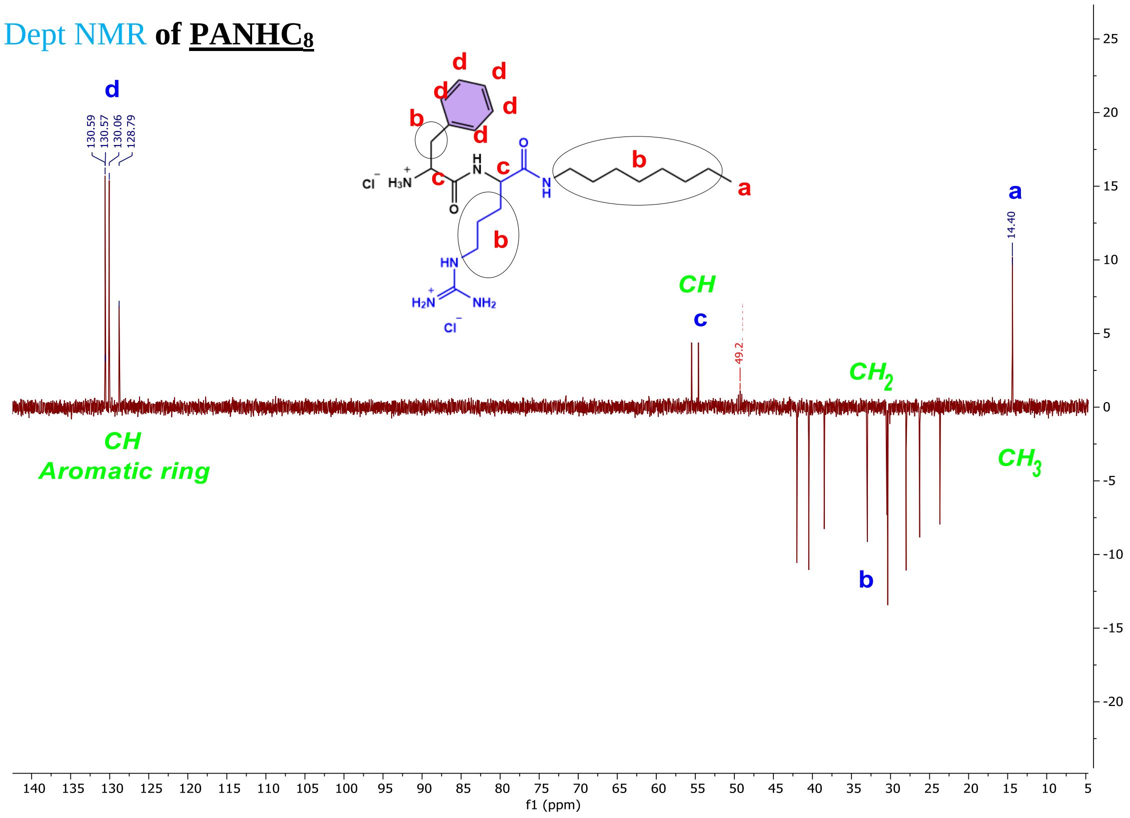

. **Figure S13.** HPLC, ESI-MS, ^{1}H NMR, ^{13}C NMR and Dept of PANHC8

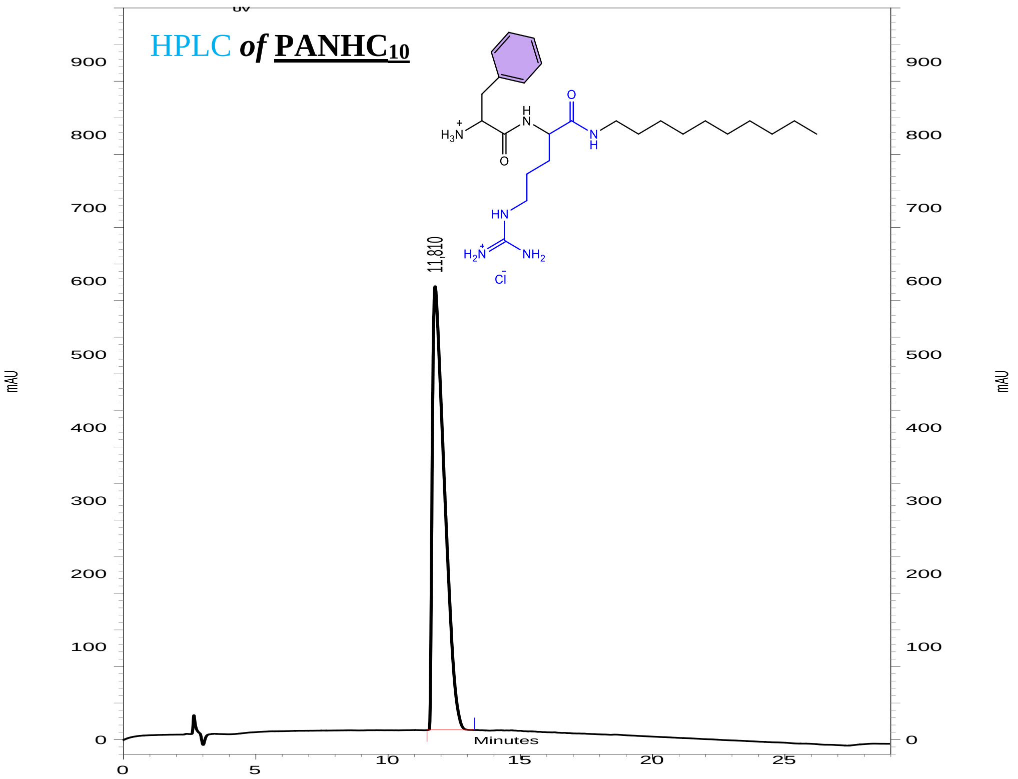

ESI-MS **of PANHC$_{10}$**

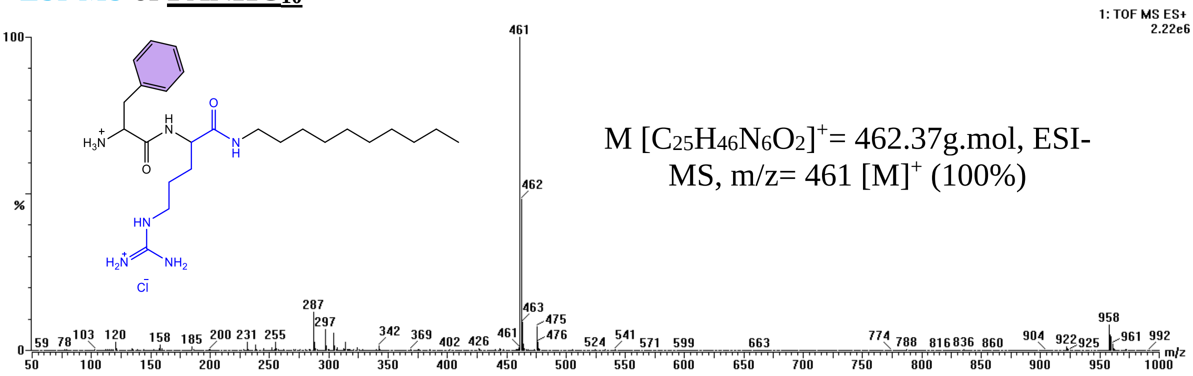

^{1}H NMR of **PANHC$_{10}$**

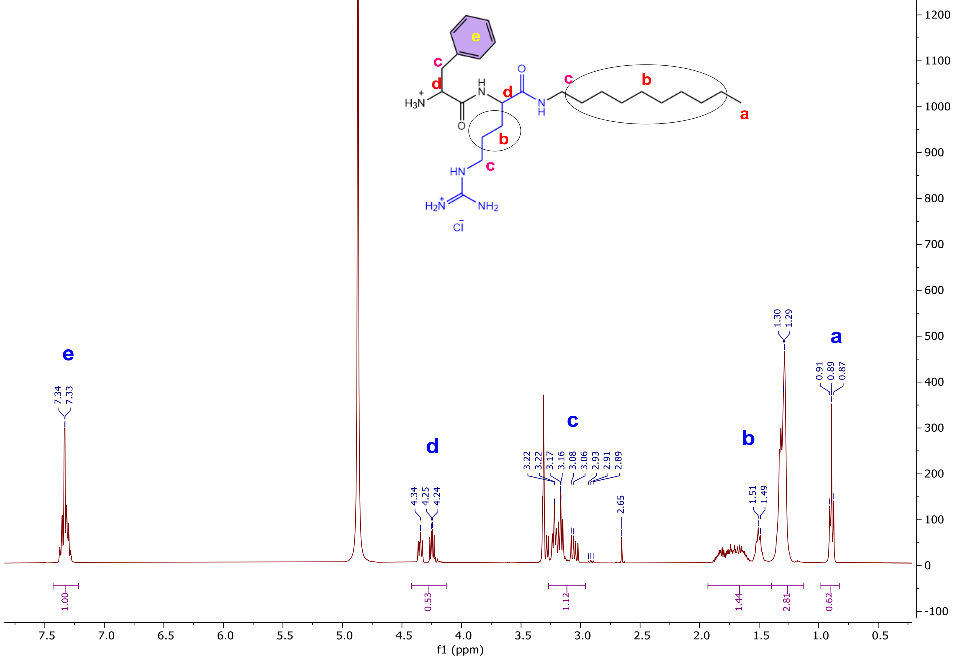

^{13}C NMR of **PANHC$_{10}$**

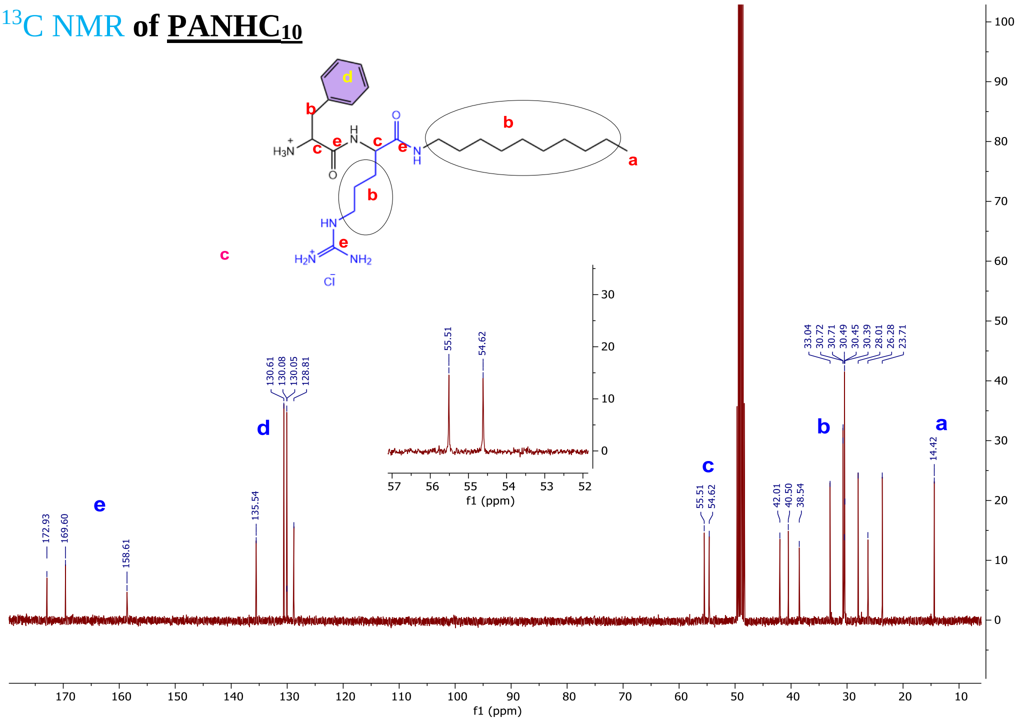

Dept NMR **of** **PANHC10**

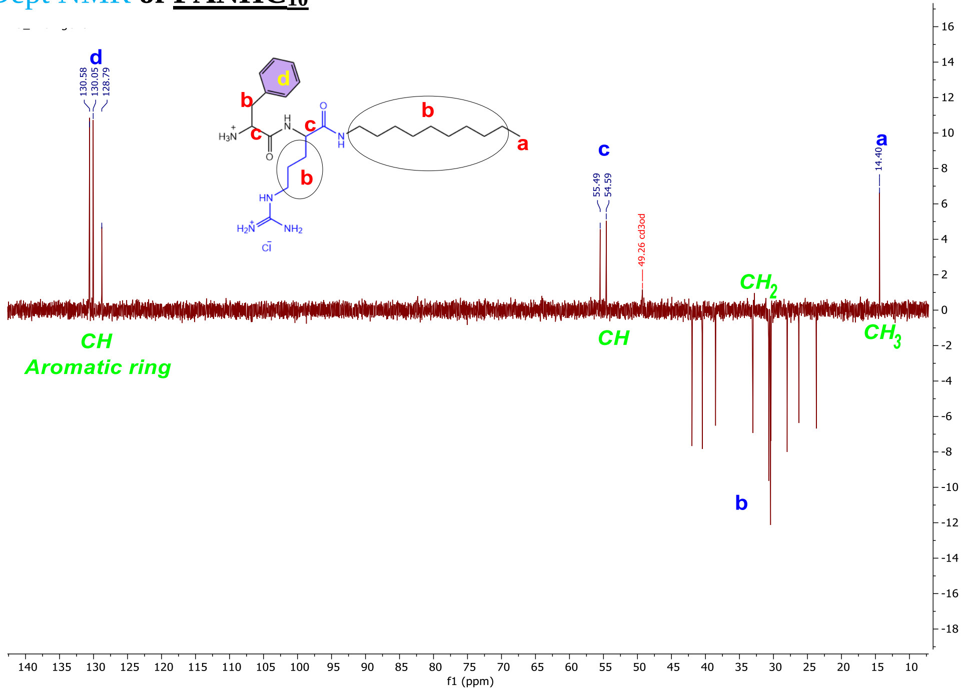

. **Figure S14.** HPLC, ESI-MS, ^1H NMR, ^{13}C NMR and Dept of $PANHC_{10}$

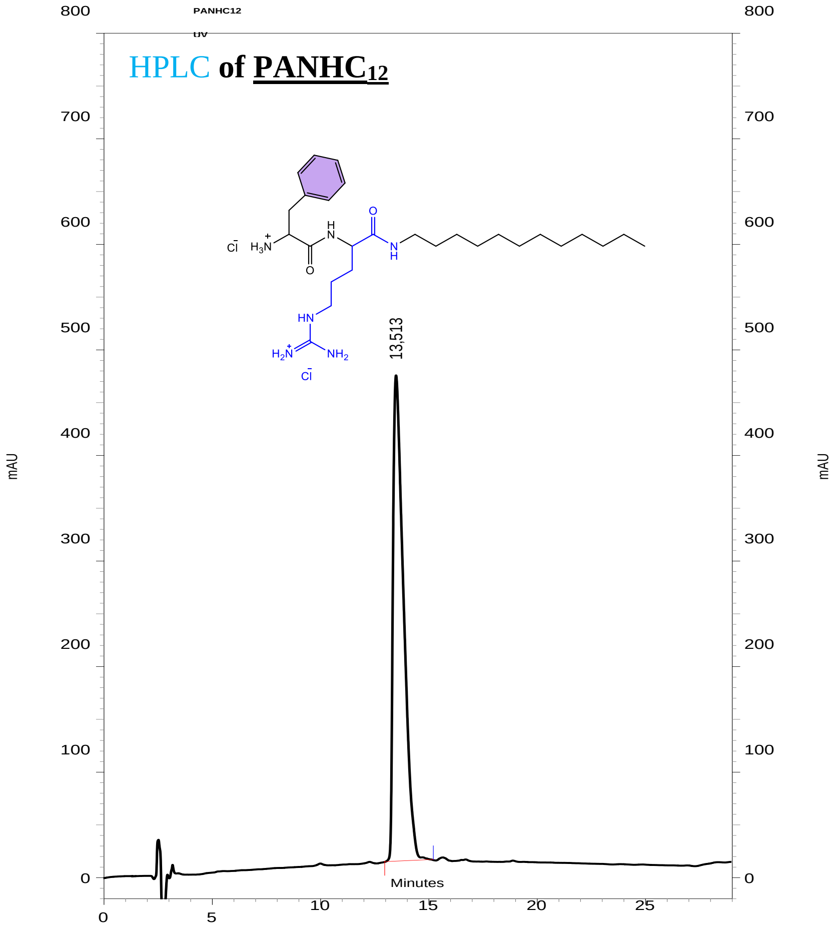

ESI-MS of PANHC12

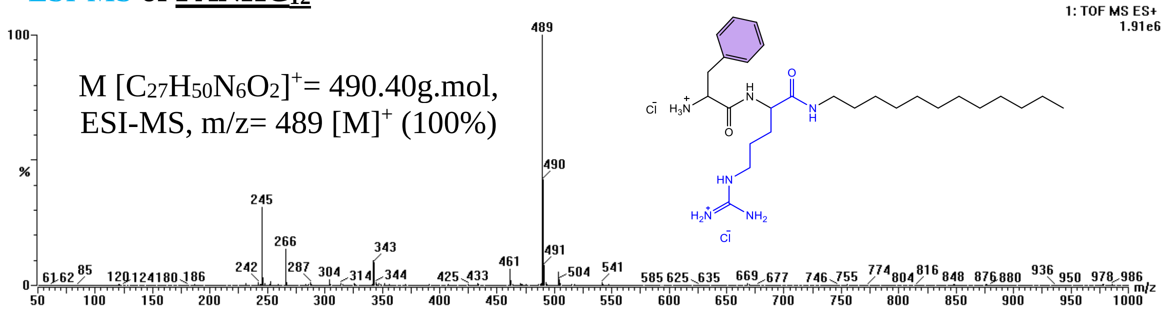

^{1}H NMR of **PANHC$_{12}$**

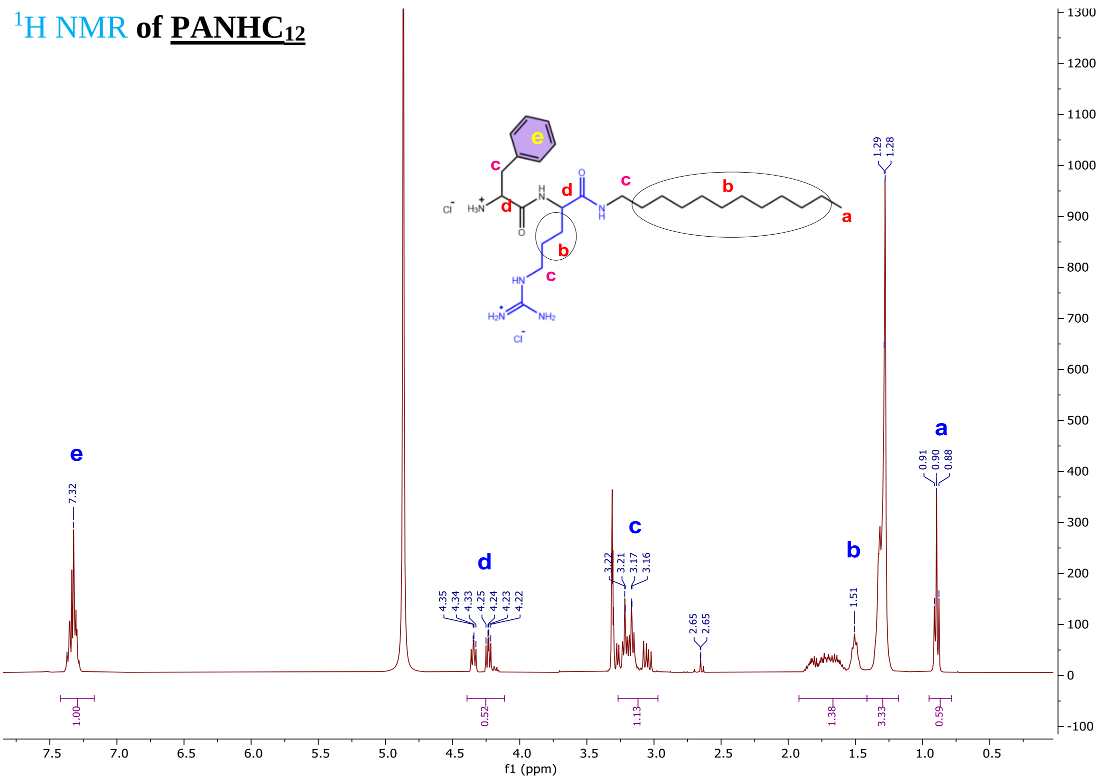

^{13}C NMR of **PANHC$_{12}$**

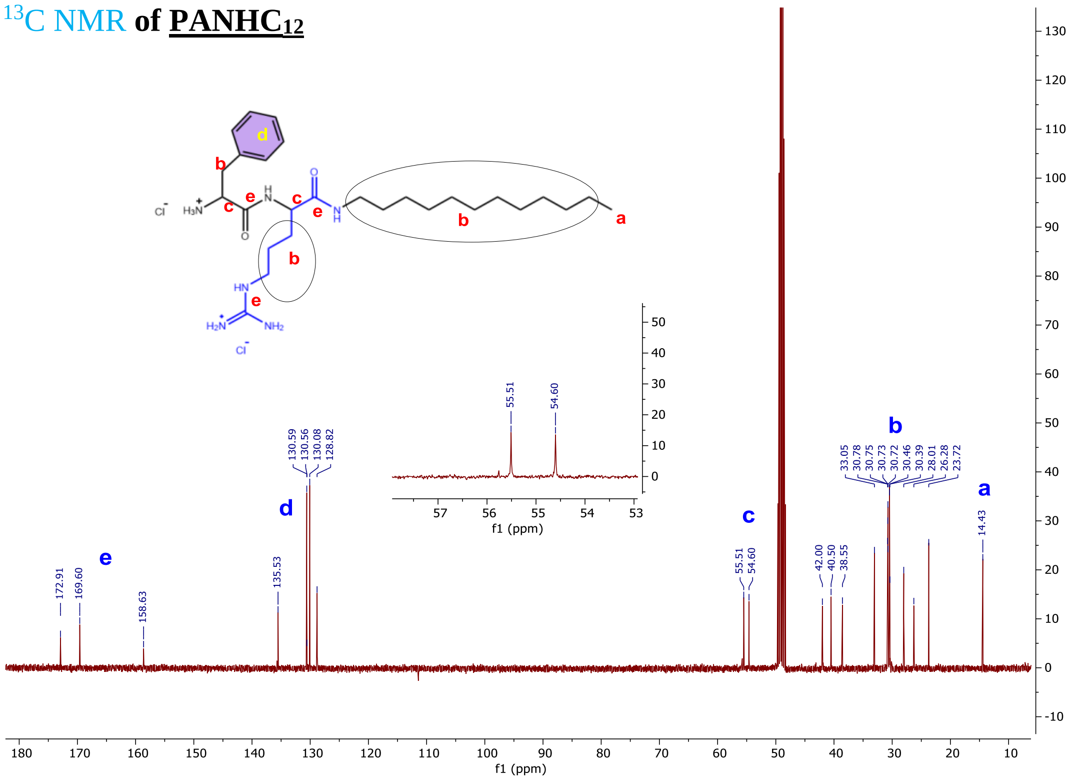

Dept NMR **of PANHC$_{12}$**

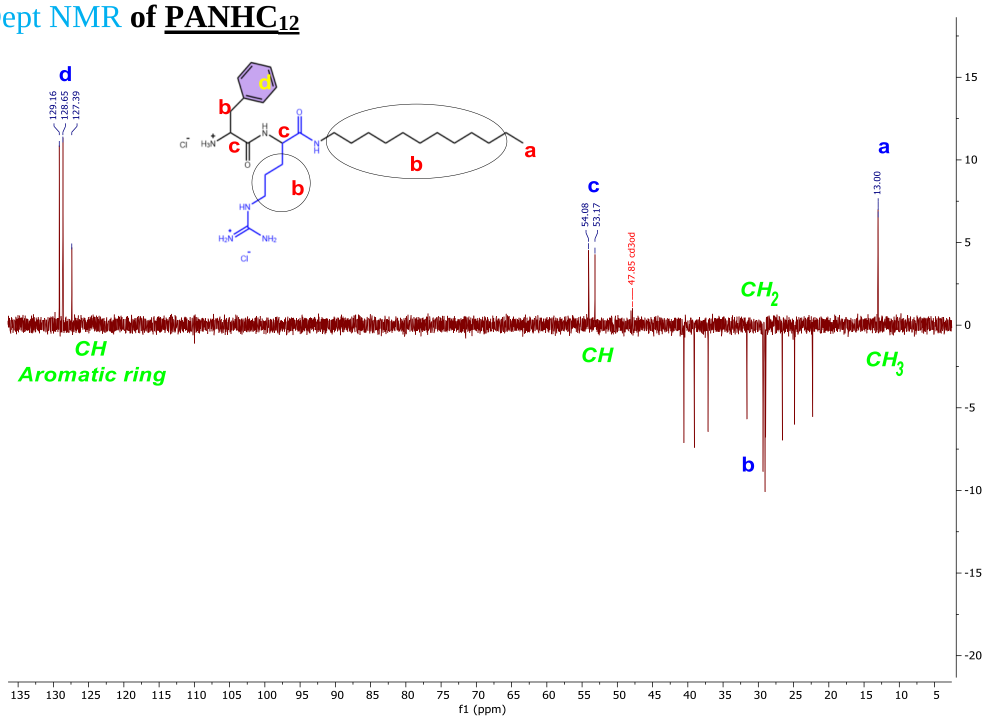

. **Figure S15.** HPLC, ESI-MS, ^{1}H NMR, ^{13}C NMR and Dept of PANHC$_{12}$

HPLC of **PANHC$_{14}$**

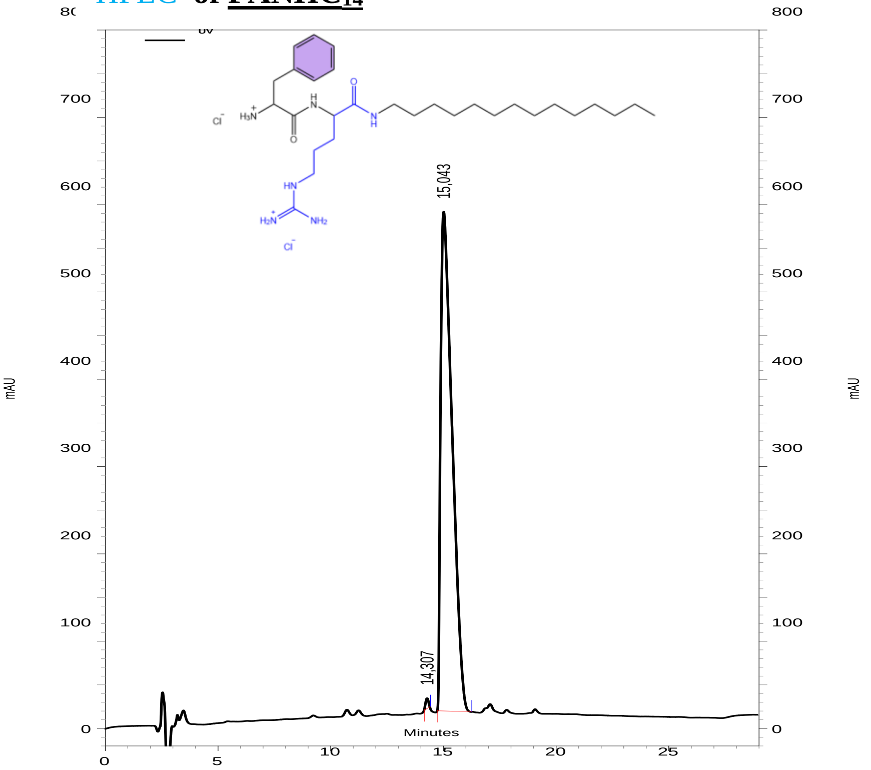

ESI-MS of **PANHC$_{14}$**

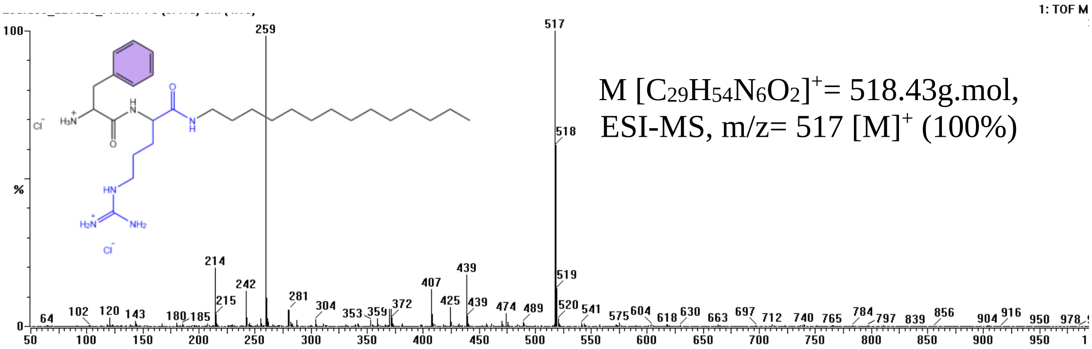

M $[C_{29}H_{54}N_6O_2]^{+}$= 518.43g.mol,
ESI-MS, m/z= 517 $[M]^{+}$ (100%)

^{1}H NMR of PANHC$_{14}$

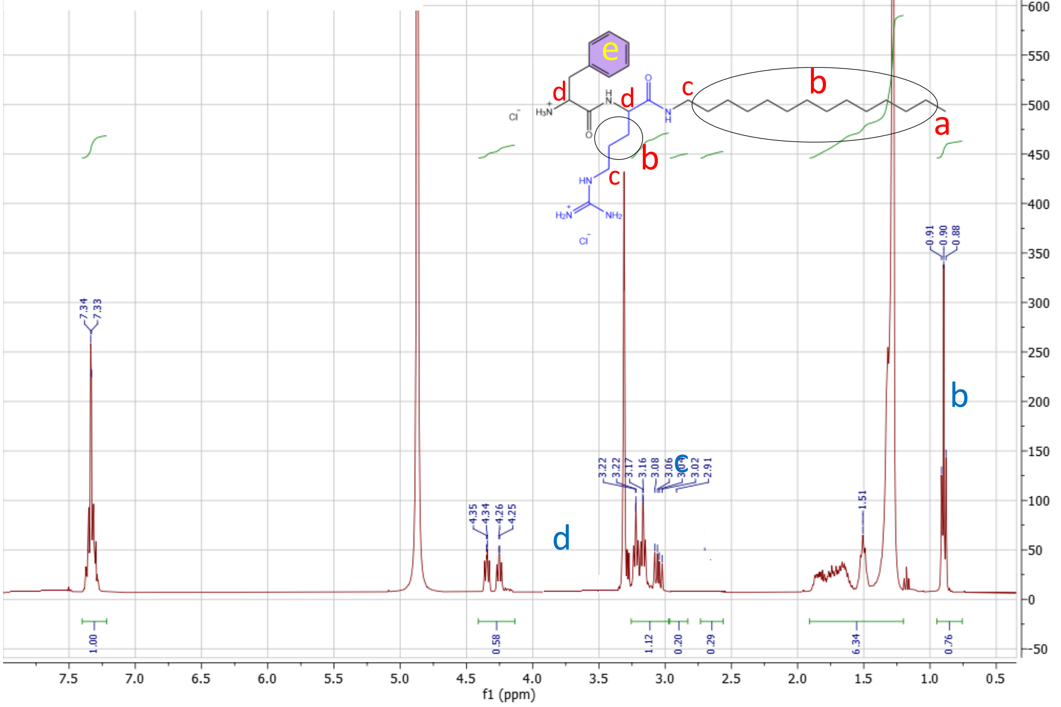

^{13}C NMR of PANHC$_{14}$

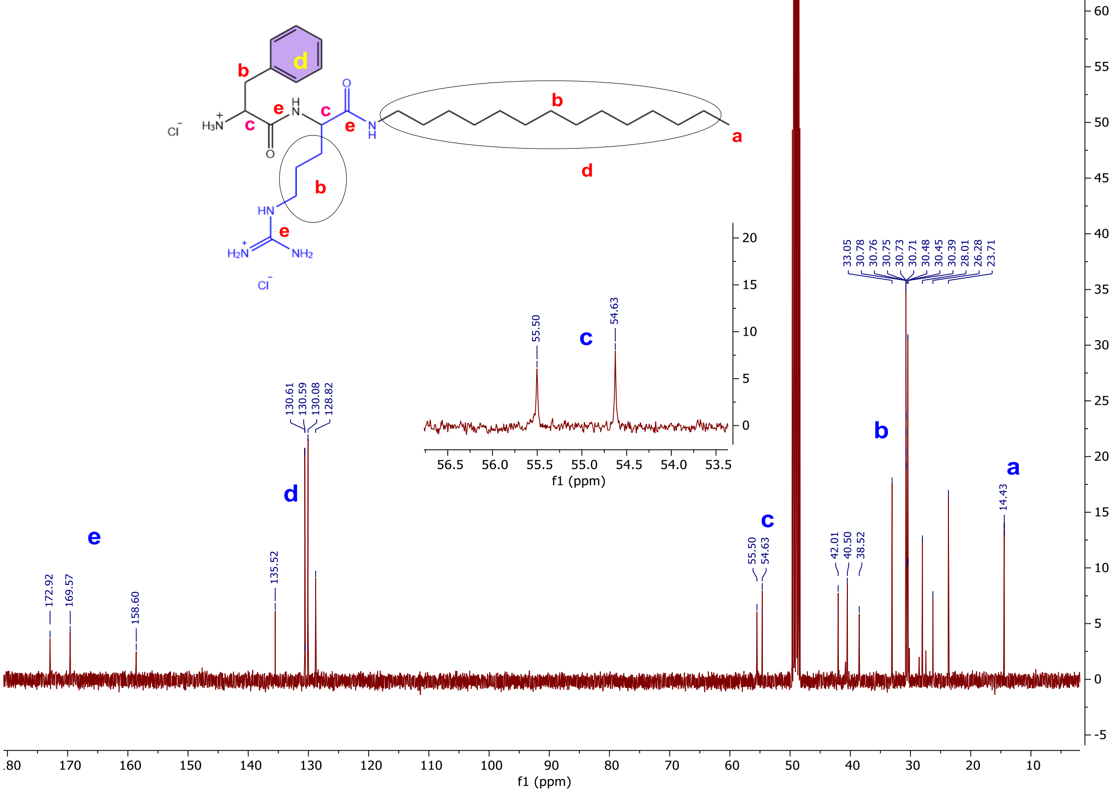

Dept NMR **of** **PANHC14**

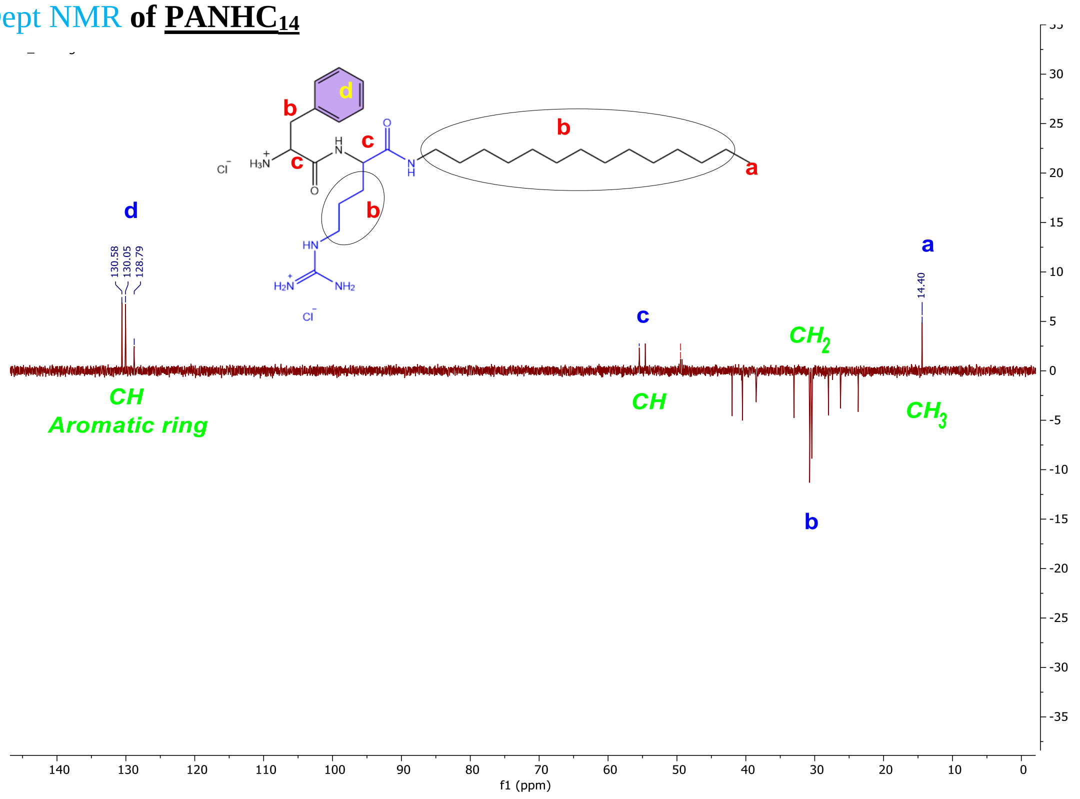

Figure S16. HPLC, ESI-MS, ^{1}H NMR, ^{13}C NMR and Dept of PANHC14

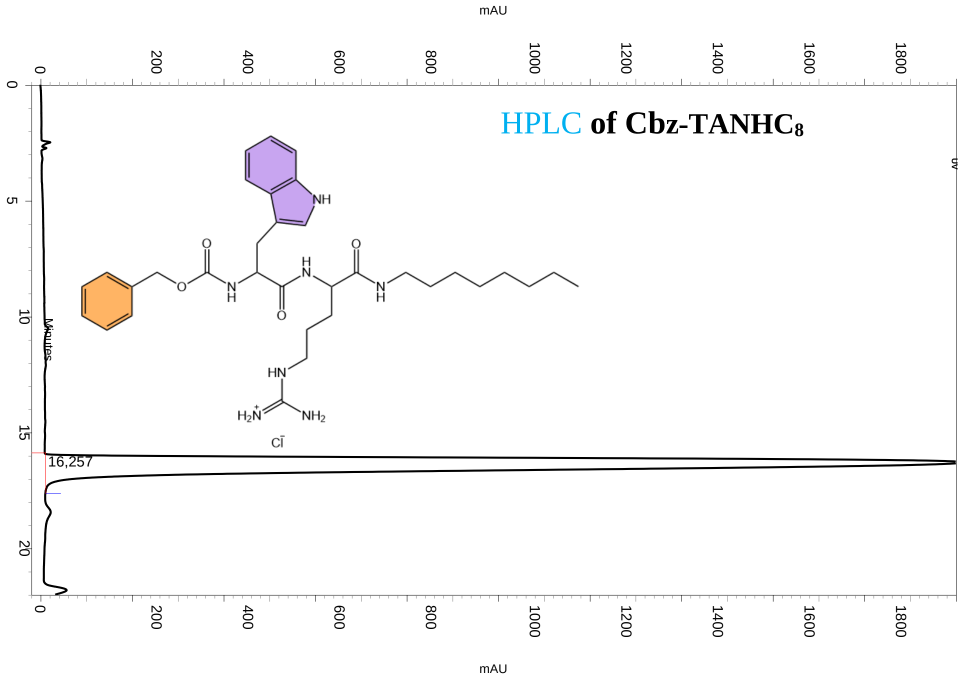

ESI-MS of Cbz-TANHC$_8$

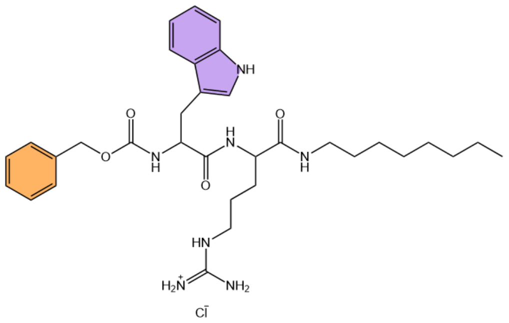

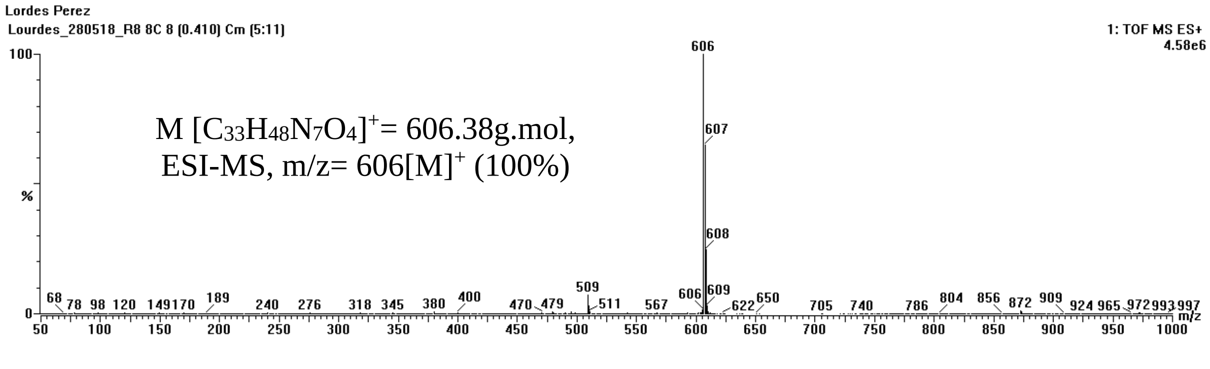

^{1}H NMR of Cbz-TANHC$_8$

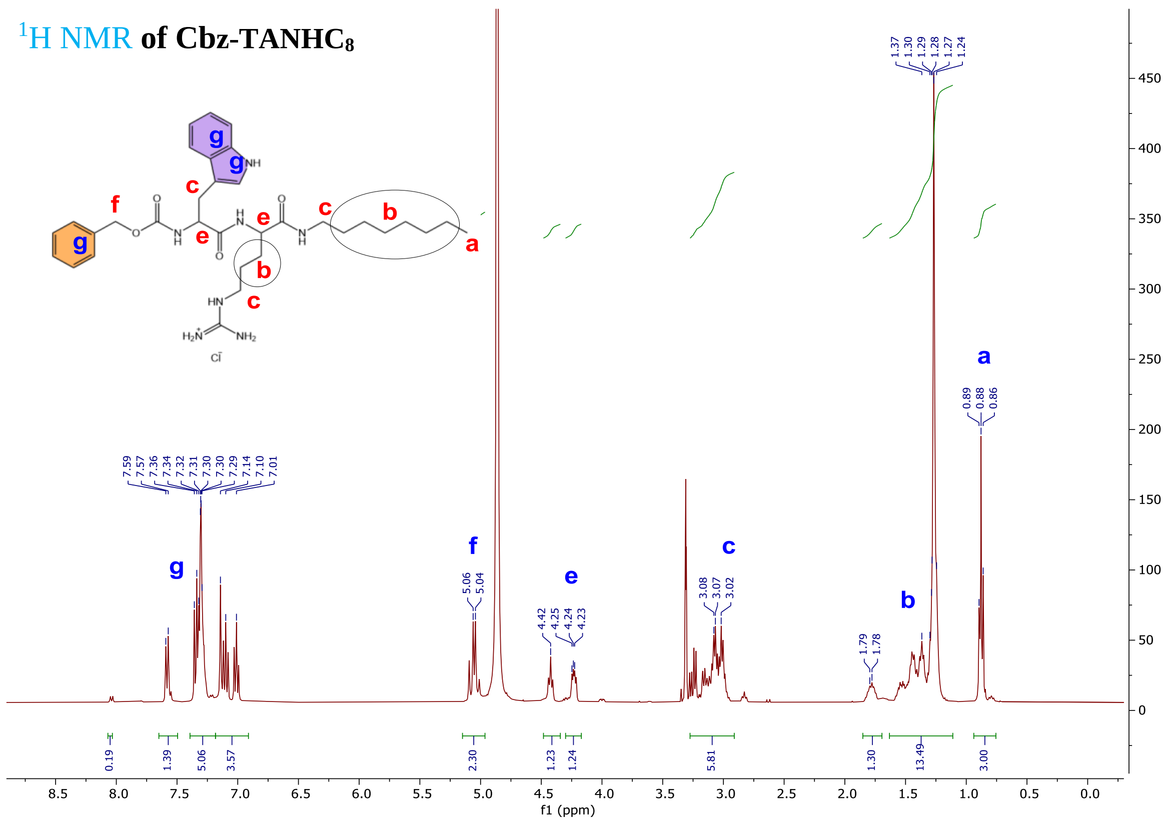

^{13}C NMR of Cbz-TANHC$_8$

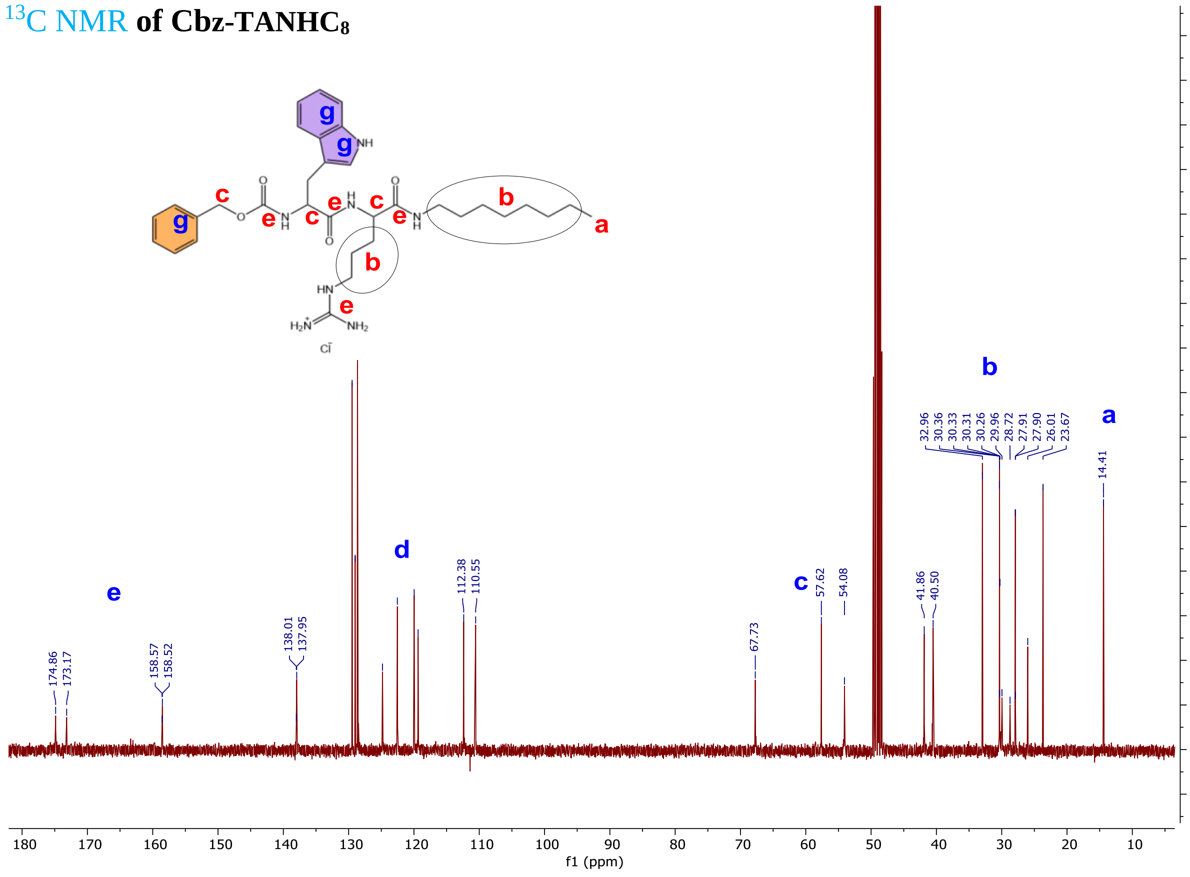

Dept NMR **of Cbz-TANHC$_8$**

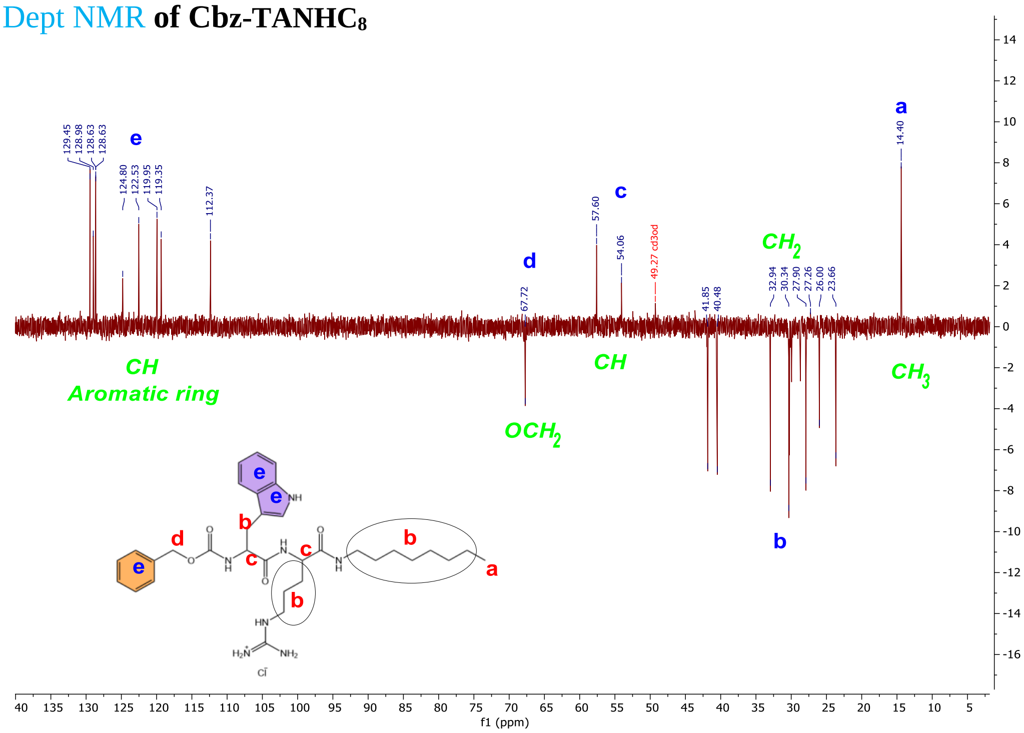

. **Figure S17.** HPLC, ESI-MS, ^{1}H NMR,^{13}C NMR and Dept of Cbz-TANHC$_8$

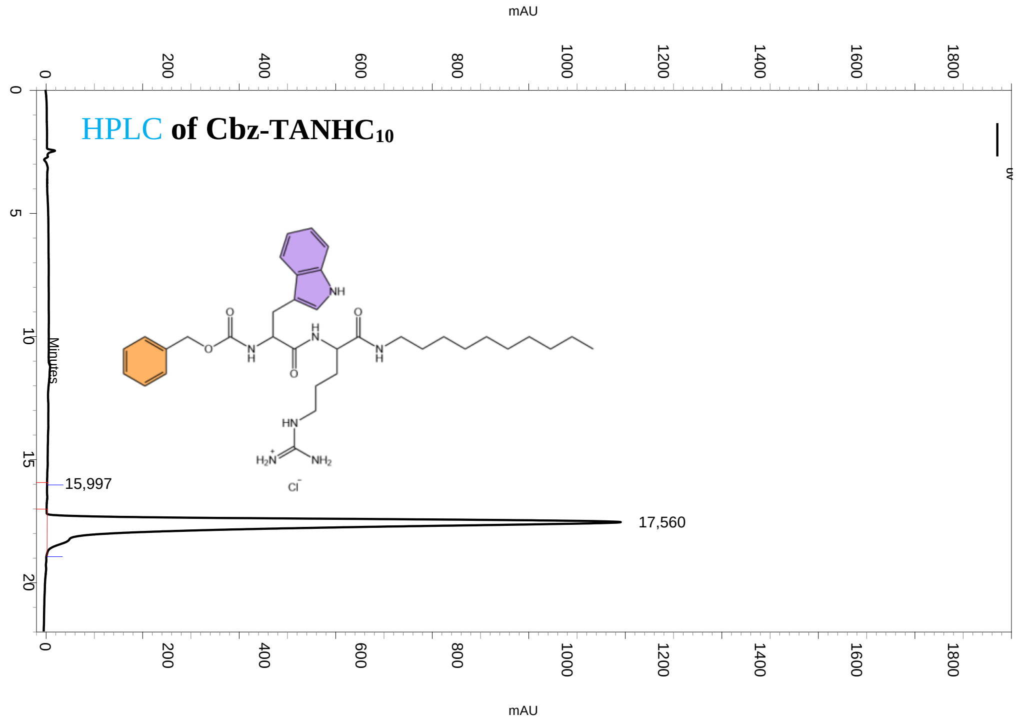

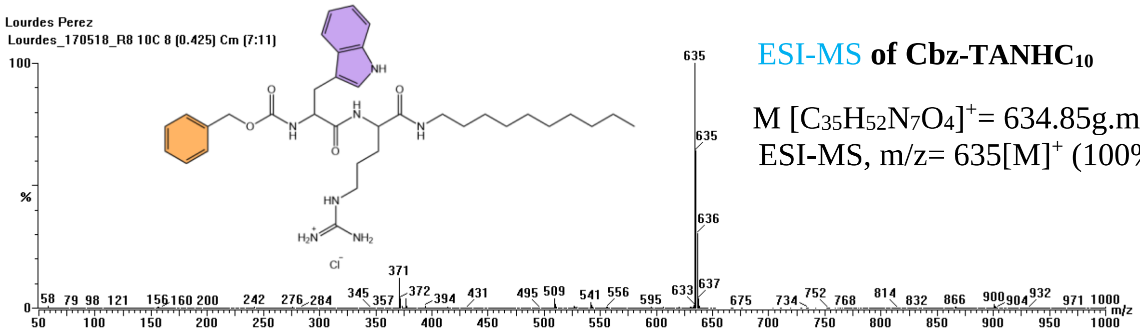

ESI-MS **of Cbz-TANHC$_{10}$**

M $[C_{35}H_{52}N_7O_4]^+$= 634.85g.mol,
ESI-MS, m/z= 635$[M]^+$ (100%)

^{1}H NMR **of Cbz-TANHC$_{10}$**

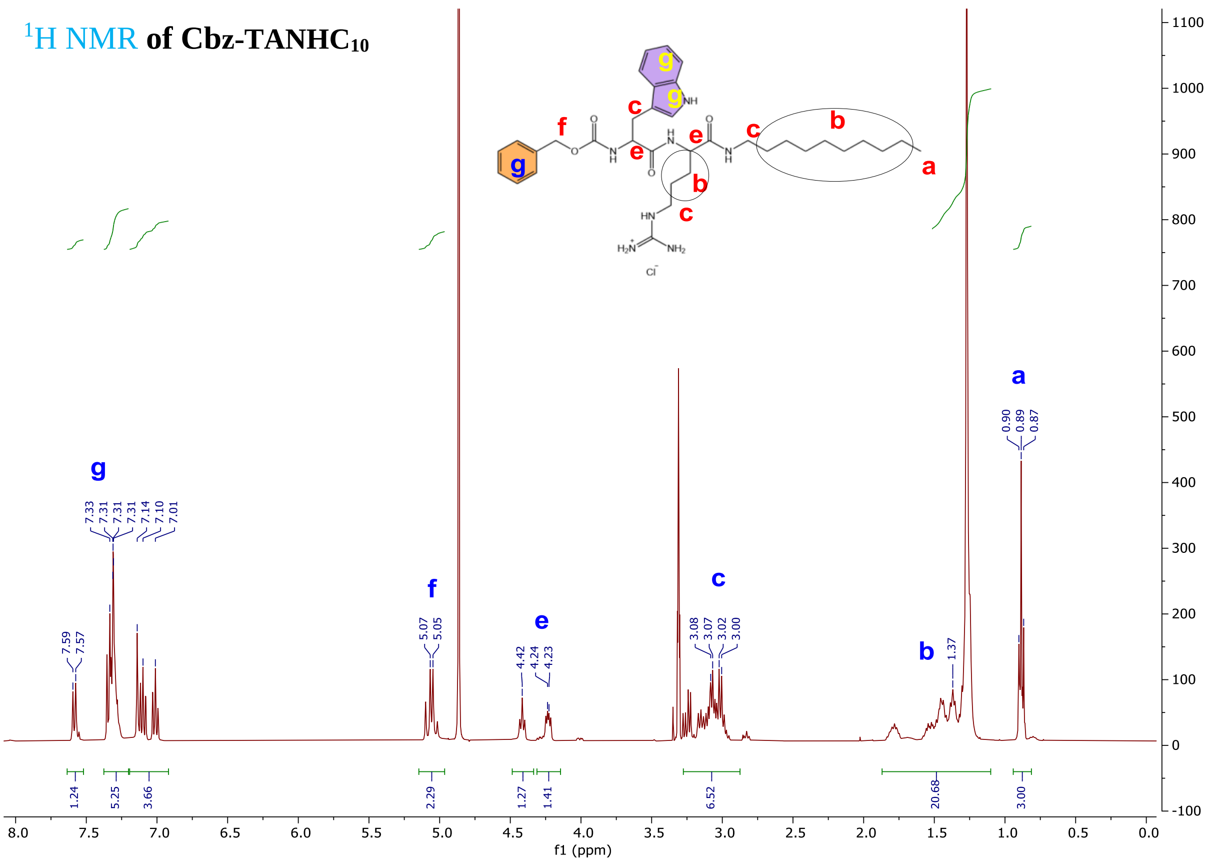

^{13}C NMR **of Cbz-TANHC$_{10}$**

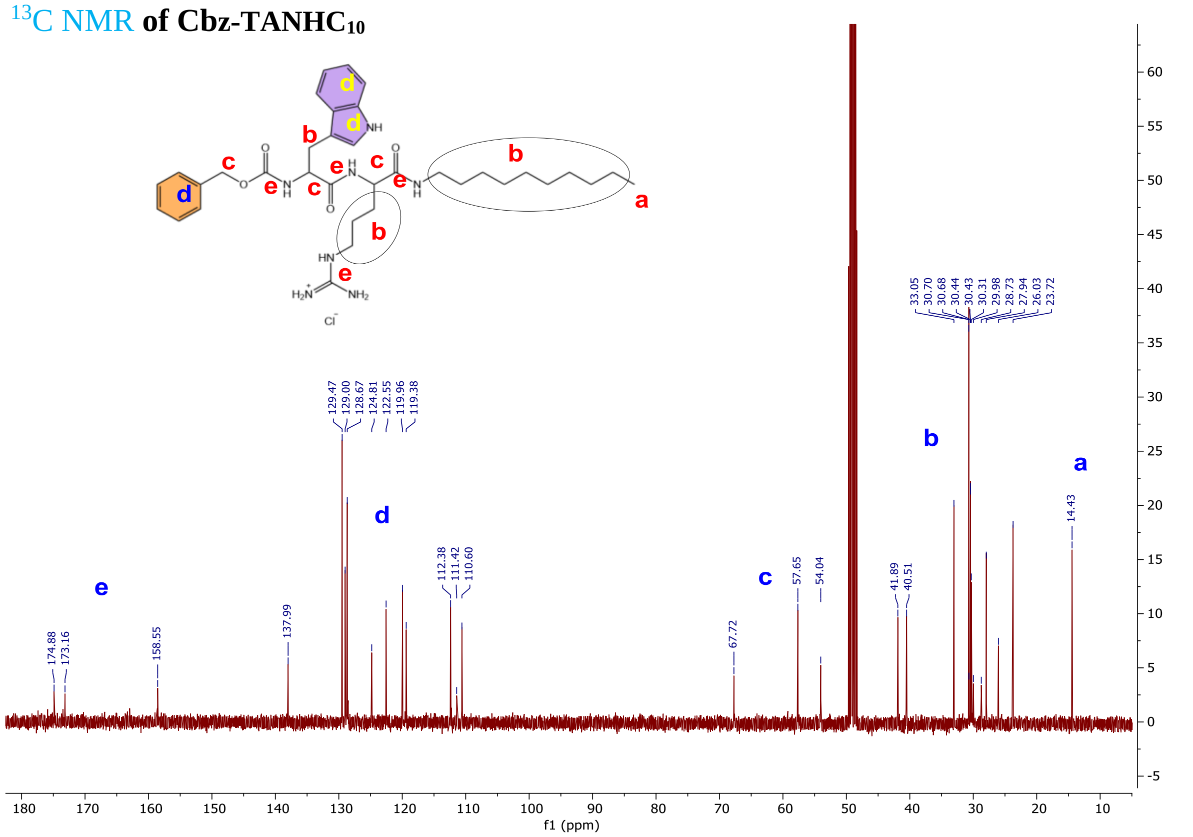

Dept NMR of Cbz-TANHC$_{10}$

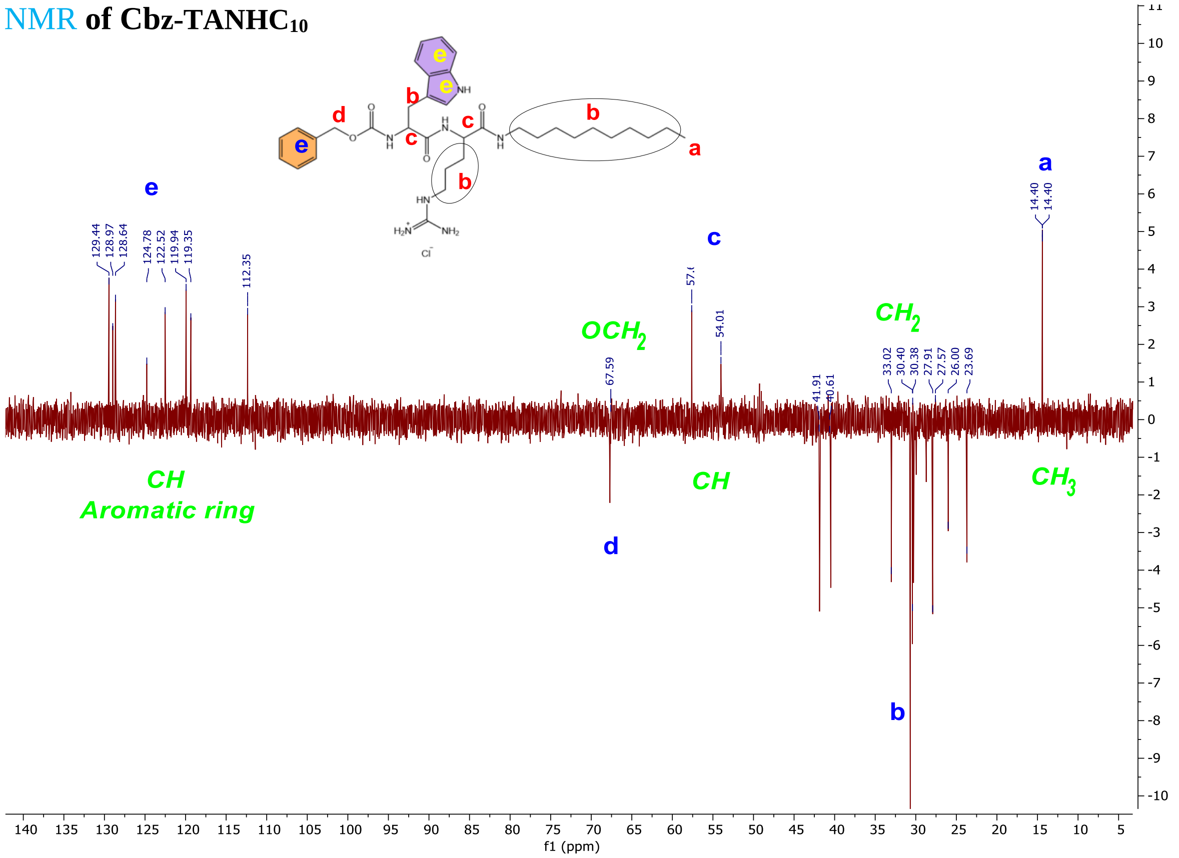

Figure S18. HPLC, ESI-MS, ^{1}H NMR, ^{13}C NMR and Dept of Cbz-TANHC$_{10}$

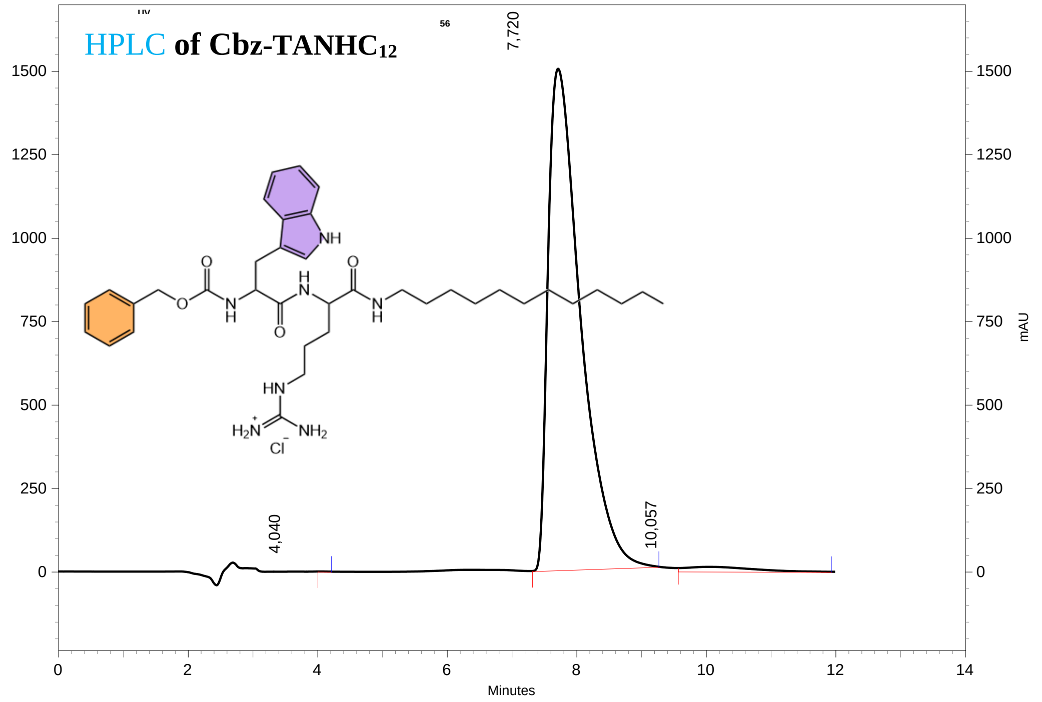

ESI-MS of Cbz-TANHC$_{12}$

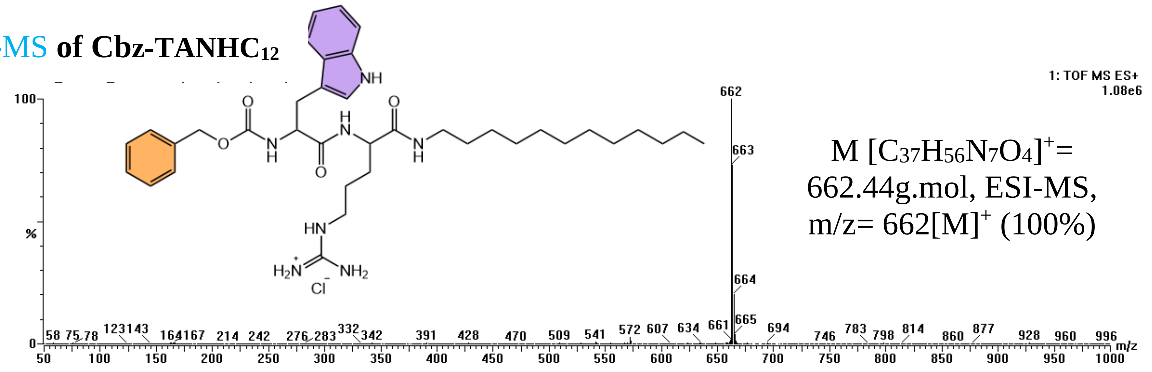

^{1}H NMR of Cbz-TANHC$_{12}$

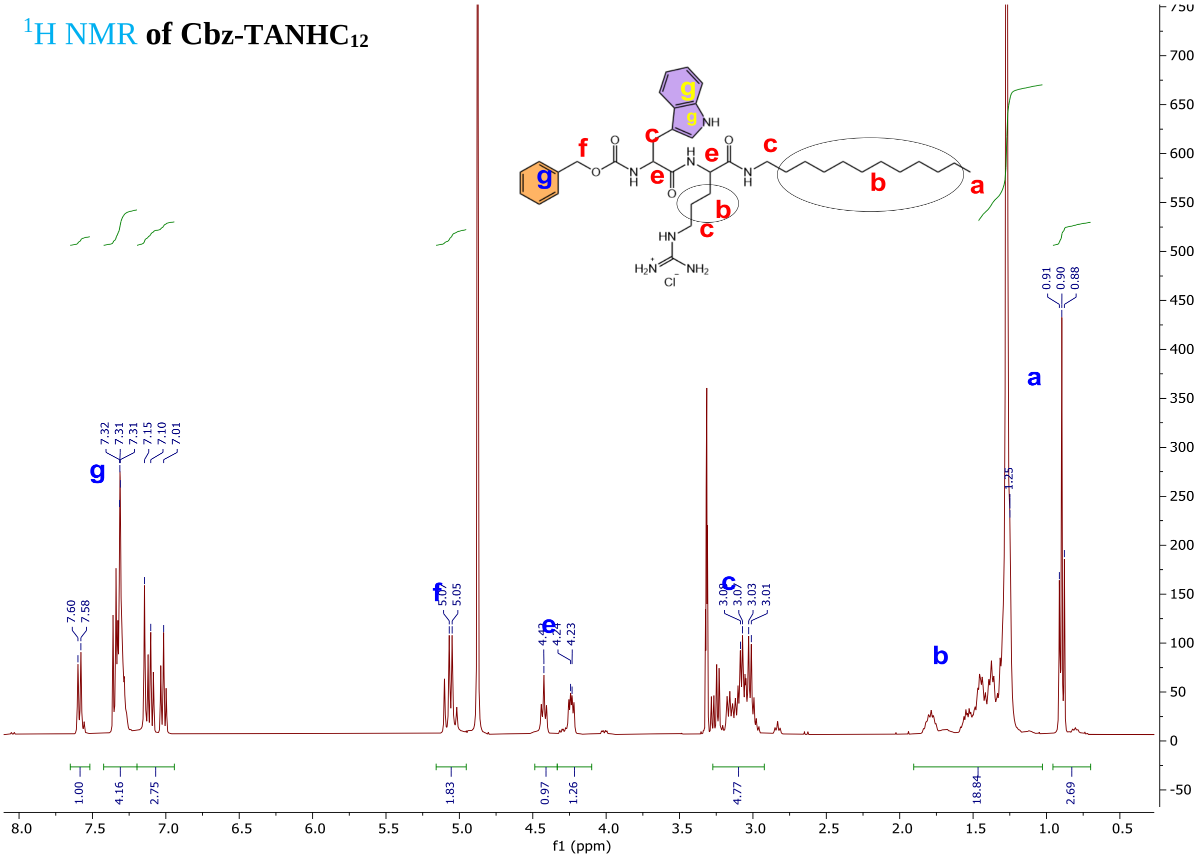

^{13}C NMR of Cbz-TANHC$_{12}$

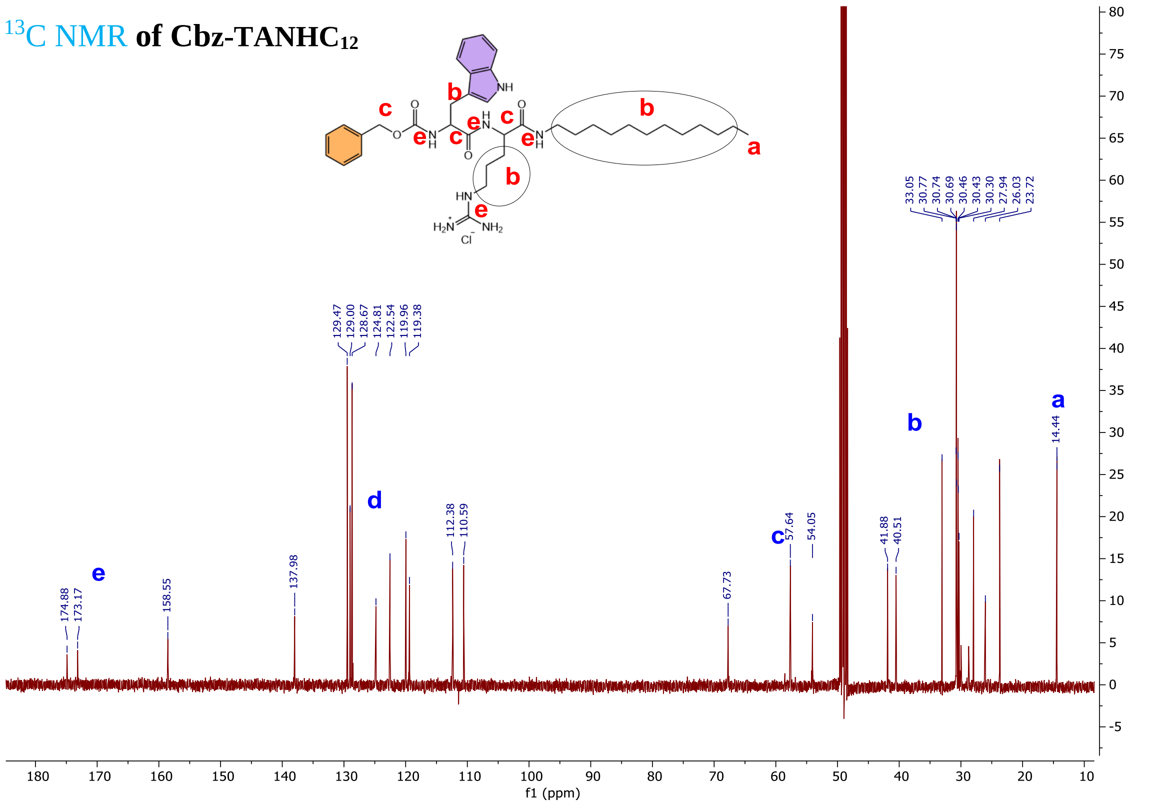

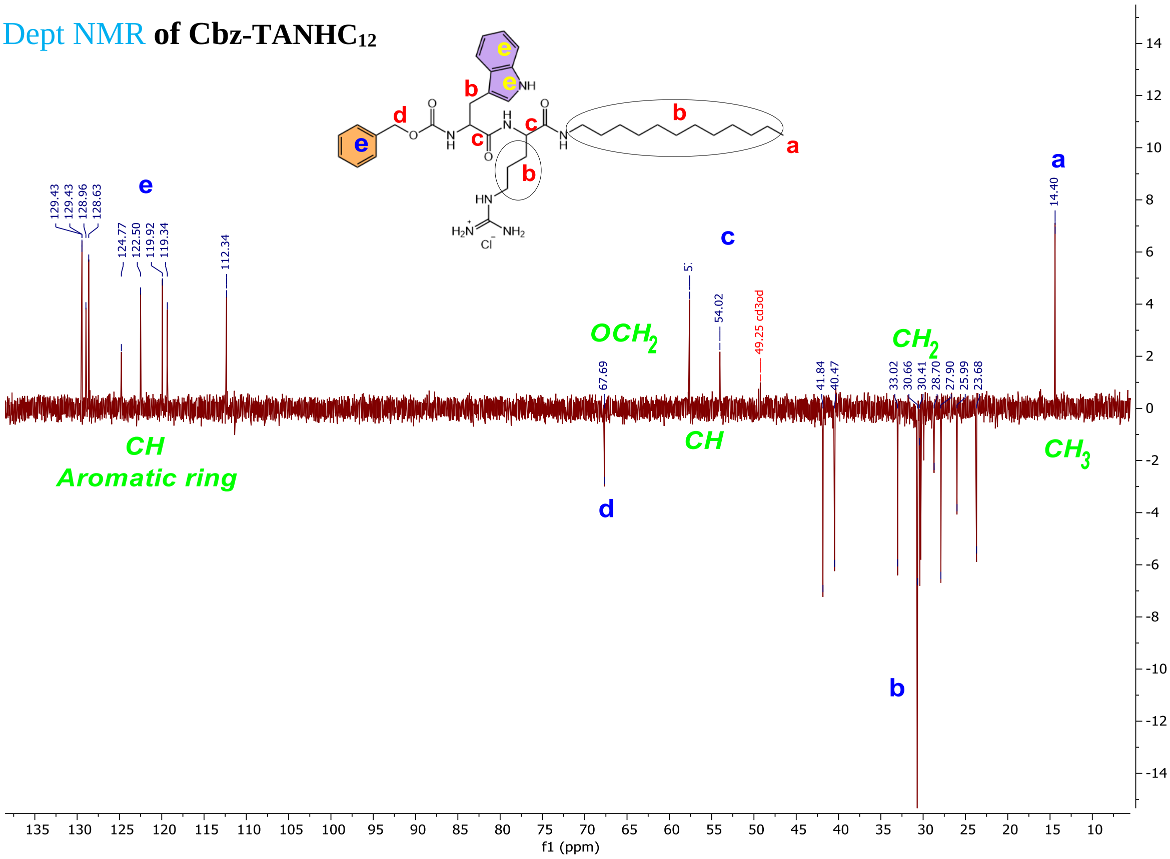

Figure S19. HPLC, ESI-MS, ^{1}H NMR, ^{13}C NMR and Dept of Cbz-TANHC$_{12}$

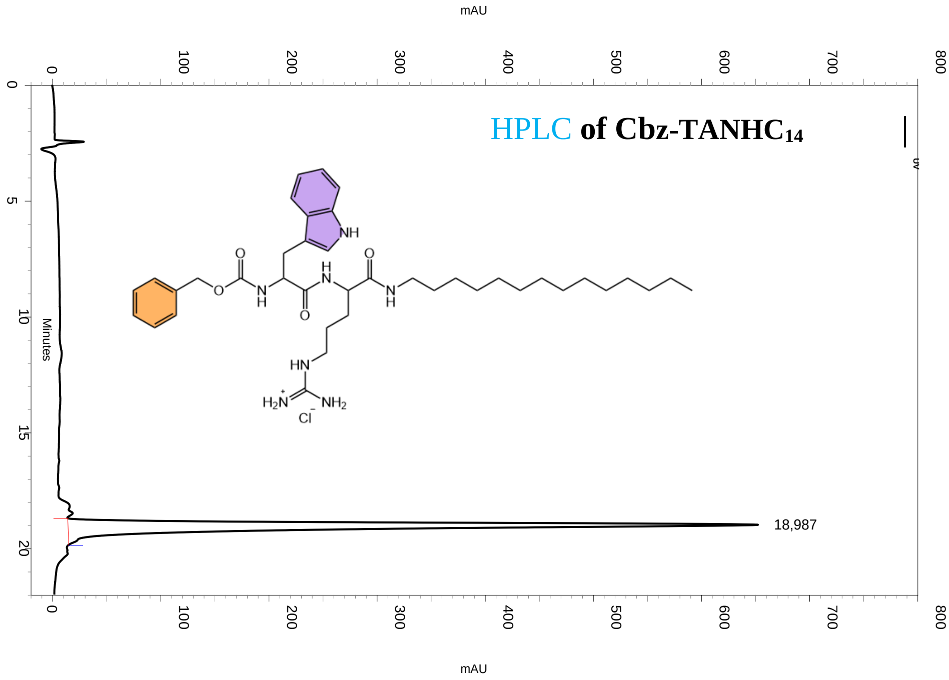
HPLC of Cbz-TANHC$_{14}$
mAU
Minutes
18,987
H$_2$N$^+$ NH$_2$
Cl$^-$

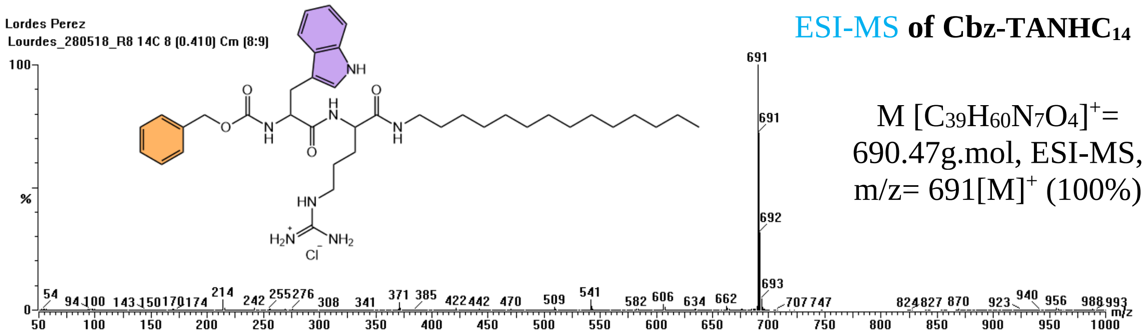
ESI-MS of Cbz-TANHC$_{14}$
Lordes Perez
Lourdes_280518_R8 14C 8 (0.410) Cm (8:9)
M [$C_{39}H_{60}N_7O_4$]$^+$=
690.47g.mol, ESI-MS,
m/z= 691[M]$^+$ (100%)
%
m/z
691
692
693

^{1}H NMR of Cbz-TANHC$_{14}$

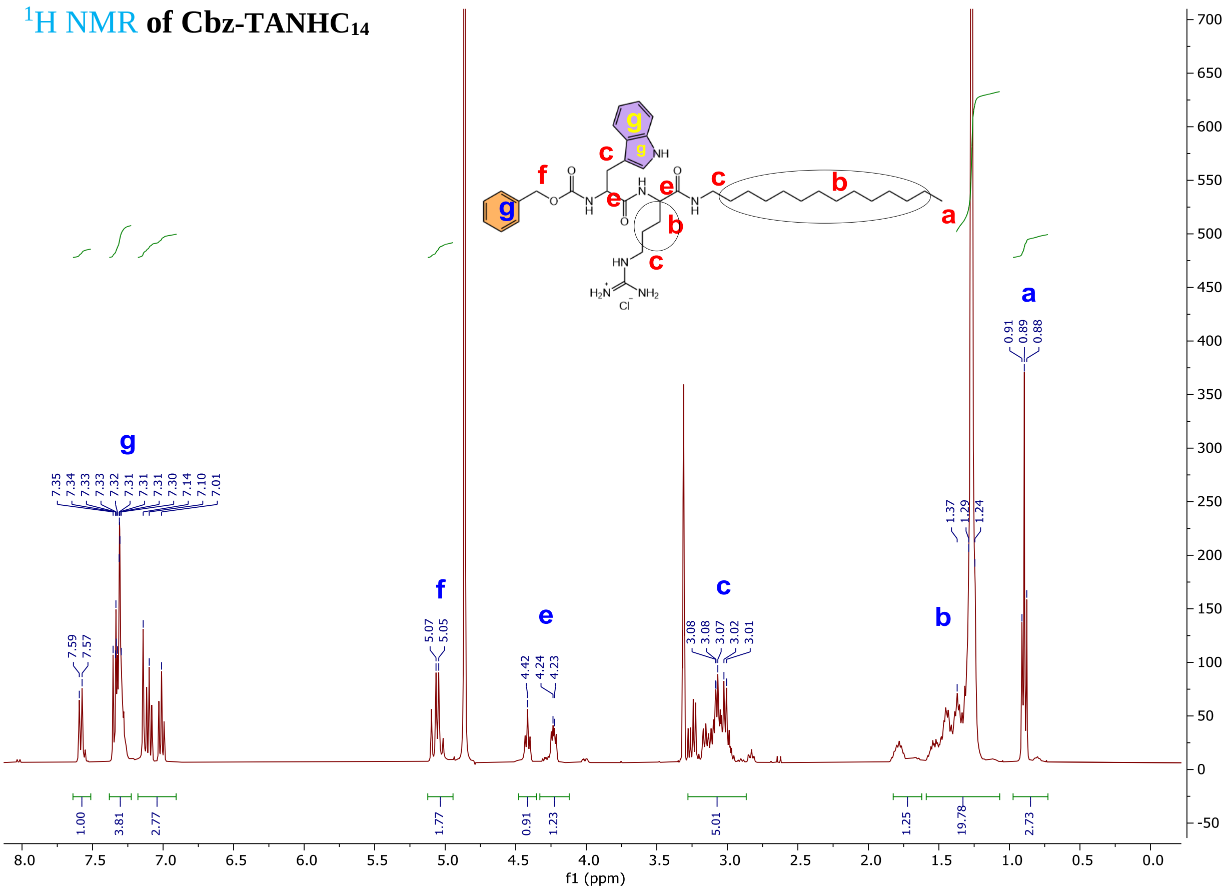

^{13}C NMR of Cbz-TANHC$_{14}$

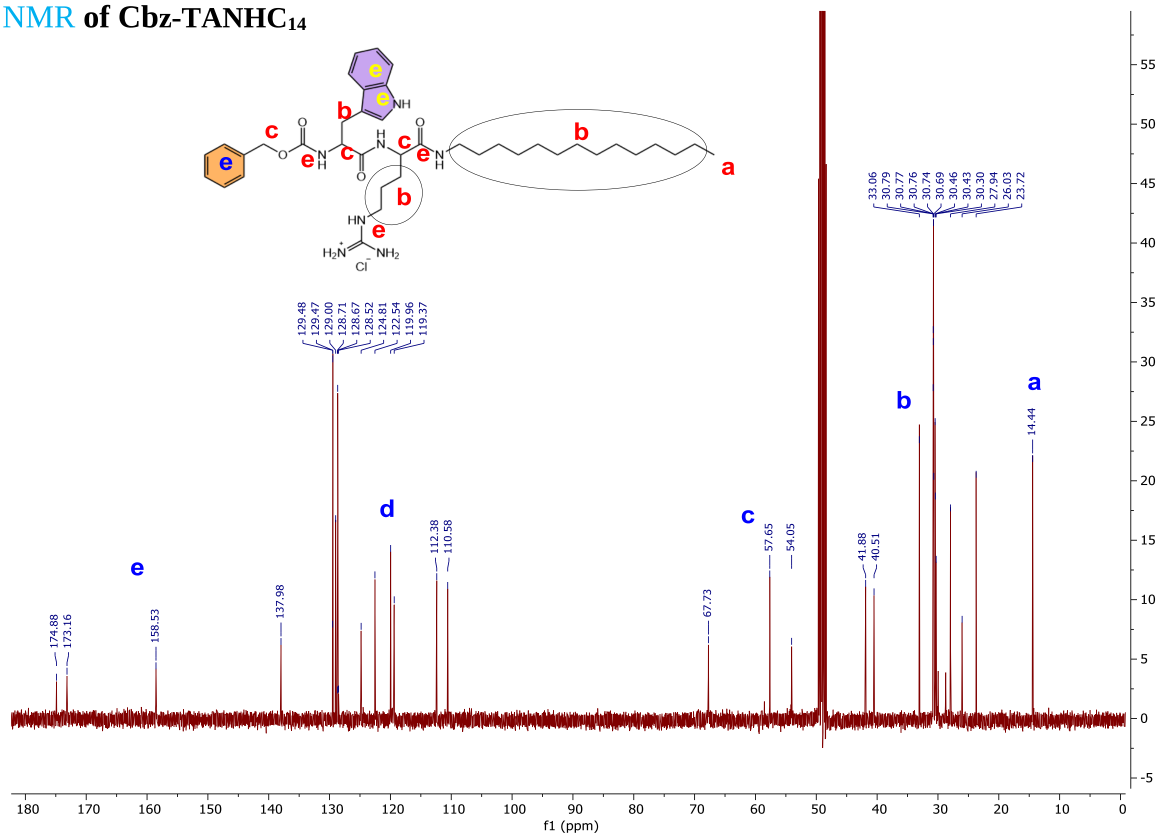

Dept NMR **of Cbz-TANHC$_{14}$**

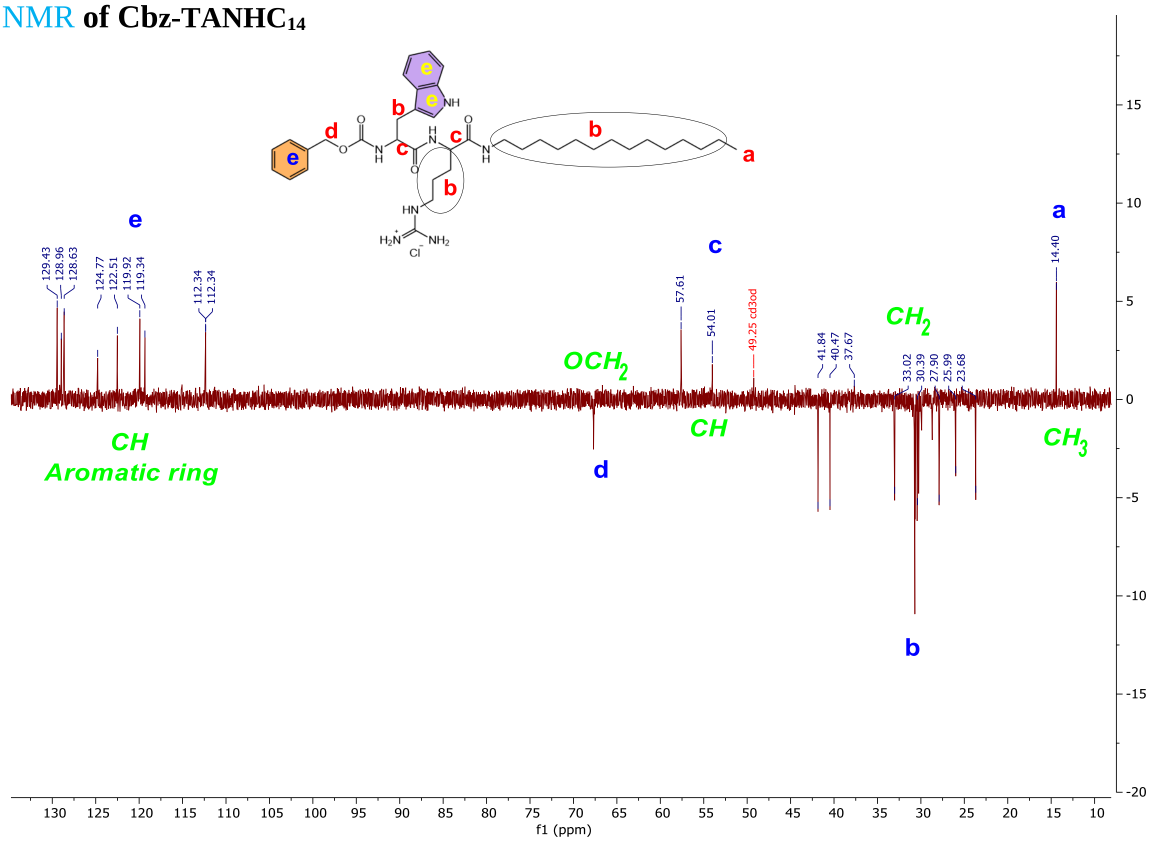

Figure S20. HPLC, ESI-MS, ^{1}H NMR,^{13}C NMR and Dept of Cbz-TANHC$_{14}$

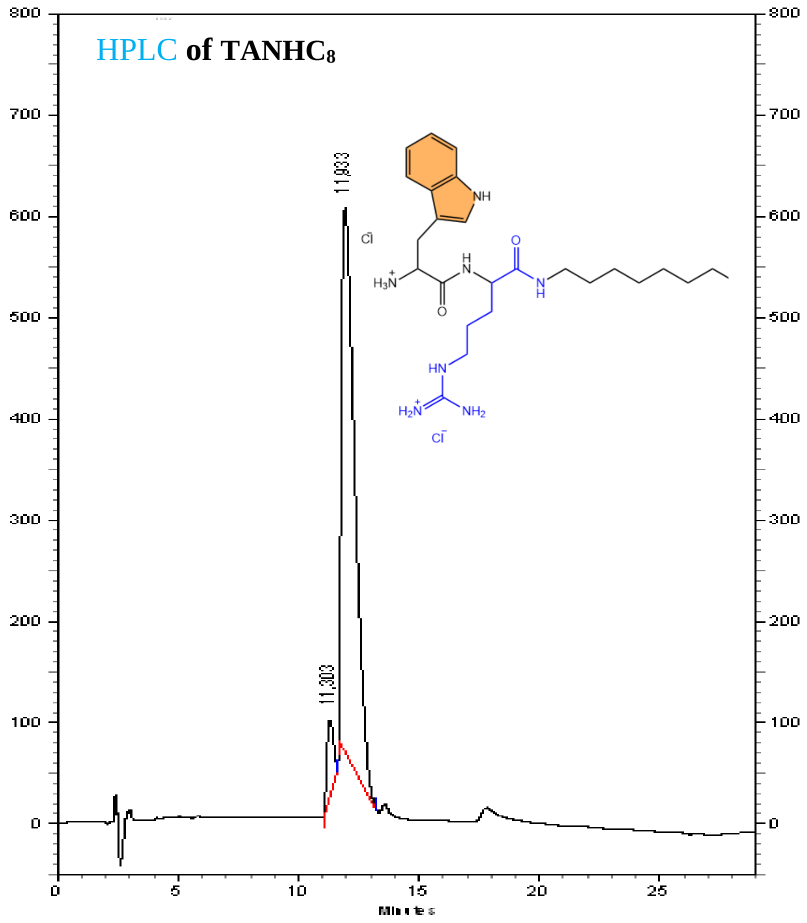

ESI-MS of $TANHC_8$

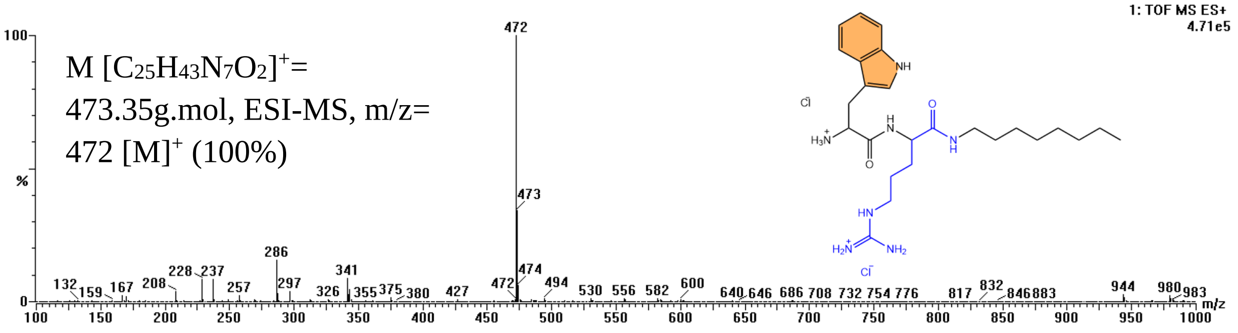

^{1}H NMR of TANHC$_8$

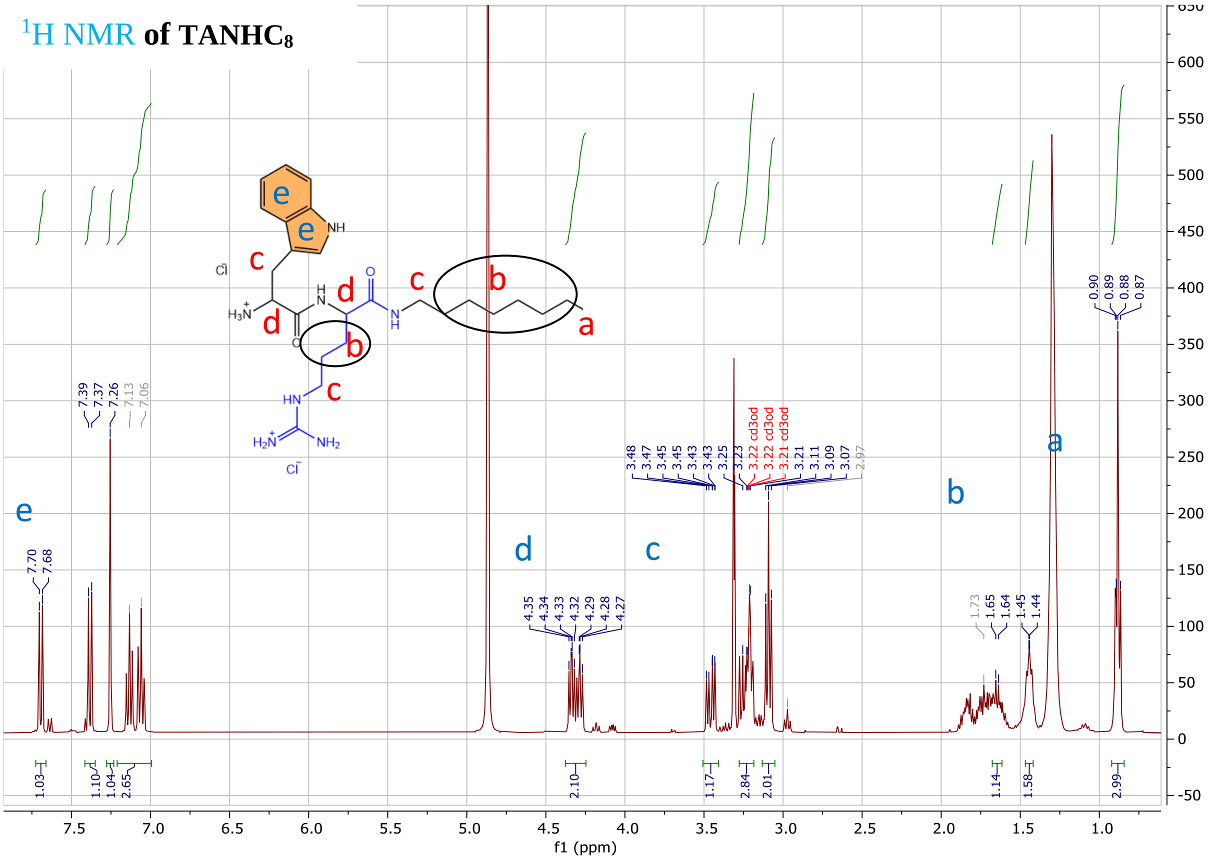

^{13}C NMR of TANHC$_8$

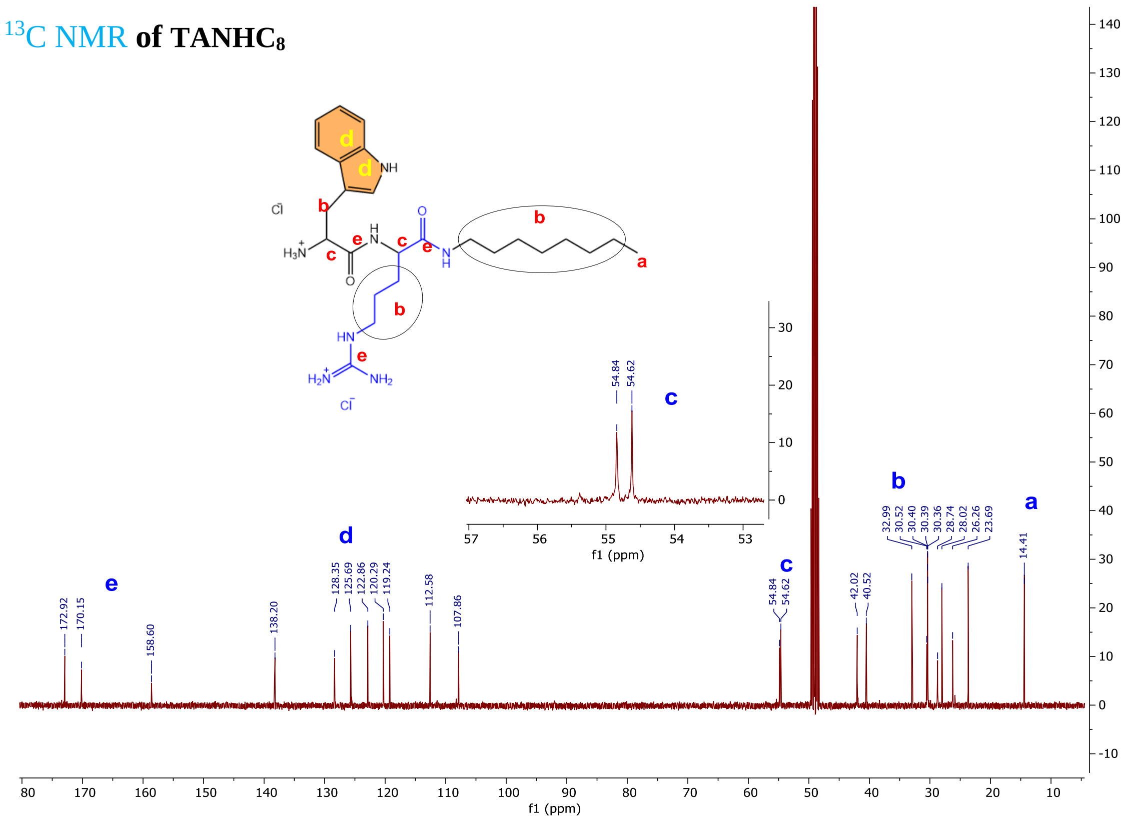

Dept NMR **of TANHC$_8$**

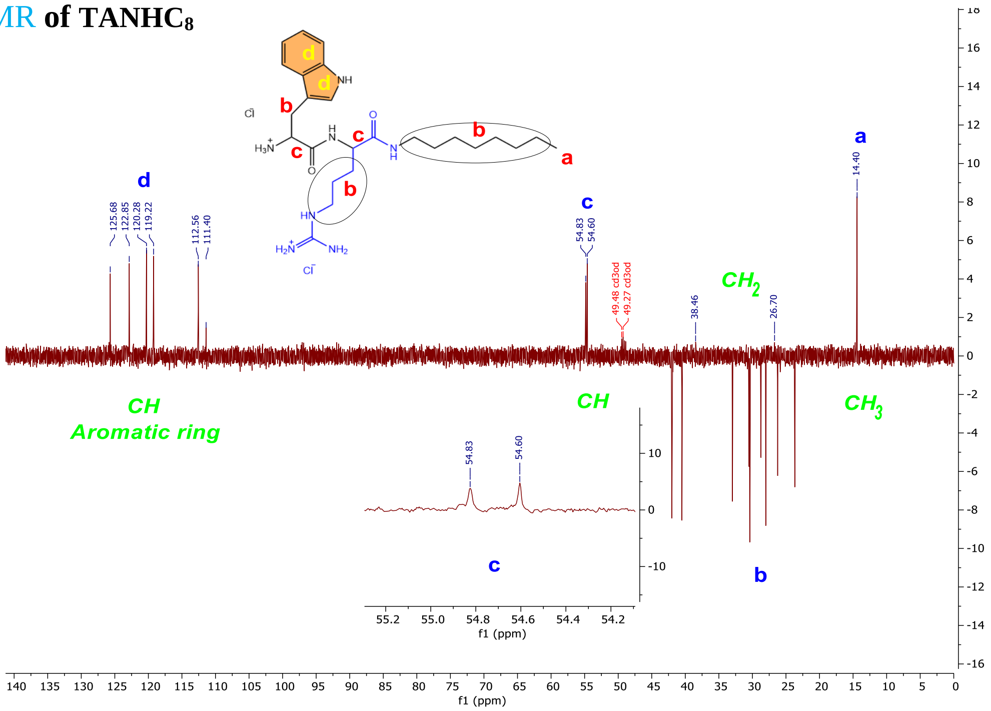

Figure S21. HPLC, ESI-MS, ^{1}H NMR, ^{13}C NMR and Dept of TANHC$_8$

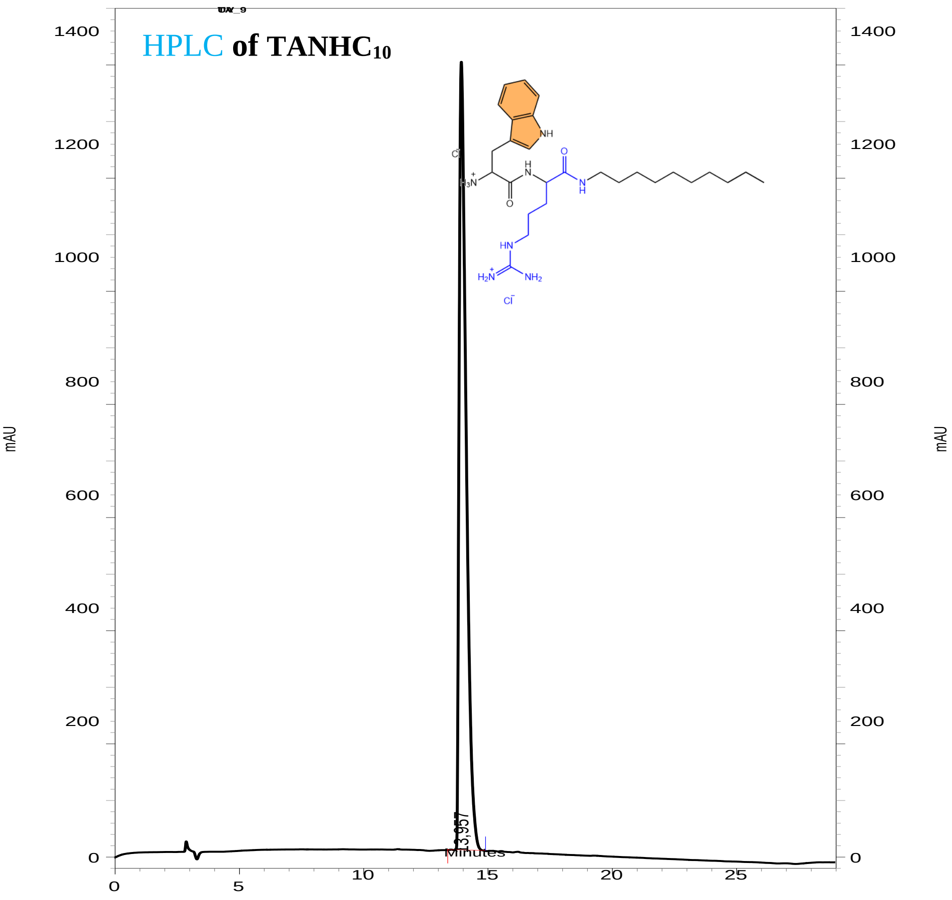

ESI-MS of TANHC$_{10}$

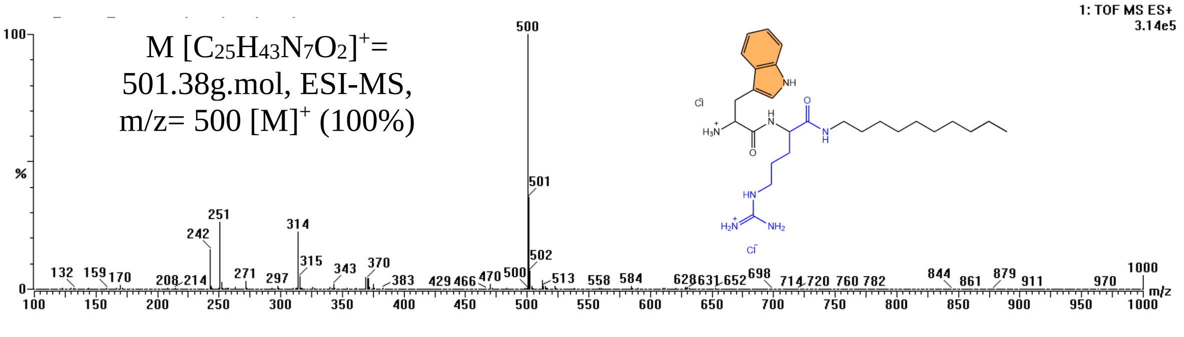

^{1}H NMR of **TANHC$_{10}$**

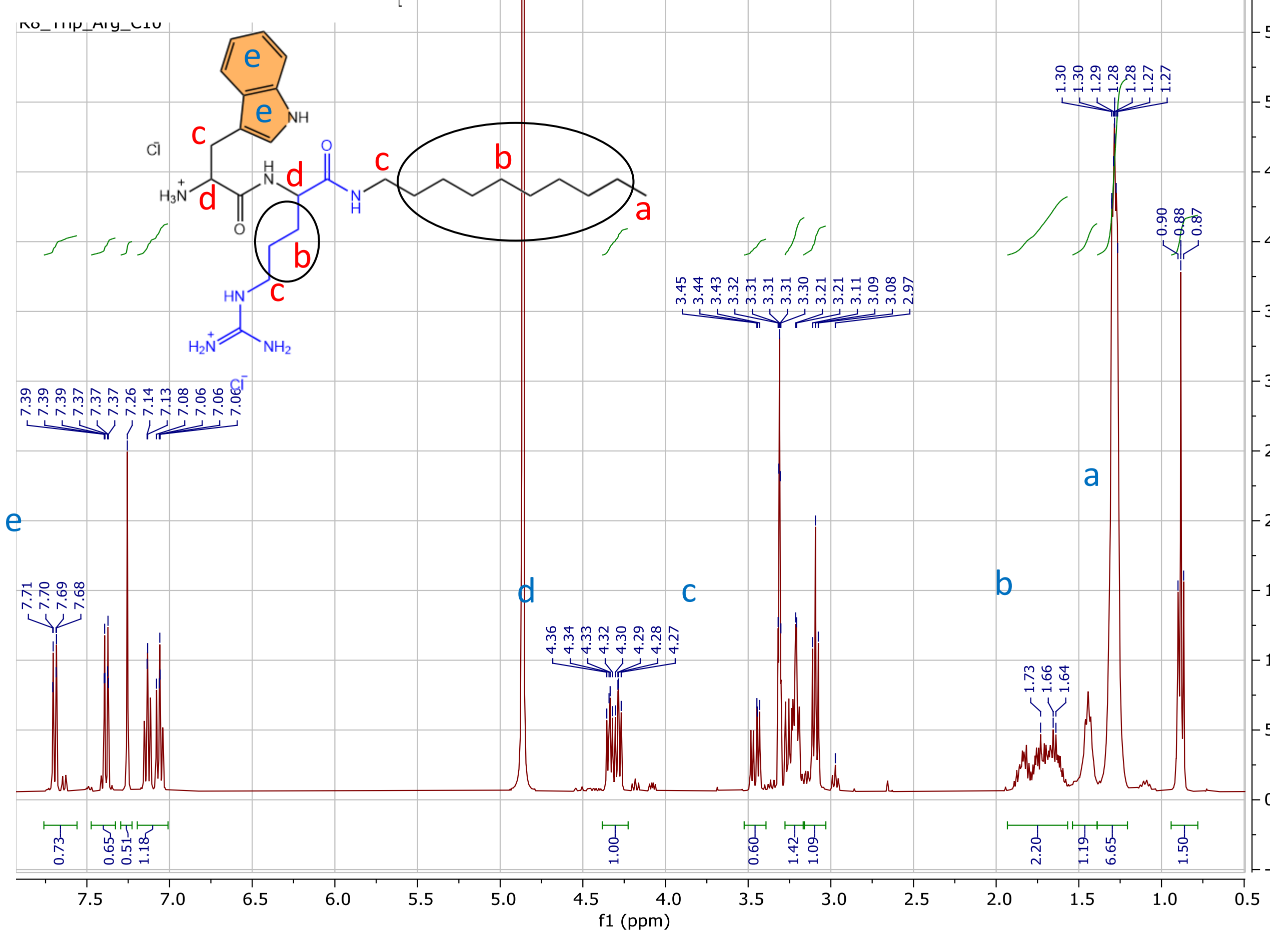

^{13}C NMR of **TANHC$_{10}$**

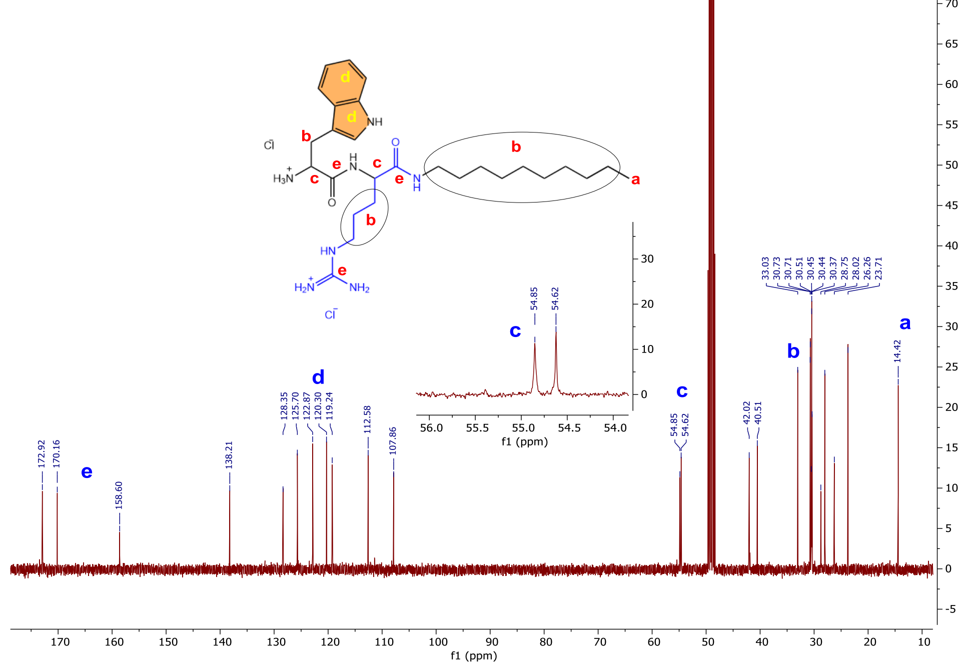

Dept NMR **of TANHC$_{10}$**

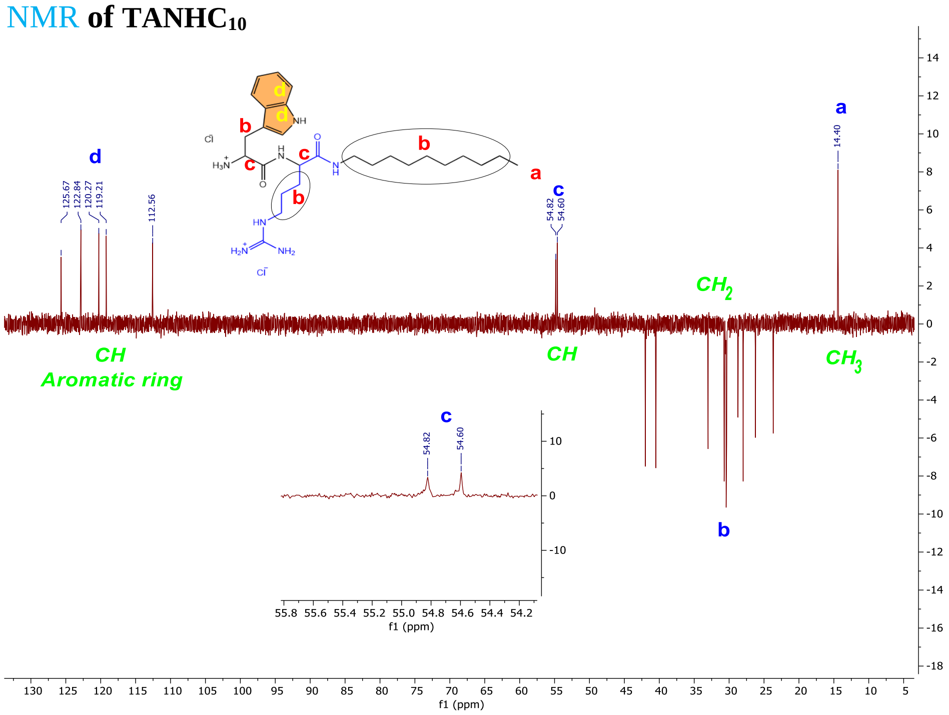

Figure S22. HPLC, ESI-MS, ^{1}H NMR, ^{13}C NMR and Dept of TANHC$_{10}$

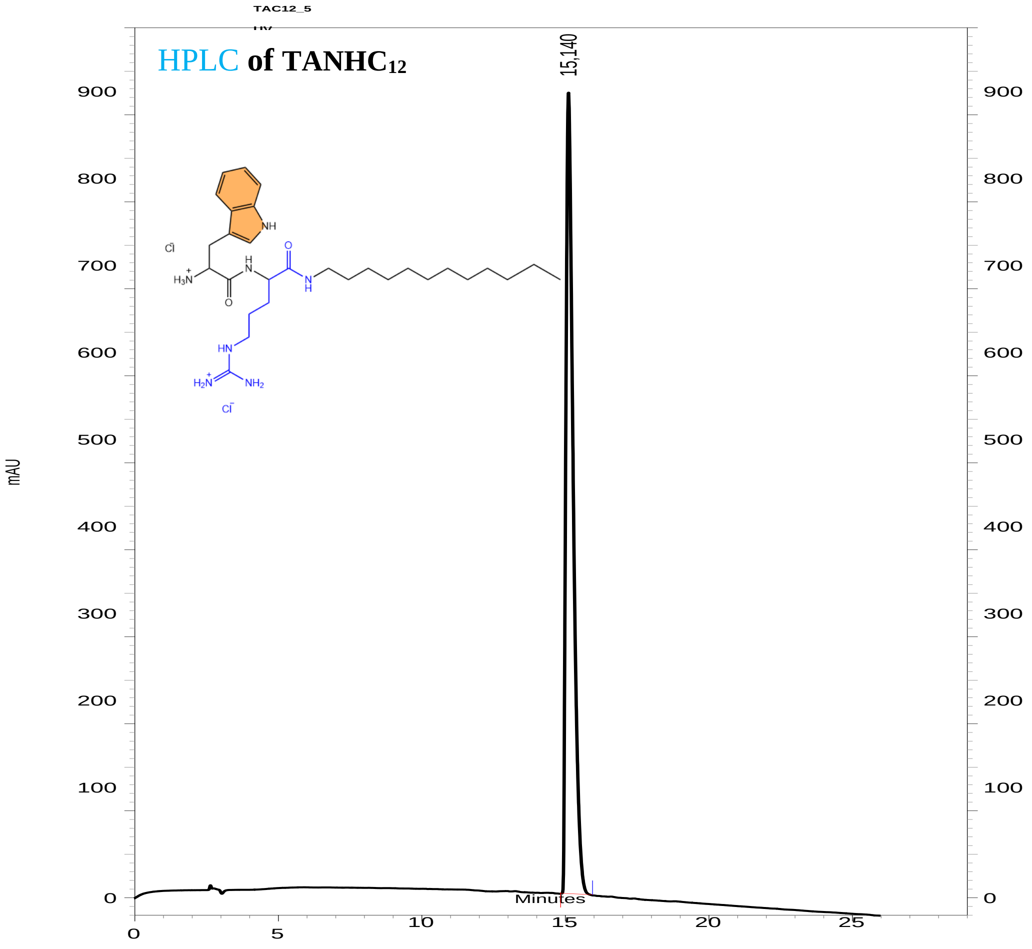

ESI-MS of TANHC12

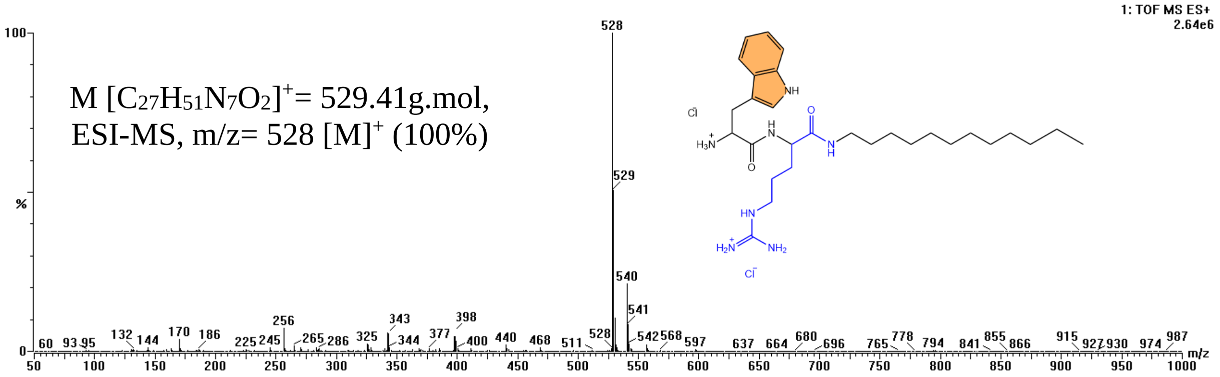

^{1}H NMR of TANHC$_{12}$

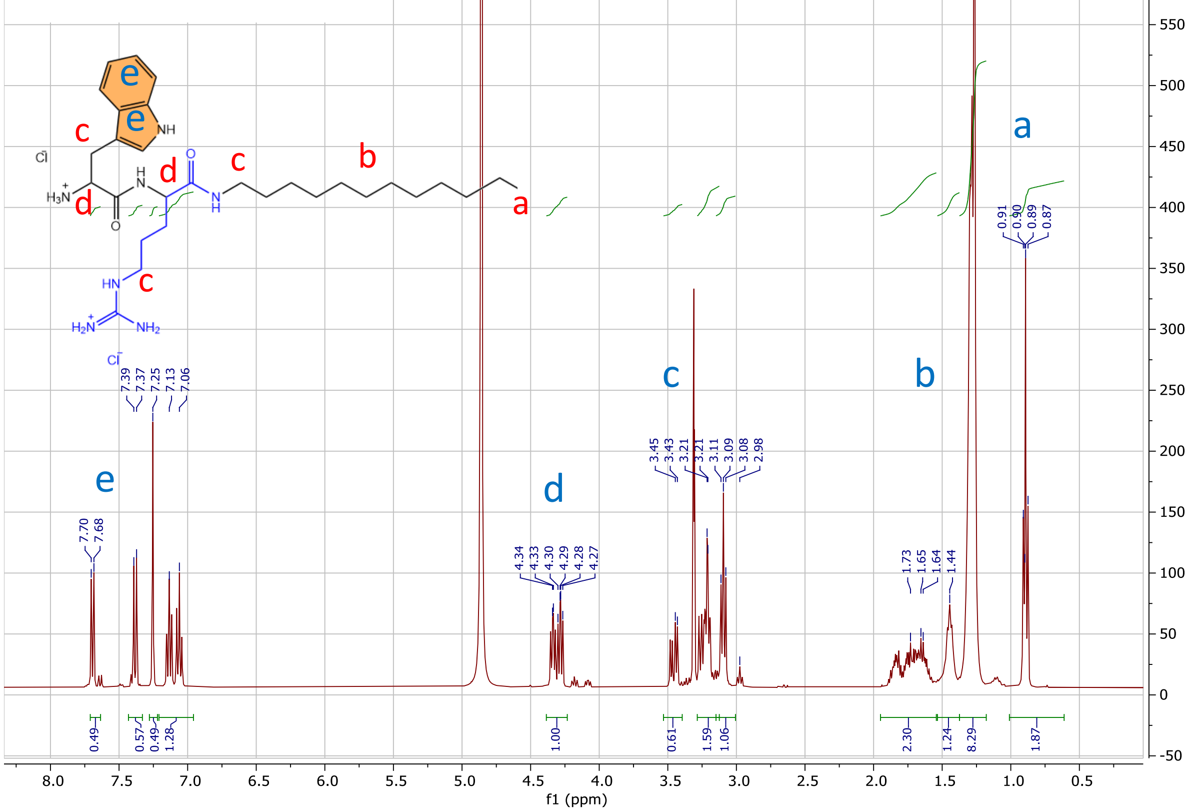

^{13}C NMR of TANHC$_{12}$

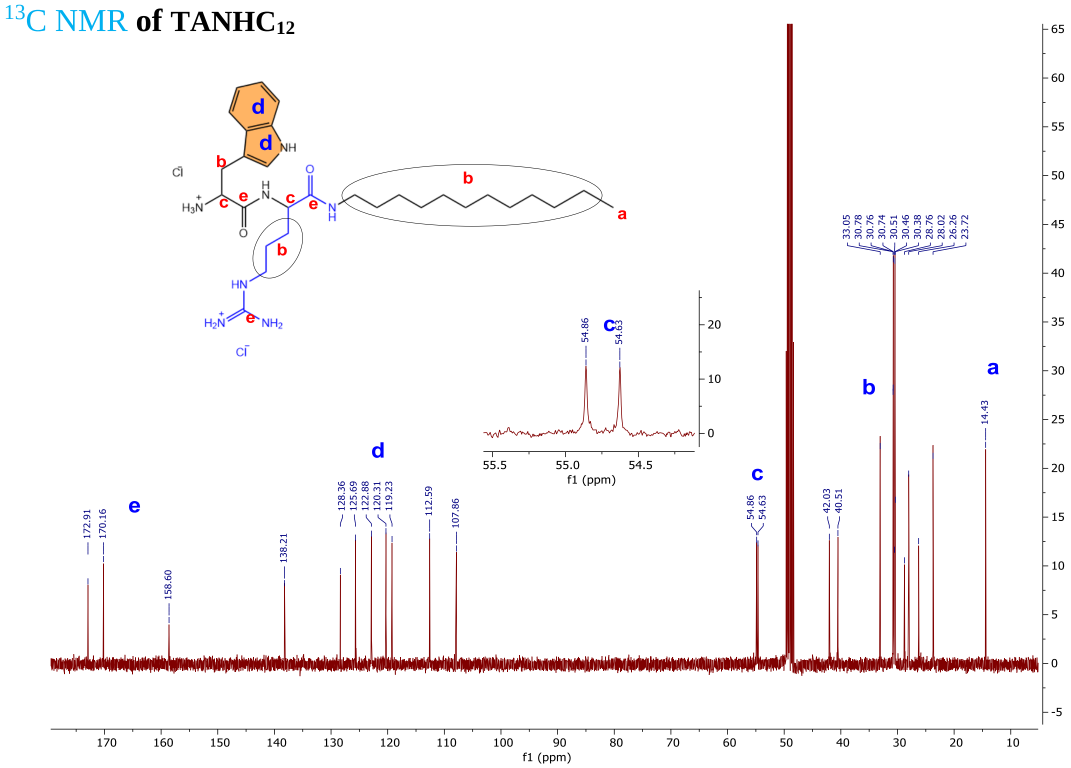

Dept NMR **of TANHC$_{12}$**

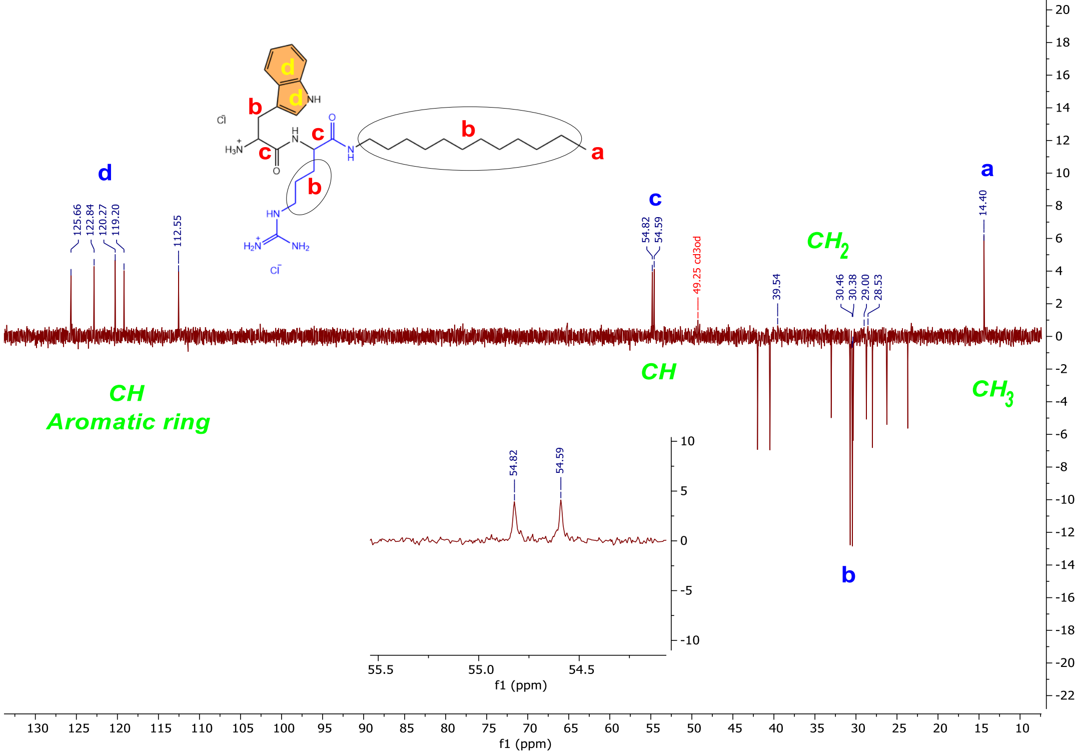

Figure S23. HPLC, ESI-MS, ^{1}H NMR, ^{13}C NMR and Dept of TANHC$_{12}$

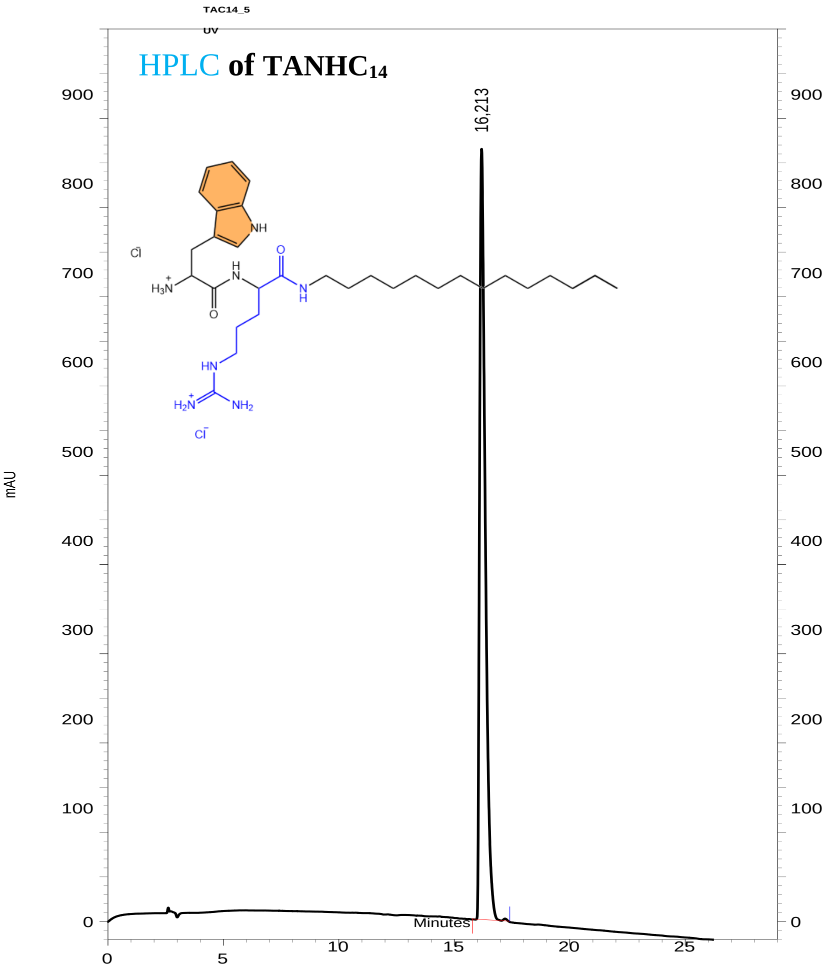

ESI-MS of $TANHC_{14}$

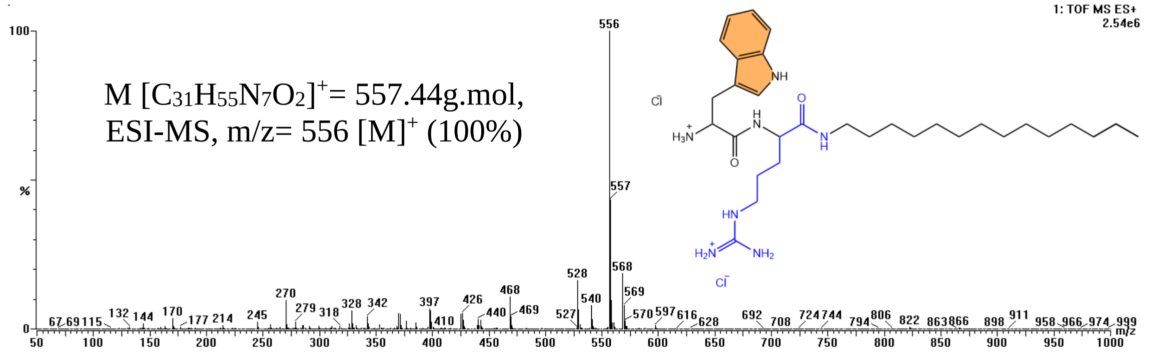

^{1}H NMR of TANHC$_{14}$

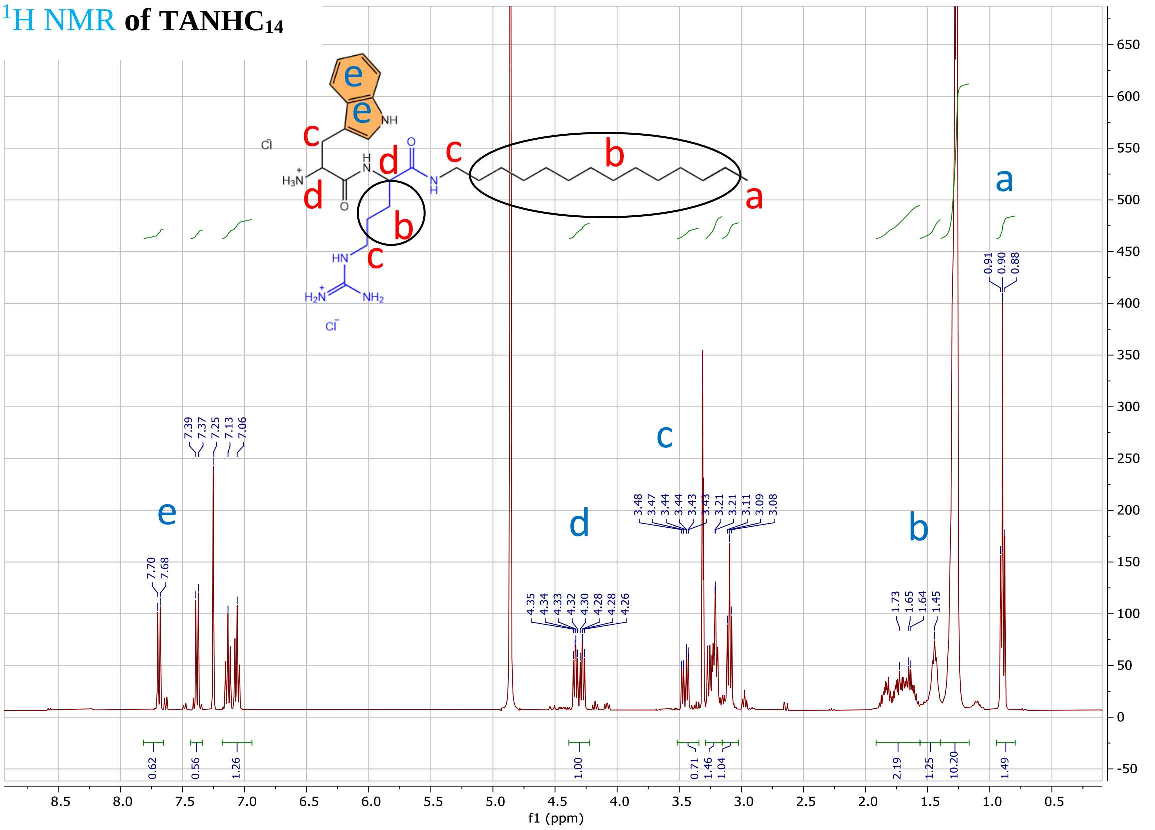

^{13}C NMR of TANHC$_{14}$

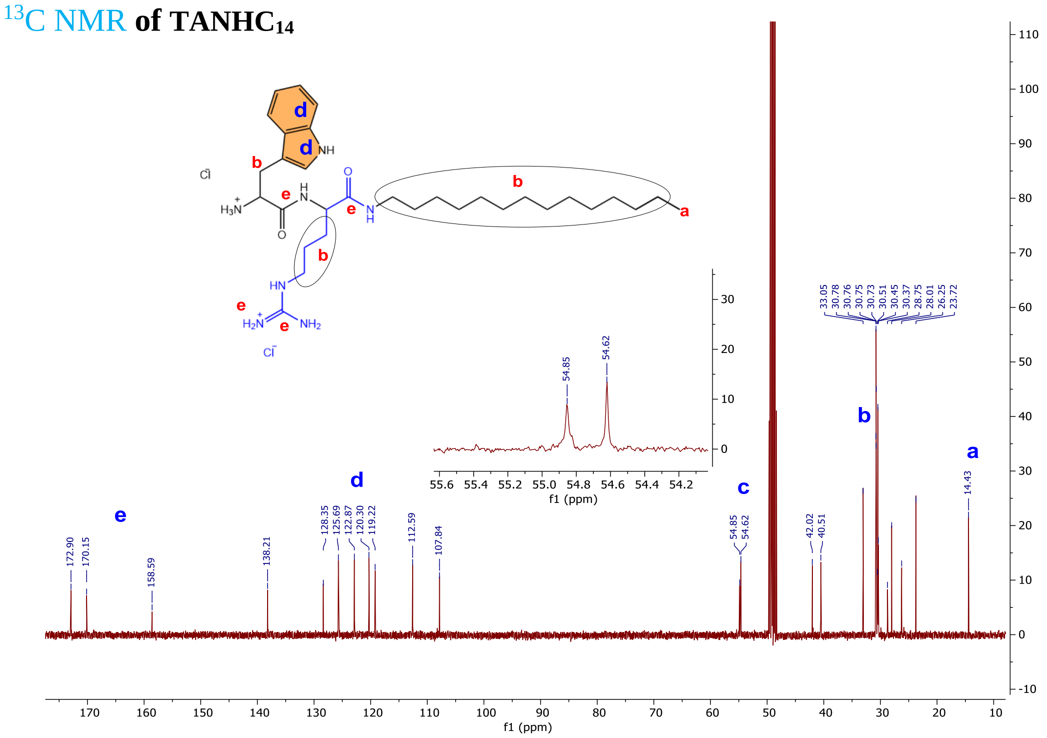

Dept NMR **of TANHC14**

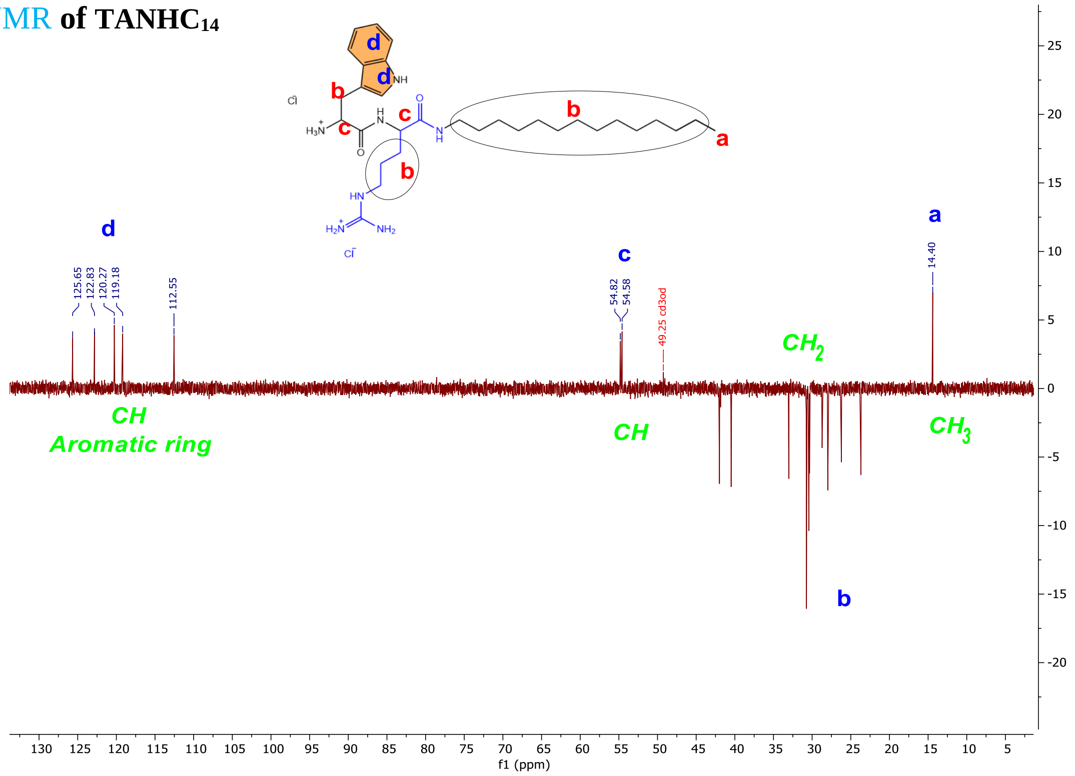

Figure S24. HPLC, ESI-MS, ^{1}H NMR, ^{13}C NMR and Dept of TANHC14

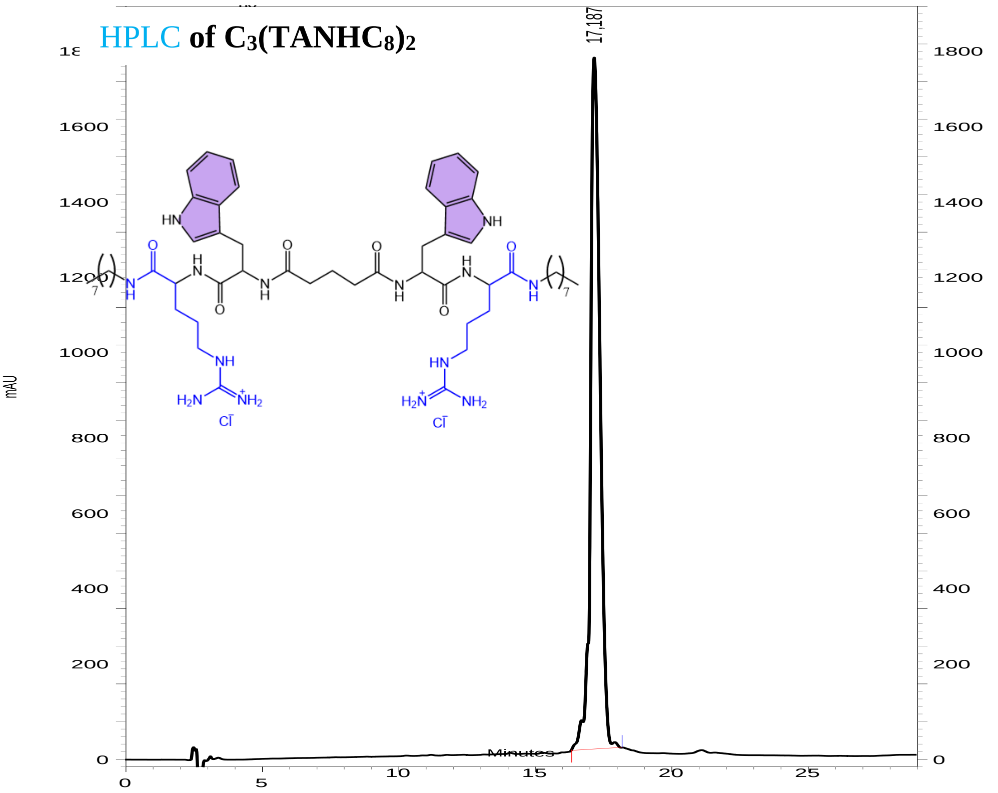

HPLC of $C_3(TANHC_8)_2$
17.187
mAU
1800
1600
1400
1200
1000
800
600
400
200
0
Minutes
0
5
10
15
20
25

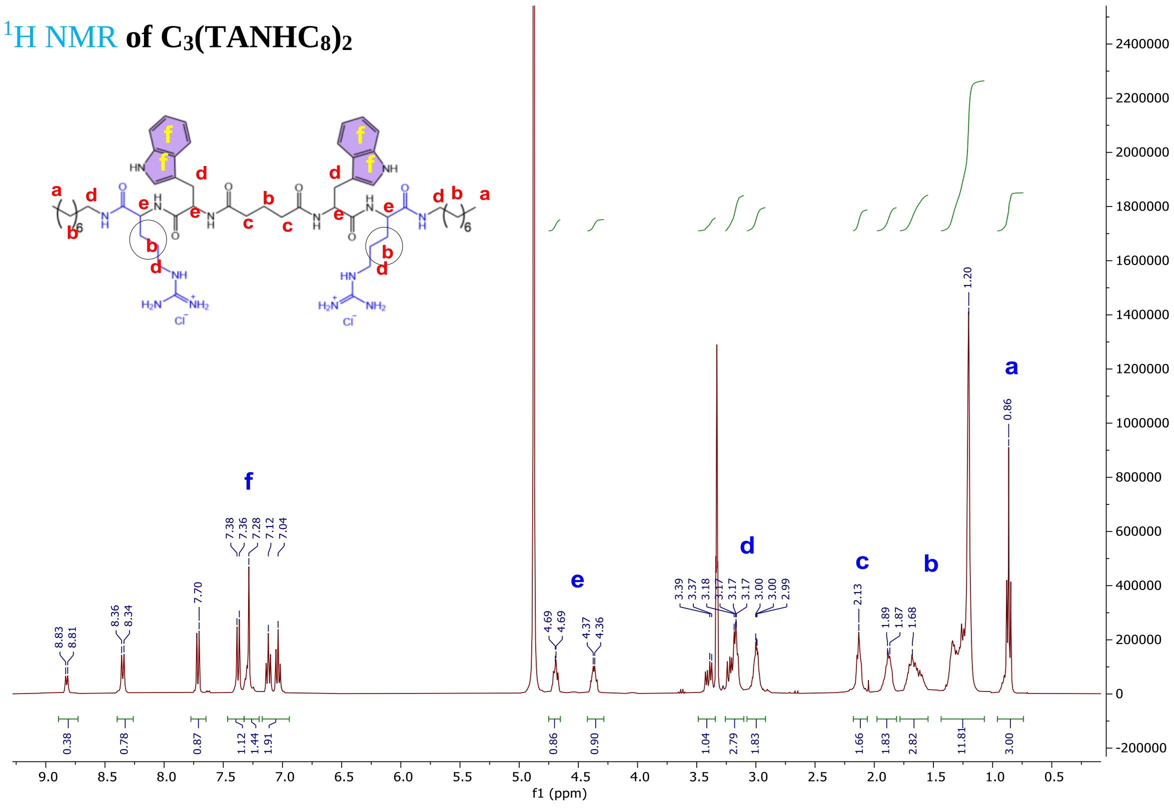

^{1}H NMR of $C_3(TANHC_8)_2$
f
e
d
c
b
a
f1 (ppm)

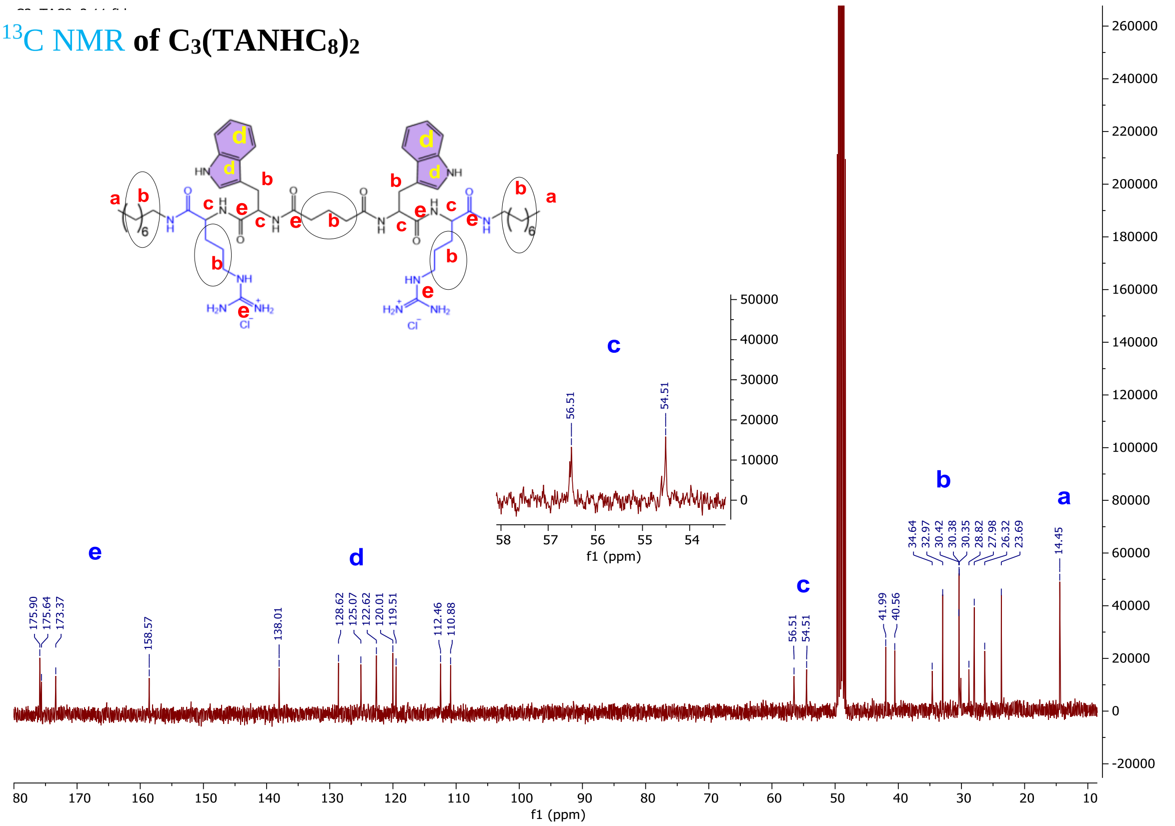

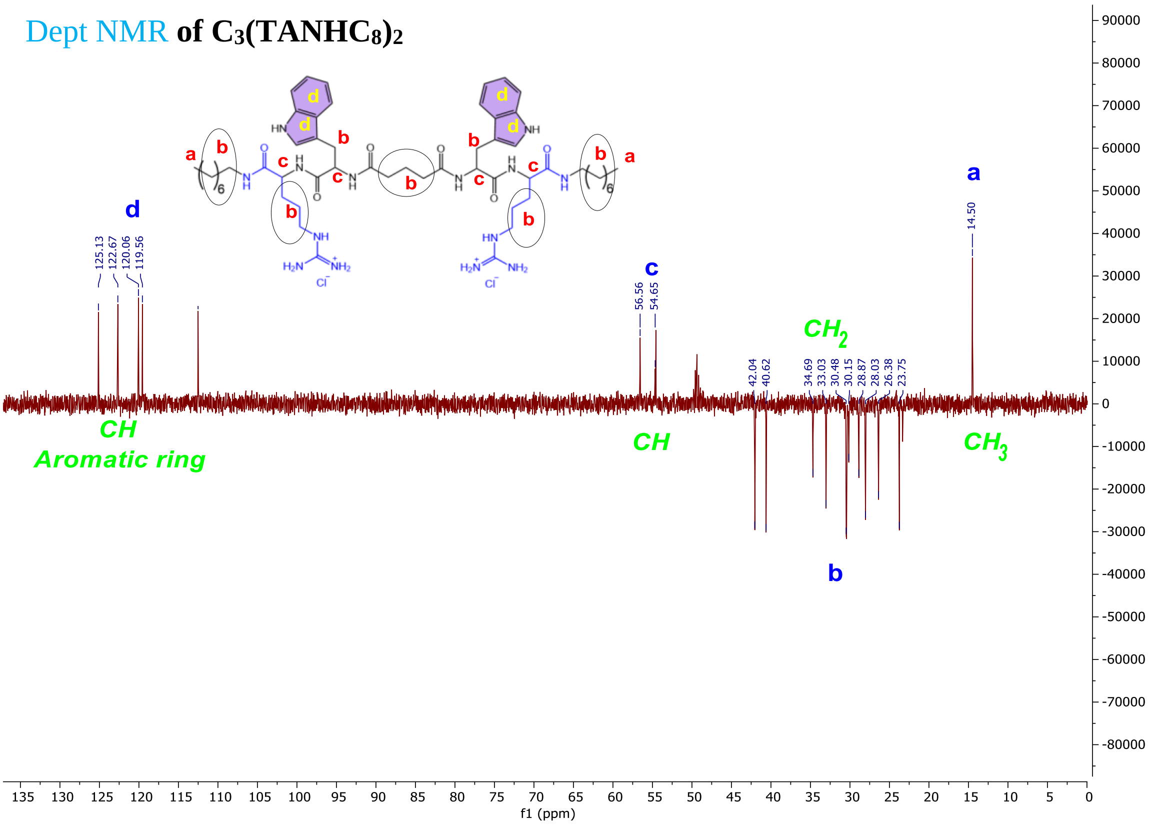

Figure S25. HPLC, ^{1}H NMR, ^{13}C NMR and Dept of $C_3(TANHC_8)_2$

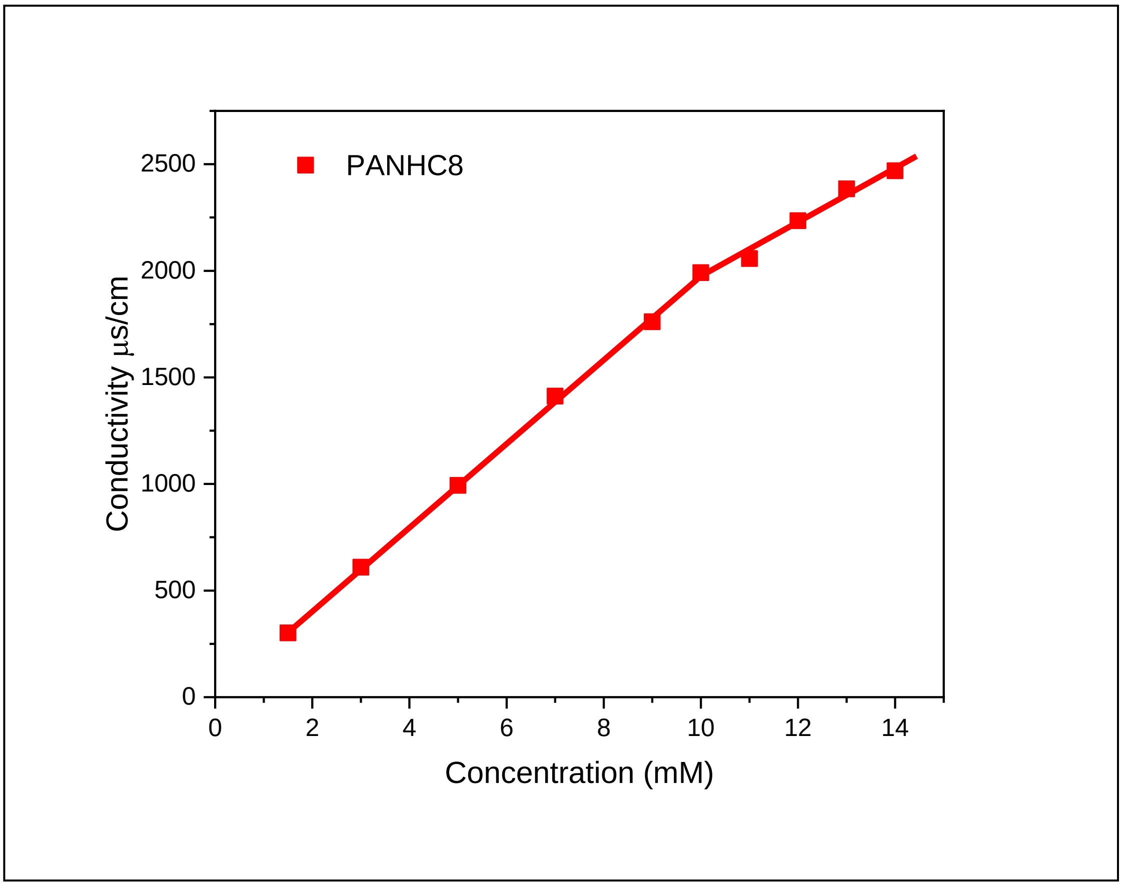
PANHC8
Conductivity µs/cm
2500
2000
1500
1000
500
0
0
2
4
6
8
10
12
14
Concentration (mM)

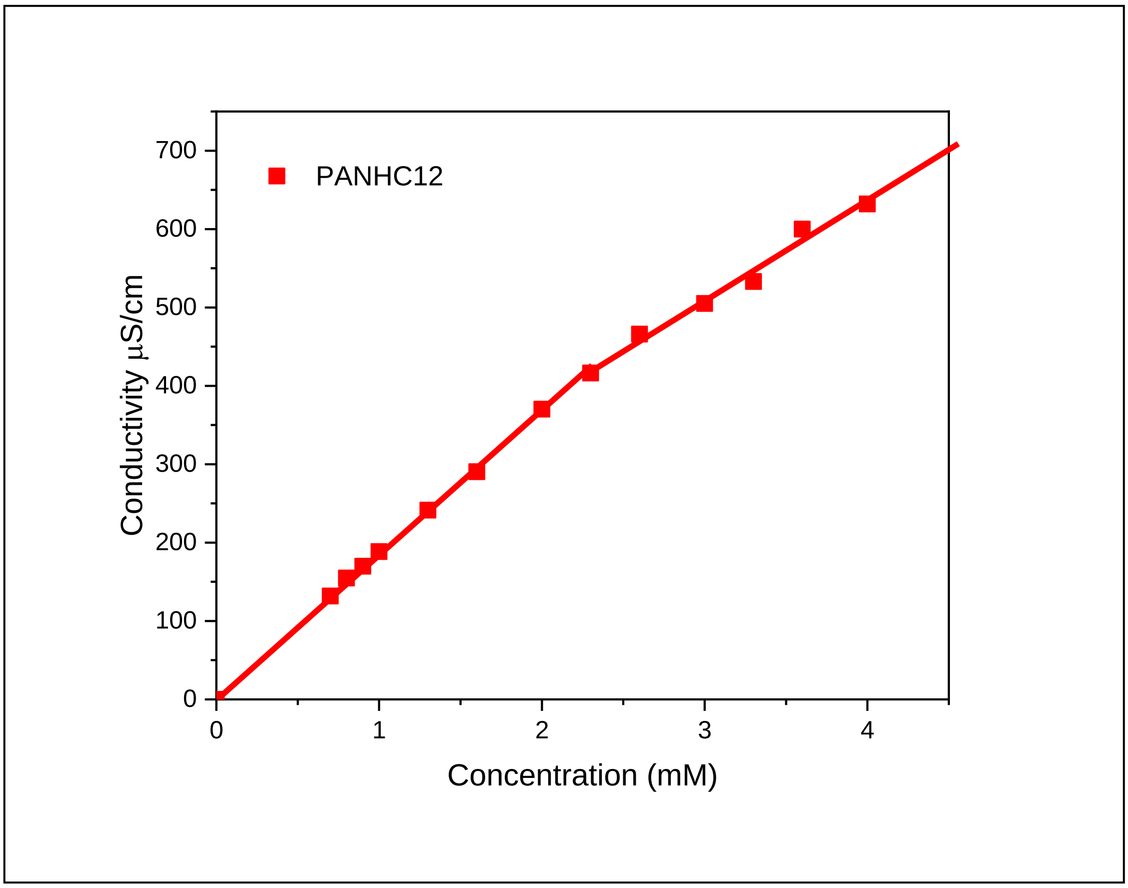
PANHC12
Conductivity µS/cm
700
600
500
400
300
200
100
0
0
1
2
3
4
Concentration (mM)

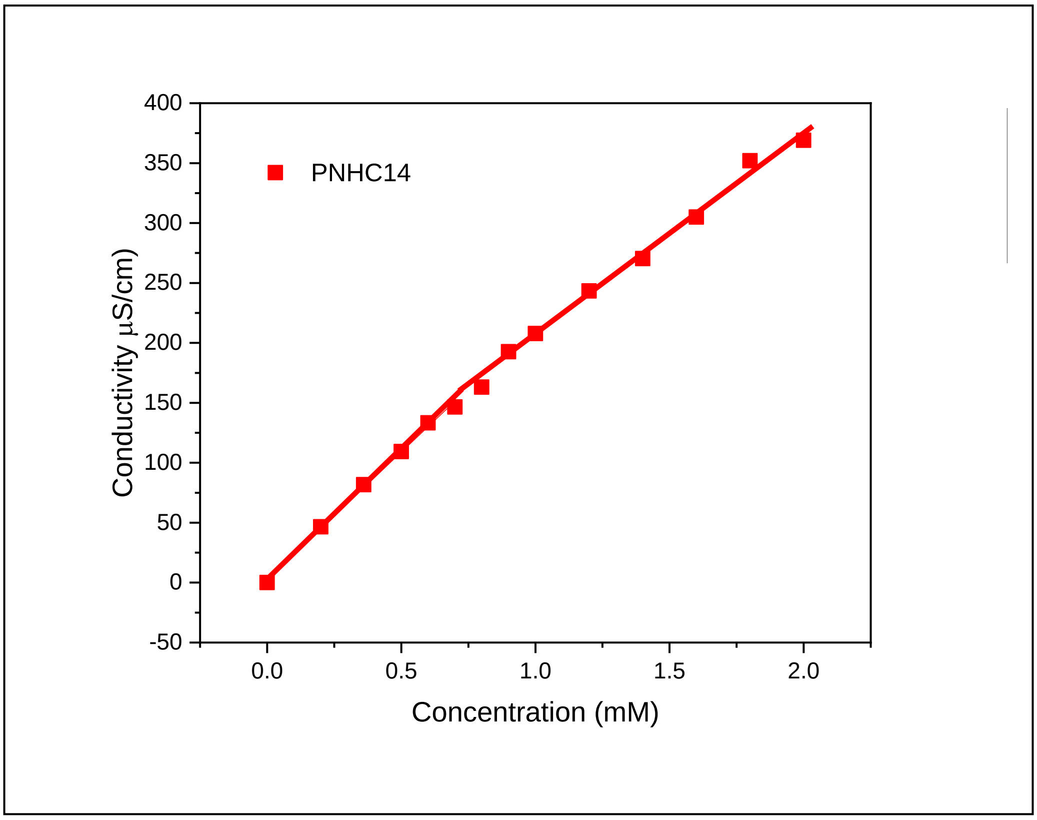
PNHC14
Conductivity µS/cm)
Concentration (mM)
400
350
300
250
200
150
100
50
0
-50
0.0
0.5
1.0
1.5
2.0

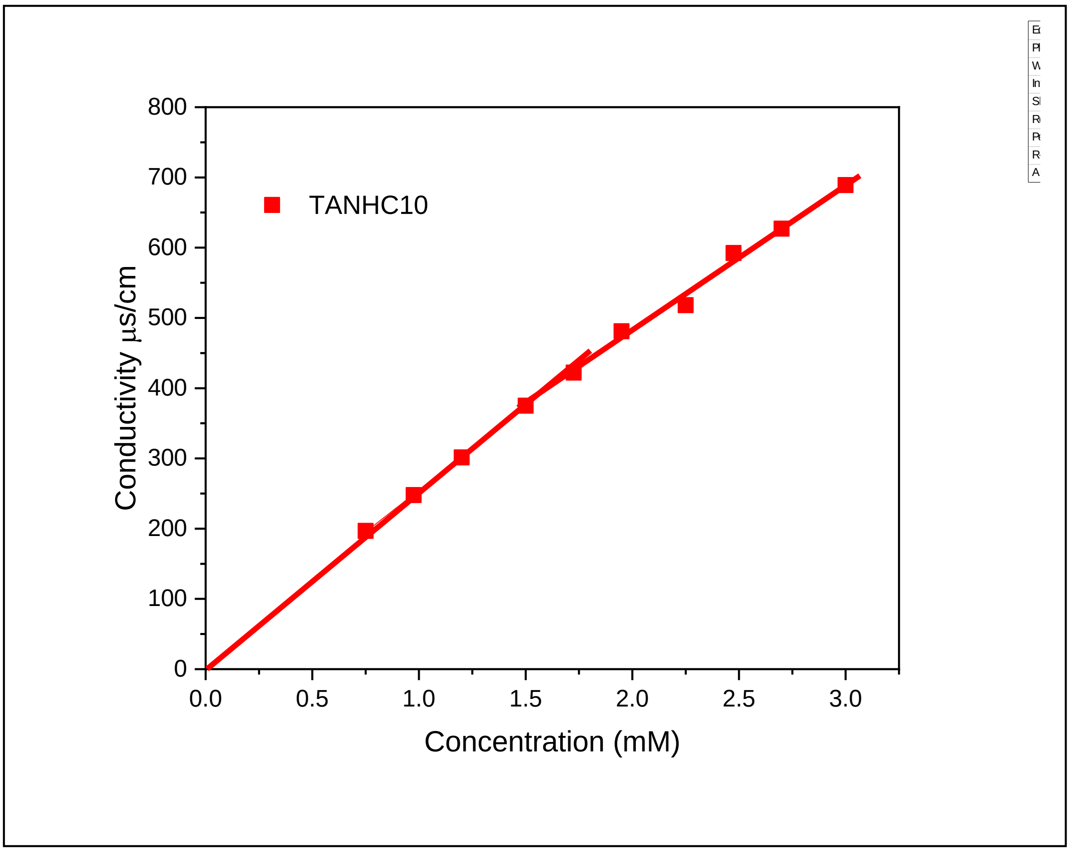
TANHC10
Conductivity µs/cm
Concentration (mM)
800
700
600
500
400
300
200
100
0
0.0
0.5
1.0
1.5
2.0
2.5
3.0

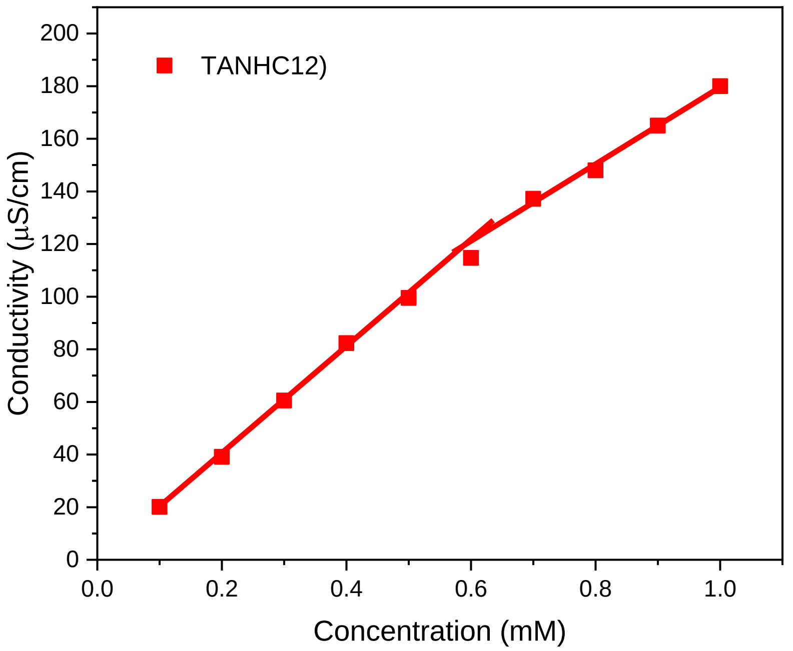
TANHC12)
Conductivity (μS/cm)
Concentration (mM)
0
20
40
60
80
100
120
140
160
180
200
0.0
0.2
0.4
0.6
0.8
1.0

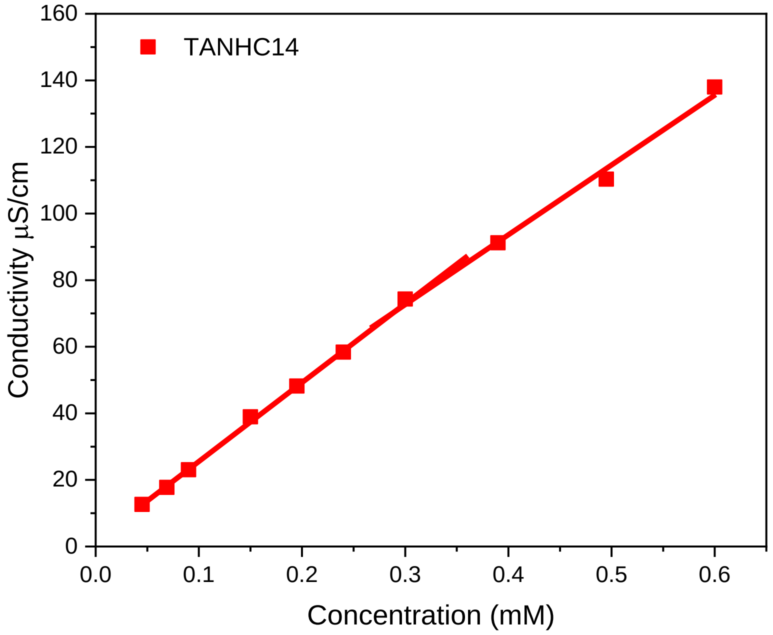
TANHC14
Conductivity μS/cm
Concentration (mM)
0
20
40
60
80
100
120
140
160
0.0
0.1
0.2
0.3
0.4
0.5
0.6

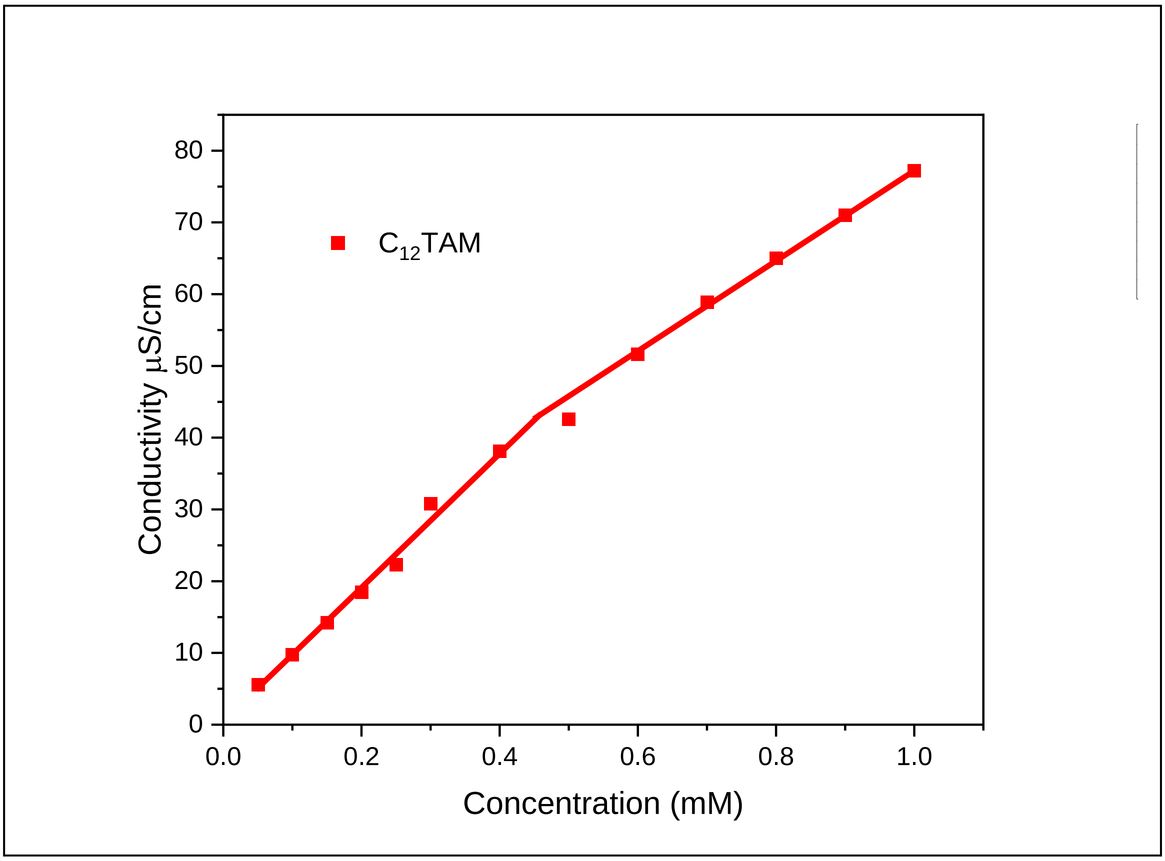

C12TAM
Conductivity µS/cm
Concentration (mM)
0
10
20
30
40
50
60
70
80
0.0
0.2
0.4
0.6
0.8
1.0

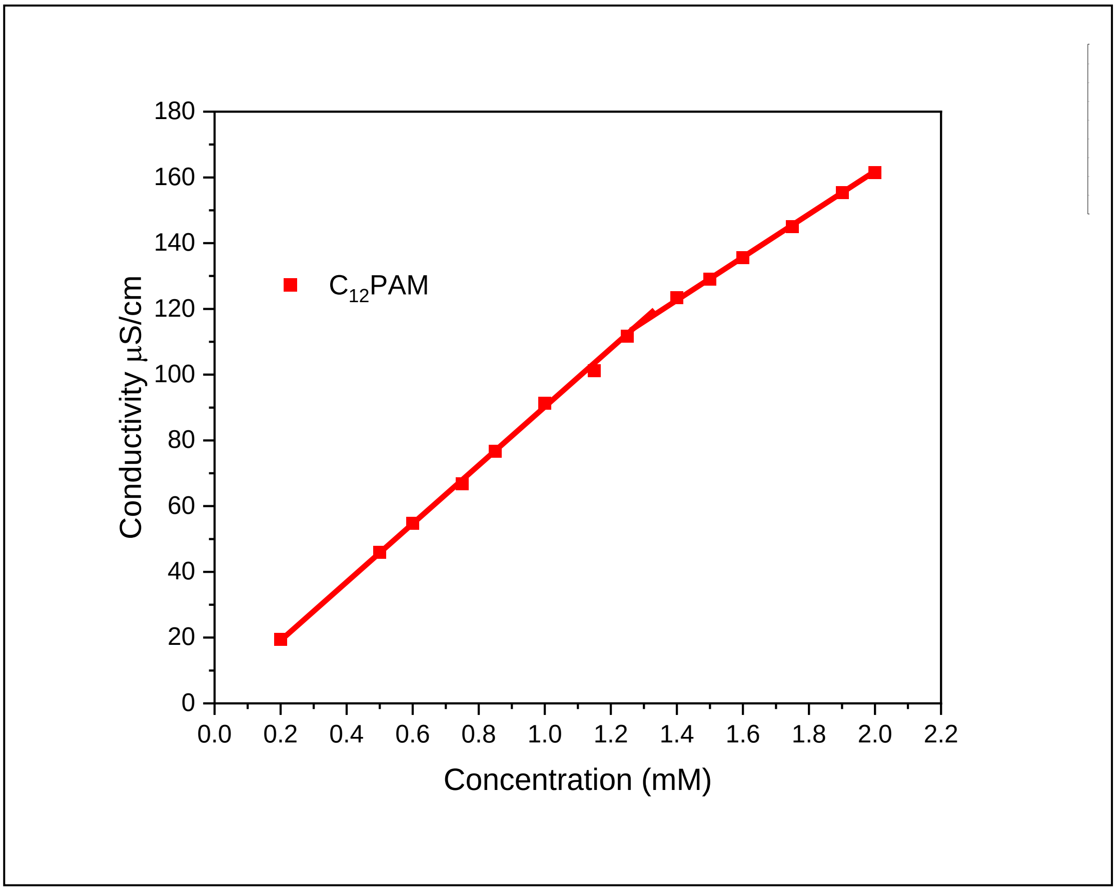

C12PAM
Conductivity µS/cm
Concentration (mM)
0
20
40
60
80
100
120
140
160
180
0.0
0.2
0.4
0.6
0.8
1.0
1.2
1.4
1.6
1.8
2.0
2.2

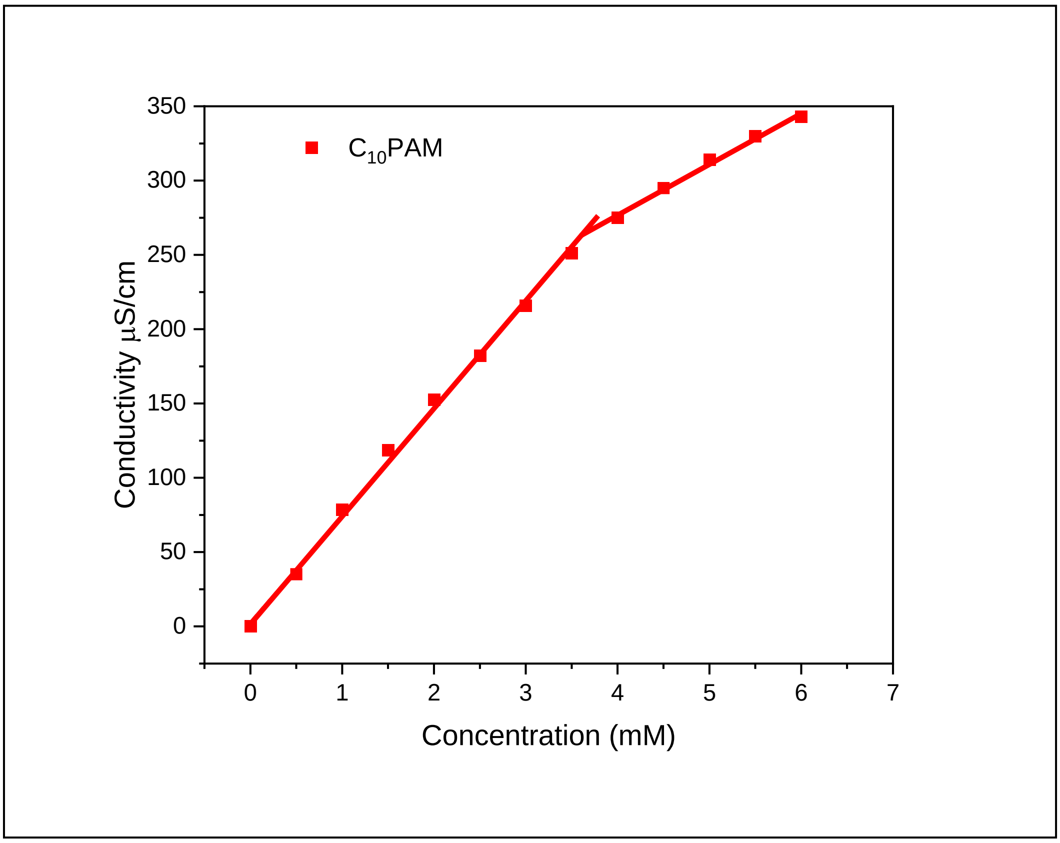

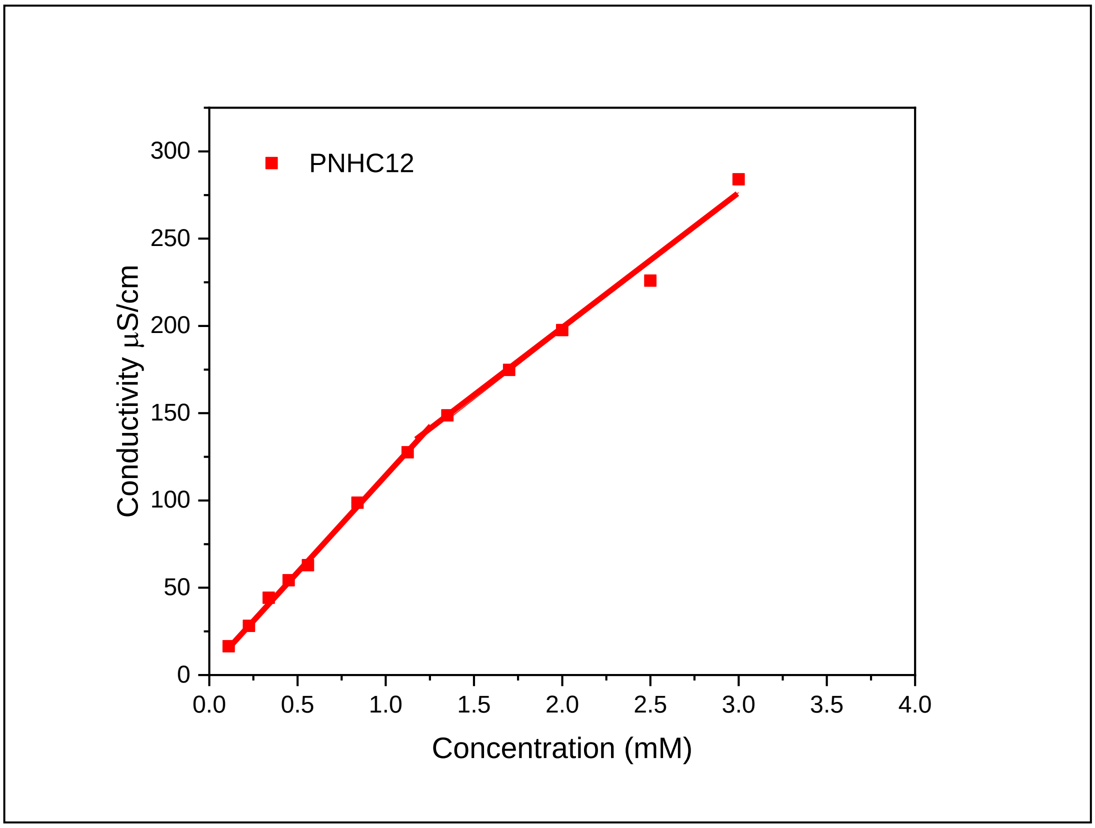

Figure S26. Specific conductivity-concentration curves for the amino acid-based surfactants.
